# Assessing the potential of polygenic scores to strengthen medical risk prediction models of COVID-19

**DOI:** 10.1371/journal.pone.0285991

**Published:** 2023-05-26

**Authors:** Aldo Córdova-Palomera, Csaba Siffel, Chris DeBoever, Emily Wong, Dorothée Diogo, Sandor Szalma

**Affiliations:** 1 Takeda Development Center Americas, Inc., San Diego, California, United States of America; 2 Takeda Development Center Americas, Inc., Cambridge, Massachusetts, United States of America; Girne American University - Karmi Campus: Girne Amerikan Universitesi, CYPRUS

## Abstract

As findings on the epidemiological and genetic risk factors for coronavirus disease-19 (COVID-19) continue to accrue, their joint power and significance for prospective clinical applications remains virtually unexplored. Severity of symptoms in individuals affected by COVID-19 spans a broad spectrum, reflective of heterogeneous host susceptibilities across the population. Here, we assessed the utility of epidemiological risk factors to predict disease severity prospectively, and interrogated genetic information (polygenic scores) to evaluate whether they can provide further insights into symptom heterogeneity. A standard model was trained to predict severe COVID-19 based on principal component analysis and logistic regression based on information from eight known medical risk factors for COVID-19 measured before 2018. In UK Biobank participants of European ancestry, the model achieved a relatively high performance (area under the receiver operating characteristic curve ~90%). Polygenic scores for COVID-19 computed from summary statistics of the Covid19 Host Genetics Initiative displayed significant associations with COVID-19 in the UK Biobank (*p*-values as low as 3.96e-9, all with *R*^2^ under 1%), but were unable to robustly improve predictive performance of the non-genetic factors. However, error analysis of the non-genetic models suggested that affected individuals misclassified by the medical risk factors (predicted low risk but actual high risk) display a small but consistent increase in polygenic scores. Overall, the results indicate that simple models based on health-related epidemiological factors measured years before COVID-19 onset can achieve high predictive power. Associations between COVID-19 and genetic factors were statistically robust, but currently they have limited predictive power for translational settings. Despite that, the outcomes also suggest that severely affected cases with a medical history profile of low risk might be partly explained by polygenic factors, prompting development of boosted COVID-19 polygenic models based on new data and tools to aid risk-prediction.

## Introduction

Individuals infected by the severe acute respiratory syndrome coronavirus 2 (SARS-CoV-2) develop coronavirus disease 2019 (COVID-19), which spans a broad set of clinical outcomes -from asymptomatic to critically ill with acute respiratory distress syndrome, sepsis, cardiac and immune complications that can be fatal [1]. The outcome of a COVID-19 infection depends on complex interactions between susceptibility risk factors, including healthcare access [2, 3], underlying medical conditions [4–6] and genetic risk factors [7, 8]. Some epidemiological, clinical and laboratory findings are now widely recognized to be involved in COVID-19 risk and progression [6], and growing evidence is unraveling their genetic underpinnings [7, 8]. Recent scientific efforts have started to develop a unified understanding of symptom severity reflective of host heterogeneities, with a clinical scope and potential for translational settings [7, 9, 10].

Converging evidence suggests that COVID-19 symptoms and severity can be successfully informed by machine learning models leveraging data sources such as diagnostic histories, medical imaging or natural language information [11–14]. With a myriad of choices in terms of data sources and analytical tools, models that combine correctness, interpretability and usability are of particular interest in clinical and translational settings [15–17]. Although a balanced trade-off between those three aspects is complicated, it might be possible through the application of clinical and machine learning expertise to investigate widely accessible biomedical data sources and protocols [18, 19].

Of particular interest in this regard are approaches combining medical comorbidities and genetic information, as the former have already shown promising results whereas genetics might help predict and interpret idiopathic, sporadic and other cases of unknown etiology [20–22]. Models incorporating genetic and nongenetic factors for hazard prediction across a variety of diseases have previously highlighted the clinical potential of multifactorial risk assessment, and outlined how the discrimination of variance obtained from health assessments and polygenes can provide valuable biological insights and parallelly provide substantial contributions to predictive power [23–25].

In this study we aim to test the utility of pre-morbid medical records and genetic data to prospectively inform on COVID-19 severity risk, through interpretable machine learning models. First, a curated set of 8 medical conditions known from meta-analyses or systematic reviews to increase the odds of severe COVID-19 were interrogated to build a base machine learning classifier to predict severe outcomes of COVID-19 infections in a harmonized set of 337,484 unrelated individuals of European ancestry in the UK Biobank. Next, polygenic scores (PS) for COVID-19 were computed in the UK Biobank using summary statistics of the Covid19 Host Genetics Initiative as reference. Associations between the PS and severe COVID-19 outcomes were quantified through statistical estimates including significance levels and effect sizes. Finally, we conjectured that individuals misclassified by the base model would show abnormal genetic burden for COVID-19 (e.g., an affected individual who is predicted healthy according to non-genetic factors would have a high genetic liability), and tested that hypothesis by error analysis of the machine learning predictions in relation to PS values.

## Materials and methods

### Participants and ethics statement

We utilized data from the UK Biobank (application 26041), a longitudinal cohort study of over 500,000 individuals aged 40–69 years enrolled between 2006 and 2019 in study centers across Wales, Scotland and England. Extensive baseline data on physical measurements, health behavior and medical history of participants were collected through questionnaires and clinical examination. In addition, saliva, urine and blood samples were collected from the participants. For the UK Biobank, ethical approval was granted by the North West Multi-Centre Research Ethics Committee and the National Health Service (NHS) National Research Ethics Service (ref: 11/NW/0382). Participants provided written informed consent to participate in the UK Biobank study, and all experiments were performed in agreement with relevant guidelines and regulations. Further details, including information on the study protocol, is available online at https://biobank.ctsu.ox.ac.uk/.

### Case-control definitions

COVID-19 laboratory test results, hospitalization and death records from the UK Biobank were accessed on March 19^th^, 2021. We limit the records to that date, in an attempt to avoid confounders related to access to vaccines and to different COVID-19 variants propagated more recently. Consistent with conventions used by the Covid19 Host Genetics Initiative working group (https://www.covid19hg.org), COVID-19-related data from the UK Biobank were used to obtain seven COVID-19 case-control definitions and these definitions were adopted based on severity of clinical outcomes: A1, lenient.A1 and A2 (very severe respiratory confirmed), B1 and B2 (hospitalized lab confirmed) and C1 and C2 (partial-susceptibility). More details on each case-control definition, as well as number of cases and controls in the UK Biobank at time of accession can be found in Table 1. Data and analyses presented next, correspond to the largest unrelated European set of participants in the UK Biobank (rightmost columns in Table 1), which had a maximum sample size of 337,484 after accounting for data completeness and relevant inclusion criteria.

**Table 1:** COVID-19 case-control definitions based on conventions from the Covid19 Host Genetics Initiative working group (https://www.covid19hg.org).

Definition	Case definition	Control definition	All UKB		Unrelated Eur UKB	
			N cases	N controls	N cases	N controls
A1 VERY SEVERE RESPIRATORY CONFIRMED COVID	Hospitalized laboratory confirmed SARS-CoV-2 infection AND death AND hospitalization with COVID19 as ++ primary reason ++ for admission	Laboratory confirmed SARS-CoV-2 infection AND not hospitalised ** 21 days after the test **	390	8487	278	5615
A1 VERY SEVERE RESPIRATORY CONFIRMED COVID (lenient)	Case if ever PCR positive AND [died with U071 or U072 code OR (died AND HES inpatient with diagnosis code U071 or U072)]	Ctrl if ever positive AND did not die with U071 nor U072 AND no evidence of hospitalization in 2020	627	8487	446	5615
A2 VERY SEVERE RESPIRATORY CONFIRMED COVID	Same as A1	Everyone that is not a case, i.e. the population	390	406647	278	287771
B1 HOSPITALIZED LAB CONFIRMED COVID	Hospitalized laboratory confirmed SARS-CoV-2 infection AND hospitalization due to corona-related symptoms.	Laboratory confirmed SARS-CoV-2 infection AND not hospitalised ** 21 days after the test **	1564	8487	1046	5615
B2 HOSPITALIZED LAB CONFIRMED COVID	Same as B1	Everyone that is not a case, i.e. the population	1564	405473	1046	287003
C1 PARTIAL-SUSCEPTIBILITY	Laboratory confirmation of SARS-CoV-2 infection OR EHR/ICD coding/ Physician Confirmed COVID-19 ** OR self-reported COVID-19 positive (e.g. by questionnaire)	(Laboratory tested for SARS-CoV-2 infection AND all tests (if multiple tests) negative*) OR self-reported tested negative for SARS-CoV-2 infection (e.g. by questionnaire)	14539	49819	9635	35469
C2 PARTIAL-SUSCEPTIBILITY	Same as C1	Everyone that is not a case, i.e. the population	14539	392498	9635	278414

### Medical risk factors

Medical information and general health-related trait data available through the UK Biobank showcase was curated through PHESANT [26], a dedicated software tool for large-scale UK Biobank phenome screenings implemented as an R package that provides an automated processing workflow to determine variable coding and standardization of continuous, integer and both single- and multi-value categorical fields.

A set of eight medical risk factors for severe COVID-19 was retrieved from data provided by the U.S. Centers for Disease Control and Prevention (CDC), using their first tier list (medical factors supported by meta-analysis/systematic review) (https://www.cdc.gov/coronavirus/2019-ncov/science/science-briefs/underlying-evidence-table.html, accessed on August 5^th^, 2021). Those eight risk factors were operationalized in UK Biobank fields (https://biobank.ctsu.ox.ac.uk/crystal/browse.cgi) recorded before 2019 as follows: cancer (C*, and D0* to D4* ICD-10 codes), cerebrovascular disease (I6* codes), chronic kidney disease (N18* codes), chronic obstructive pulmonary disease (COPD; J44* codes, or a COPD diagnosis by a doctor and age it was diagnosed, recorded through an online questionnaire), type 1 and type 2 diabetes mellitus (E08* to E13* codes), heart conditions such as heart failure, coronary artery disease or cardiomyopathies (I50*, I25* and I42*) and obesity (body mass index, entered as continuous measures from UKB data fields 21001 and 23104). A total of 501 data fields (columns) accounted for all previous variables; the complete catalog of fields, including summary information for each of them, is provided as S1 Table in S1 File.

### Machine learning classification model

An initial predictive model for COVID-19 was implemented using information related to the eight major risk factors listed above, represented in five hundred and one possibly correlated variables, together with age at recruitment and sex were prepared to be entered into a base machine learning classifier predicting case-control status. For the sake of interpretability, and to account for missingness and correlations between features, the baseline classifier consisted of a missing value imputer to the most frequent values, followed by dimensionality reduction by PCA (up to 50 components) and logistic regression solved by limited-memory BFGS (lbfgs) with up to 100 iterations. Noting that gradient boosting models might have better performance for classification tasks with tabular input data [27], the output of the logistic regression model above was compared against XGBoost when relevant. Models were fit through 10-fold cross validation with a fixed random seed, and correctness was evaluated through area under the receiver operating characteristic curve (AUROC) and confusion matrices.

### Estimation of polygenic scores and associations with COVID-19

The fifth round of COVID-19 genome-wide association study (GWAS) meta-analysis summary statistics performed by the Covid19 Host Genetics Initiative was downloaded from the public release (https://www.covid19hg.org/results/r5/, date of release: January 18^th^, 2021). Eight files were retrieved, corresponding to GWAS of four case-control definitions (A2, B1, B2 and C2; details in Table 1) in two cohorts (multi-ethnic and its European-ancestry subset); UK Biobank was not included in those studies. Each of those eight summary statistics files was applied to the UK Biobank using all seven case-control definitions, therefore giving a total of 56 PS analyses.

PS were computed for European-ancestry participants using the PRSice-2 toolbox [28] with standard parameters: clumping distance of 250kb with *R*^2^ threshold of 0.1 and *p*-value of 1, a step-size interval of 5e-05, and *p*-value thresholds ranging between 5e-08 and 0.5. For model fit assessment, sex, age and two genetic principal components were included to control for cryptic relatedness and population stratification. Estimates of *p*-values and variance explained (*R*^2^) are provided in the Results section. When PS were computed, the case-control labels were considered. The best fitting PS from each of the 56 analyses was retrieved for downstream analyses of the machine learning classifier.

### Polygenic scores for classifier improvement and error analysis

Fifty-six PS features, computed as detailed above, were used as follow-up on the initial classifier to try to improve its performance (as additional input features) or to conduct error analysis (e.g., to determine whether an affected individual who is incorrectly predicted healthy according to non-genetic factors would have a high genetic liability). When appropriate, analyses using PS estimates also included the first ten genetic principal components. Associations between PS estimates and predicted probability of disease in affected individuals were tested using univariate linear regression analysis including an intercept term, fit with ordinary least squares from Python’s statsmodel module [29].

## Results and discussion

### Machine learning classification based on medical risk factors

**Fig 1:**
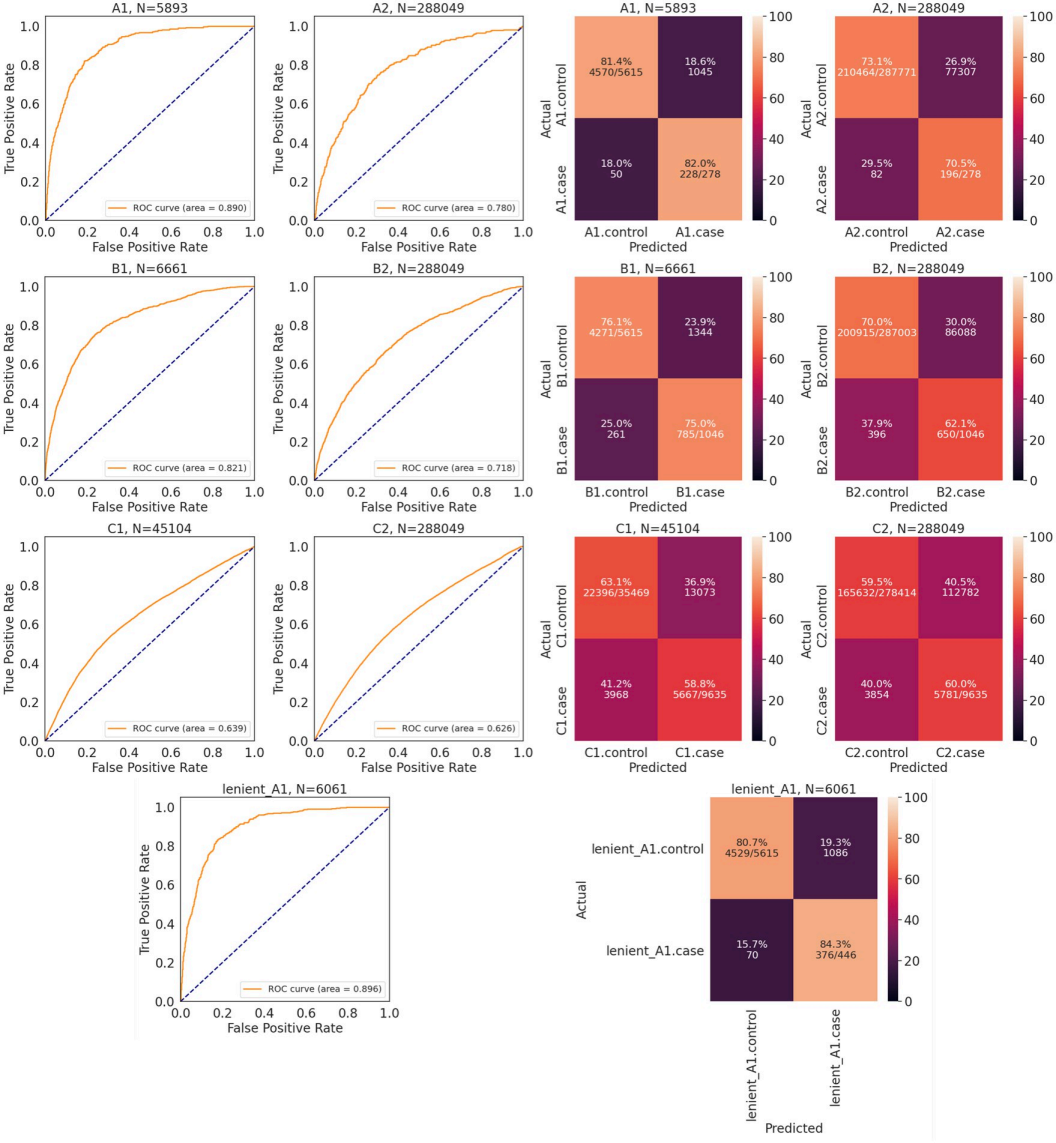
Receiver-operating characteristic curve and confusion matrices for the COVID-19 case-control classifiers using logistic regression.

Machine learning classifiers with information on eight medical risk factors, age and sex (initially embedded within five hundred and three sparse features) for severe COVID-19 were included in the principal component analysis (PCA) and logistic regression, and achieved performances between 62.6% and 89.6% AUROC for each of the seven case-control definitions using PCA plugged to logistic regression, and AUROC values between 64.4% and 89.1% with XGBoost. Since both logistic regression and XGBoost sets of models achieved similar performances across each scenario (Fig 1 and S1 Fig in S1 File), despite XGBoost having built-in capabilities to handle missingness, sparsity and feature interactions [30], results moving forward refer to the logistic regression model unless otherwise specified. Interestingly, although less severe case definitions have higher sample sizes (C2 > C1 > B2 > B1 > lenient.A1 > A2 > A1; Table 1), models comparing more severe COVID-19 outcomes against recovered or otherwise unaffected individuals (e.g., A1) showed generally higher AUROC values and higher weights on the confusion matrix diagonals than the more relaxed case-control definitions (e.g., B2, C1, C2). This is in line with the observation that individuals with multiple comorbidities are more likely to present severe COVID-19 outcomes [31], whereas less severe disease outcomes could be influenced by a few risk factors in isolation, populating a sparser feature matrix that can make predictions harder and less accurate.

Additionally, we evaluated the potential confounding impact of healthcare access on the risk predictions obtained above using medical factors. More specifically, we investigated whether date of disease onset was correlated with false negatives: individuals predicted to have lower risk of COVID-19 based on their medical history, but were actually affected by COVID-19. Results on S2, S3 Figs and S4 Table in S1 File do not provide conclusive evidence of a link between COVID-19 complications for individuals with low susceptibility based on their medical risk factors. However, it is worth noting that, for all case-control definitions, the association slopes display the hypothesized direction: an enrichment in subjects displaying COVID-19 complications near the weeks of pandemic peaks, even though those subjects do not have the standard medical risk profile characterized by the machine learning model.

Namely, for some case-control definitions, individuals whose disease onsets occurred during the early pandemic period had higher probability of being misclassified, ostensibly indicating that they did not necessarily have severe medical comorbidities but may have developed complications due to different healthcare practices and accessibility. Of note, despite the nominally- and trend-significant results for C1 (*p* = 0.018), C2 (*p* = 0.023) and B2 (*p* = 0.08) (S4 Table in S1 File), none of the associations between onset time and misclassification likelihood displayed a strong effect size nor remained statistically significant after adjustment for multiple comparisons.

### Polygenic score fitting

**Fig 2:**
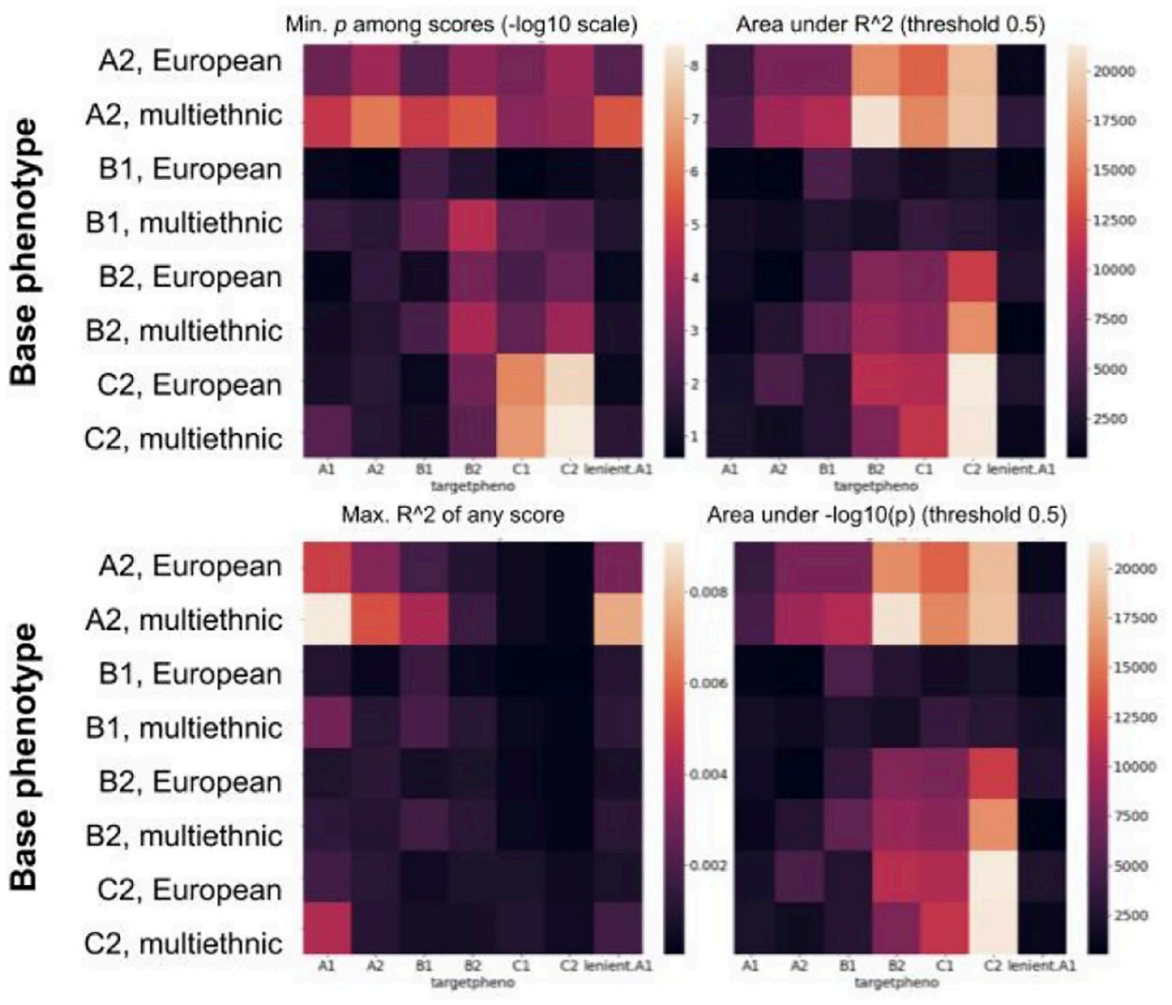
Summary of *R*^2^ and *p*-values for the 56 sets of PS analyses. “Base phenotype” refers to the case-control definition (e.g., A1) and ethnicity (e.g., European or multi-ancestry) used to compute the external GWAS summary statistics (excluding the UK Biobank cohort), whereas “targetpheno” indicates the case-control definition used for the current analysis of European-ancestry individuals in the UK Biobank.

Consistency of the UK Biobank genetic and phenotypic data with previously published COVID-19 studies was reviewed by genetic association tests across the seven case-control definitions, for the two loci reported by The Severe Covid-19 GWAS Group and Shelton and colleagues [8, 10] (Table 2). For the PS analysis, the initial stage consisted in computing score estimates for each of the seven case-control definitions, using eight base summary statistics from the Covid19 Host Genetics Initiative working group (https://www.covid19hg.org). Fig 2 and S2 Table in S1 File show statistics from each of the 56 PS models. Variance explained estimates for all PSs were under 1% (*R*^2^ between 2.7e-5 and 0.009), suggesting that the PSs are explaining only a small fraction of variance of disease status. Nominal *p*-values ranged between 3.69e-9 and 0.26, and 20 out of the 56 p-values remained statistically significant at *p*<0.05 after Bonferroni-based multiple testing adjustment. Beta coefficients for all 20 significant test results were in the expected direction (higher PS resulting in higher odds of COVID-19), whereas only 6 of the remaining 36 non-significant estimates were negative. B2 and C2 COVID-19 case-control definitions had the highest proportions of significant results after multiple testing correction (6/7 and 5/7), whereas the most stringent traits (A1, lenient.A1, A2 and B2) showed the lowest fractions of significant results. Although the latter result could be reflective of differences in number of participants across each case-control definition (sample sizes are considerably larger for non-stringent definitions such as C1 and C2 than for dead or hospitalized patients in A1 and A2), we also observe that common genetic factors might lack power to predict a severe outcome (such as death following a positive COVID-19 test result), consistent with the tangential observation that common genetic variants at 3p21.31 and 9q34.2 (ABO) previously related to COVID-19 in independent samples display stronger evidence of association with B1/B2/C1/C2 than with A1/A2/lenient A1 (S2 Table in S1 File) [8, 10].

**Table 2:** Results of genetic association tests across the seven case-control definitions, for the two loci reported by The Severe Covid-19 GWAS group and Shelton and colleagues [8, 10]. Analysis was restricted to the largest set of unrelated European participants in the UK Biobank.

Locus	Trait	ID	A1	AX	CASE_ALLELE_CT	CTRL_ALLELE_CT	OBS_CT	BETA	SE	P	Signif.
**3p21.31**	A1	rs11385942	GA	G	300	6274	3287	0.225506	0.22354	0.313075	
	A2	rs11385942	GA	G	300	575798	288049	0.257813	0.205498	0.209631	
	B1	rs11385942	GA	G	970	6274	3622	0.374012	0.128943	0.003724	*
	B2	rs11385942	GA	G	970	575128	288049	0.392026	0.10832	0.000296	*
	C1	rs11385942	GA	G	10846	49956	30401	0.075853	0.041555	0.067948	
	C2	rs11385942	GA	G	10846	565252	288049	0.07128	0.037247	0.055654	
**9q34.2 (ABO)**	A1	rs687621	G	A	300	6274	3287	0.101934	0.131954	0.439821	
	A2	rs687621	G	A	300	575798	288049	0.117967	0.121348	0.33098	
	B1	rs687621	G	A	970	6274	3622	0.00158	0.07884	0.984012	
	B2	rs687621	G	A	970	575128	288049	0.07433	0.068029	0.274559	
	C1	rs687621	G	A	10846	49956	30401	0.081449	0.022849	0.000364	*
	C2	rs687621	G	A	10846	565252	288049	0.091888	0.020494	7.34E-06	***

Among all base summary statistics tables, the one computed for the A2 trait using multi-ancestry individuals achieved the top-performing PS (smallest *p*-values and largest *R*^2^ estimates) for severe COVID-19 in the UK Biobank (A1, lenient.A1, A2, B1 and B2), whereas the multi-ancestry C2 base table was the top performer for C1 and C2 in the UK Biobank, which is likely indicative of phenotypic consistence between the base and target datasets.

### Analysis of machine learning model errors using polygenic scores

**Fig 3:**
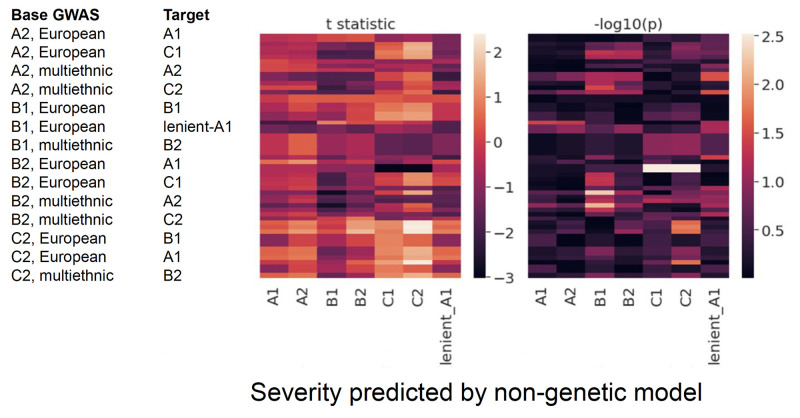
Linear regression results for the associations between 56 top polygenic scores and disease risk prediction based on medical comorbidities, among cases (not controls).

**Fig 4:**
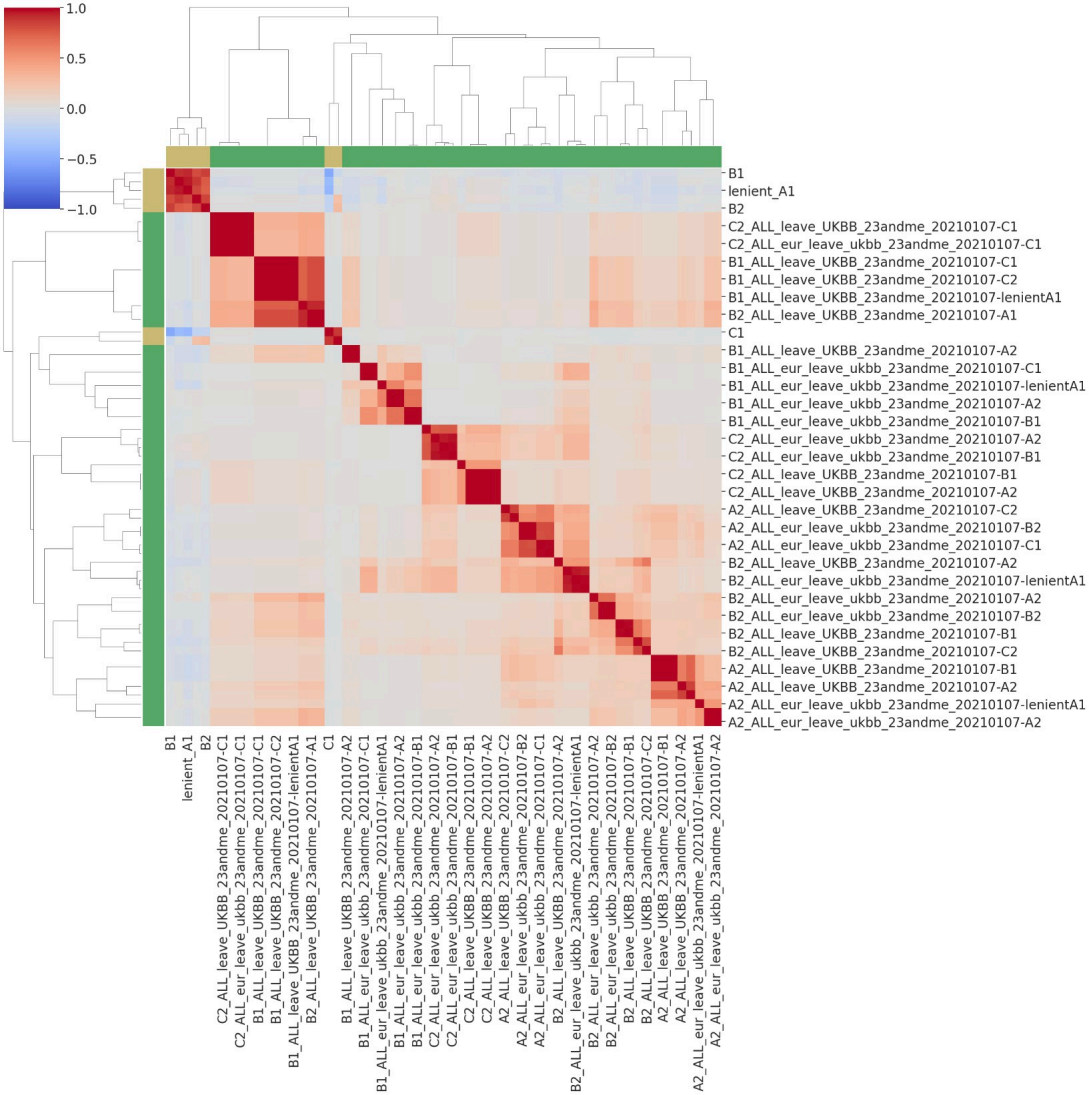
Correlations matrix of model predictions (case probabilities for A1, lenient A1, A2, B1, B2, C1 and C2 (yellow side bars)) and candidate polygenic scores (green side bars). For this matrix, predicted case probabilities were included only for true cases, whereas all available polygenic scores were used.

Next, in recognition that affected COVID-19 individuals who were misclassified as healthy based on (a lack of) medical comorbidities might have different biological drivers of outcomes, we hypothesized that their symptoms would be partly explained by genetic predisposition. To test this hypothesis, error analysis was performed for each case-control definition by extracting the subset of affected individuals and examining whether a statistically significant association existed between different polygenic measures of genetic risk and the predicted probability of disease by the medical (non-genetic) models. Three hundred and ninety-two regression statistics results were calculated (56 polygenic scores times 7 case definitions); *t* statistics ranged between -2.9 and 2.4, and *p*-values were between 0.004 and 0.999 (Fig 3, S3 Table and S4 Fig in S1 File). Of note, the set of *t* statistics were normally distributed and shifted to the left, with 267/392 of estimates having a negative sign (one sample *t*-test statistic (two-sided): -8.3, *p* = 1.47e-15; binomial (sign) test: *p* = 5.99e-13 for a 68% prob. of success, 95% confidence interval bounds at 63% and 73%). Although the set of *t* statistics are not completely independent (which likely inflated the latter test results) (Fig 4), the observation suggests that, in general, individuals with severe COVID-19 predicted as “low-risk” based on their medical history could actually have a higher genetic risk for disease. While the current evidence is not conclusive, it may indicate that further polygenic information (e.g., boosted polygenic scores) could be informative.

It is also worth mentioning that specific risk factors seem to drive the predictive power of the different classification models. To illustrate this point, S5 Fig in S1 File shows feature importance plots from the XGBoost model version (without the dimensionality reduction aspect of PCA concatenated to logistic regression). The figure suggests age as one of the main predictors; interestingly, younger age was enriched among controls in the severe phenotype definitions (A1, A2, lenient A1, B1 and B2), whereas younger age also correlated with higher risk for the less severe phenotypes (C1 and C2). This may indicate a pattern where, although young people could be more exposed to infections (leading to C1 and C2 case phenotypes), they would also be less likely to have drastic complications (A1, A2, lenient A1, B1 and B2 cases). Among the remaining medical factors with high importance according to S5 Fig in S1 File, gender is also present across most of the analyses, with females having lower overall prevalence of complications. Lower body mass index (based on UK Biobank field 21001) also correlated with lower risk of symptoms; the related data field 23104 showed a similar trend. A diagnosis of COPD (indexed by field 22130), and vitamin D (recorded through field 100021) were also among the top predictors across phenotype definitions.

### Study limitations

Some limitations of this study deserve mention. First, since the UK Biobank is neither a hospital-based sample nor a cohort reflective of the underlying sociodemographic and health distributions in the UK, disease prevalences and clinical outcomes might not fully reflect observations in the general population. In addition, we cannot rule out the possibility that the associations between polygenic scores and disease severity might have been mediated by intermediate phenotypes or medical risk factors unaccounted for in the current research setting. Similarly, the case/control overlap between phenotype definitions might have caused some similarity between sets of results. Novel research aimed at determining additional health susceptibilities for COVID-19 and decomposition of COVID-19 polygenic risk into phenotypic dimensions might help uncover to what extent the disease severity PS might be driven by genetic risk for other conditions.

## Conclusions

Overall, the findings are fourfold. First, the results agree with previous reports in that a few well-known medical risk factors can make a relatively robust prediction of COVID-19 risk, in line with earlier genetic risk results [16, 31], and with a more recent publication by Nakanishi and colleagues [32]. Second, medical risk factors show stronger power to discriminating very severe COVID-19 cases from infected but recovered controls, whereas their predictive performance is worse as case-control definitions get relaxed. We notice that this could reflect a multifactorial pattern of pre-existing comorbidities in individuals with worse clinical outcomes [31], whereas subjects who recover promptly may have a less clear profile with fewer and less severe risk factors that may or may not end up causing severe disease. In that regard, soft trait definitions (e.g., C1/C2) might include individuals with only a few risk predictors in both case and control groups, complicating discrimination. Additionally, more relaxed definitions (C1/C2) could be more prone to phenotyping errors since severity of symptoms can be low in both cases and controls. The seemingly paradoxical fact that severe COVID-19 phenotypes can be effectively discriminated by established medical risk factors, whereas the best polygenic score fittings are observed for non-severe outcomes, can probably be attributed to the availability of a well-powered set of medical variables in the former case (which would work best at predicting critical illnesses but not mild symptoms) whereas the latter scenario might reflect higher statistical power of GWAS for broadly-defined COVID-19 infection. Third, polygenic scores for COVID-19 are statistically associated with disease in the current sample, but, due to their low variance explained estimates (largest *R*^2^<1%), they did not provide additional power to improve discrimination between cases and controls. That is in line with previous reports suggesting a moderate (<1%) but statistically significant COVID-19 heritability estimation based on existing GWAS data [33] and suggests that, in their current form, polygenic scores based on common variants might show limited clinical utility. Fourth, despite the latter observation, we observe a small but consistent increase in polygenic risk estimates in COVID-19 cases misclassified by non-genetic factors (affected individuals with a medical history profile of relatively low risk), which could indicate that boosted PSs could have more power and potentially be more relevant in translational settings. Research in the active field of genetic risk calculation [20–22] may help develop tools to address this scenario.

## Supporting information

S1 File Supporting figures and tables. (DOCX)
