# Correction: Assessing the potential of polygenic scores to strengthen medical risk prediction models of COVID-19

**DOI:** 10.1371/journal.pone.0326209

**Published:** 2025-06-10

**Authors:** Aldo Córdova-Palomera, Csaba Siffel, Chris DeBoever, Emily Wong, Dorothée Diogo, Sandor Szalma

The affiliation for the second author is incorrect. Csaba Siffel is not affiliated with #1 but with #2: Takeda Development Center Americas, Inc., Cambridge, Massachusetts, United States of America.
