# Supplementary material for: Assessing the potential of polygenic scores to strengthen medical risk prediction models of COVID-19
Source: PLoS One. 2023 May 26;18(5):e0285991. doi: 10.1371/journal.pone.0285991 (PMC10218741; doi:10.1371/journal.pone.0285991)
Supplement: S1 File — (DOCX) [file pone.0285991.s001.docx]

***Supplementary Material***

The following information refers to the manuscript ***Assessing the Potential of Polygenic Scores to Strengthen Medical Risk Prediction Models of COVID-19***.

| ***Item*** | ***Page*** |
| --- | --- |
| Supplementary Table S1 | 2 |
| Supplementary Figure S1 | 21 |
| Supplementary Table S2 | 22 |
| Supplementary Table S3 | 24 |
| Supplementary Figure S2 | 35 |
| Supplementary Figure S3 | 36 |
| Supplementary Table S4 | 37 |
| Supplementary Figure S4 | 38 |
| Supplementary Figure S5 | 39 |

| code | name | category | category_id |
| --- | --- | --- | --- |
| 21001 | Body mass index (BMI) | UK Biobank Assessment Centre > Physical measures > Anthropometry > Body size measures | 100010 |
| 22130 | Doctor diagnosed COPD (chronic obstructive pulmonary disease) | Online follow-up > Work environment > Medical information | 132 |
| 22150 | Age COPD (Chronic Obstructive Pulmonary Disease) diagnosed by doctor | Online follow-up > Work environment > Medical information | 132 |
| 23104 | Body mass index (BMI) | UK Biobank Assessment Centre > Physical measures > Anthropometry > Impedance measures | 100009 |
| 40001_C159 | Underlying (primary) cause of death: ICD10-C15.9 Oesophagus, unspecified | Health-related outcomes > Death register | 100093 |
| 40001_C169 | Underlying (primary) cause of death: ICD10-C16.9 Stomach, unspecified | Health-related outcomes > Death register | 100093 |
| 40001_C189 | Underlying (primary) cause of death: ICD10-C18.9 Colon, unspecified | Health-related outcomes > Death register | 100093 |
| 40001_C19 | Underlying (primary) cause of death: ICD10-C19 Malignant neoplasm of rectosigmoid junction | Health-related outcomes > Death register | 100093 |
| 40001_C20 | Underlying (primary) cause of death: ICD10-C20 Malignant neoplasm of rectum | Health-related outcomes > Death register | 100093 |
| 40001_C220 | Underlying (primary) cause of death: ICD10-C22.0 Liver cell carcinoma | Health-related outcomes > Death register | 100093 |
| 40001_C221 | Underlying (primary) cause of death: ICD10-C22.1 Intrahepatic bile duct carcinoma | Health-related outcomes > Death register | 100093 |
| 40001_C259 | Underlying (primary) cause of death: ICD10-C25.9 Pancreas, unspecified | Health-related outcomes > Death register | 100093 |
| 40001_C260 | Underlying (primary) cause of death: ICD10-C26.0 Intestinal tract, part unspecified | Health-related outcomes > Death register | 100093 |
| 40001_C349 | Underlying (primary) cause of death: ICD10-C34.9 Bronchus or lung, unspecified | Health-related outcomes > Death register | 100093 |
| 40001_C439 | Underlying (primary) cause of death: ICD10-C43.9 Malignant melanoma of skin, unspecified | Health-related outcomes > Death register | 100093 |
| 40001_C459 | Underlying (primary) cause of death: ICD10-C45.9 Mesothelioma, unspecified | Health-related outcomes > Death register | 100093 |
| 40001_C509 | Underlying (primary) cause of death: ICD10-C50.9 Breast, unspecified | Health-related outcomes > Death register | 100093 |
| 40001_C541 | Underlying (primary) cause of death: ICD10-C54.1 Endometrium | Health-related outcomes > Death register | 100093 |
| 40001_C56 | Underlying (primary) cause of death: ICD10-C56 Malignant neoplasm of ovary | Health-related outcomes > Death register | 100093 |
| 40001_C61 | Underlying (primary) cause of death: ICD10-C61 Malignant neoplasm of prostate | Health-related outcomes > Death register | 100093 |
| 40001_C64 | Underlying (primary) cause of death: ICD10-C64 Malignant neoplasm of kidney, except renal pelvis | Health-related outcomes > Death register | 100093 |
| 40001_C679 | Underlying (primary) cause of death: ICD10-C67.9 Bladder, unspecified | Health-related outcomes > Death register | 100093 |
| 40001_C719 | Underlying (primary) cause of death: ICD10-C71.9 Brain, unspecified | Health-related outcomes > Death register | 100093 |
| 40001_C80 | Underlying (primary) cause of death: ICD10-C80 Malignant neoplasm without specification of site | Health-related outcomes > Death register | 100093 |
| 40001_C800 | Underlying (primary) cause of death: ICD10-C80.0 Malignant neoplasm, primary site unknown, so stated | Health-related outcomes > Death register | 100093 |
| 40001_C809 | Underlying (primary) cause of death: ICD10-C80.9 Malignant neoplasm, unspecified | Health-related outcomes > Death register | 100093 |
| 40001_C859 | Underlying (primary) cause of death: ICD10-C85.9 Non-Hodgkin's lymphoma, unspecified type | Health-related outcomes > Death register | 100093 |
| 40001_C900 | Underlying (primary) cause of death: ICD10-C90.0 Multiple myeloma | Health-related outcomes > Death register | 100093 |
| 40001_C920 | Underlying (primary) cause of death: ICD10-C92.0 Acute myeloid leukaemia | Health-related outcomes > Death register | 100093 |
| 40001_I251 | Underlying (primary) cause of death: ICD10-I25.1 Atherosclerotic heart disease | Health-related outcomes > Death register | 100093 |
| 40001_I259 | Underlying (primary) cause of death: ICD10-I25.9 Chronic ischaemic heart disease, unspecified | Health-related outcomes > Death register | 100093 |
| 40001_I609 | Underlying (primary) cause of death: ICD10-I60.9 Subarachnoid haemorrhage, unspecified | Health-related outcomes > Death register | 100093 |
| 40001_I619 | Underlying (primary) cause of death: ICD10-I61.9 Intracerebral haemorrhage, unspecified | Health-related outcomes > Death register | 100093 |
| 40001_I64 | Underlying (primary) cause of death: ICD10-I64 Stroke, not specified as haemorrhage or infarction | Health-related outcomes > Death register | 100093 |
| 40001_J440 | Underlying (primary) cause of death: ICD10-J44.0 Chronic obstructive pulmonary disease with acute lower respiratory infection | Health-related outcomes > Death register | 100093 |
| 40001_J449 | Underlying (primary) cause of death: ICD10-J44.9 Chronic obstructive pulmonary disease, unspecified | Health-related outcomes > Death register | 100093 |
| 40002_C349 | Contributory (secondary) causes of death: ICD10-C34.9 Bronchus or lung, unspecified | Health-related outcomes > Death register | 100093 |
| 40002_C61 | Contributory (secondary) causes of death: ICD10-C61 Malignant neoplasm of prostate | Health-related outcomes > Death register | 100093 |
| 40002_C780 | Contributory (secondary) causes of death: ICD10-C78.0 Secondary malignant neoplasm of lung | Health-related outcomes > Death register | 100093 |
| 40002_C787 | Contributory (secondary) causes of death: ICD10-C78.7 Secondary malignant neoplasm of liver | Health-related outcomes > Death register | 100093 |
| 40002_C793 | Contributory (secondary) causes of death: ICD10-C79.3 Secondary malignant neoplasm of brain and cerebral meninges | Health-related outcomes > Death register | 100093 |
| 40002_C795 | Contributory (secondary) causes of death: ICD10-C79.5 Secondary malignant neoplasm of bone and bone marrow | Health-related outcomes > Death register | 100093 |
| 40002_C798 | Contributory (secondary) causes of death: ICD10-C79.8 Secondary malignant neoplasm of other specified sites | Health-related outcomes > Death register | 100093 |
| 40002_C80 | Contributory (secondary) causes of death: ICD10-C80 Malignant neoplasm without specification of site | Health-related outcomes > Death register | 100093 |
| 40002_E119 | Contributory (secondary) causes of death: ICD10-E11.9 Without complications | Health-related outcomes > Death register | 100093 |
| 40002_I251 | Contributory (secondary) causes of death: ICD10-I25.1 Atherosclerotic heart disease | Health-related outcomes > Death register | 100093 |
| 40002_I259 | Contributory (secondary) causes of death: ICD10-I25.9 Chronic ischaemic heart disease, unspecified | Health-related outcomes > Death register | 100093 |
| 40002_I500 | Contributory (secondary) causes of death: ICD10-I50.0 Congestive heart failure | Health-related outcomes > Death register | 100093 |
| 40002_I501 | Contributory (secondary) causes of death: ICD10-I50.1 Left ventricular failure | Health-related outcomes > Death register | 100093 |
| 40002_I509 | Contributory (secondary) causes of death: ICD10-I50.9 Heart failure, unspecified | Health-related outcomes > Death register | 100093 |
| 40002_I64 | Contributory (secondary) causes of death: ICD10-I64 Stroke, not specified as haemorrhage or infarction | Health-related outcomes > Death register | 100093 |
| 40002_I679 | Contributory (secondary) causes of death: ICD10-I67.9 Cerebrovascular disease, unspecified | Health-related outcomes > Death register | 100093 |
| 40002_J449 | Contributory (secondary) causes of death: ICD10-J44.9 Chronic obstructive pulmonary disease, unspecified | Health-related outcomes > Death register | 100093 |
| 40002_N189 | Contributory (secondary) causes of death: ICD10-N18.9 Chronic renal failure, unspecified | Health-related outcomes > Death register | 100093 |
| 40006_C01 | Type of cancer: ICD10-C01 Malignant neoplasm of base of tongue | Health-related outcomes > Cancer register | 100092 |
| 40006_C029 | Type of cancer: ICD10-C02.9 Tongue, unspecified | Health-related outcomes > Cancer register | 100092 |
| 40006_C07 | Type of cancer: ICD10-C07 Malignant neoplasm of parotid gland | Health-related outcomes > Cancer register | 100092 |
| 40006_C099 | Type of cancer: ICD10-C09.9 Tonsil, unspecified | Health-related outcomes > Cancer register | 100092 |
| 40006_C155 | Type of cancer: ICD10-C15.5 Lower third of oesophagus | Health-related outcomes > Cancer register | 100092 |
| 40006_C159 | Type of cancer: ICD10-C15.9 Oesophagus, unspecified | Health-related outcomes > Cancer register | 100092 |
| 40006_C160 | Type of cancer: ICD10-C16.0 Cardia | Health-related outcomes > Cancer register | 100092 |
| 40006_C169 | Type of cancer: ICD10-C16.9 Stomach, unspecified | Health-related outcomes > Cancer register | 100092 |
| 40006_C180 | Type of cancer: ICD10-C18.0 Caecum | Health-related outcomes > Cancer register | 100092 |
| 40006_C181 | Type of cancer: ICD10-C18.1 Appendix | Health-related outcomes > Cancer register | 100092 |
| 40006_C182 | Type of cancer: ICD10-C18.2 Ascending colon | Health-related outcomes > Cancer register | 100092 |
| 40006_C183 | Type of cancer: ICD10-C18.3 Hepatic flexure | Health-related outcomes > Cancer register | 100092 |
| 40006_C184 | Type of cancer: ICD10-C18.4 Transverse colon | Health-related outcomes > Cancer register | 100092 |
| 40006_C185 | Type of cancer: ICD10-C18.5 Splenic flexure | Health-related outcomes > Cancer register | 100092 |
| 40006_C186 | Type of cancer: ICD10-C18.6 Descending colon | Health-related outcomes > Cancer register | 100092 |
| 40006_C187 | Type of cancer: ICD10-C18.7 Sigmoid colon | Health-related outcomes > Cancer register | 100092 |
| 40006_C189 | Type of cancer: ICD10-C18.9 Colon, unspecified | Health-related outcomes > Cancer register | 100092 |
| 40006_C19 | Type of cancer: ICD10-C19 Malignant neoplasm of rectosigmoid junction | Health-related outcomes > Cancer register | 100092 |
| 40006_C20 | Type of cancer: ICD10-C20 Malignant neoplasm of rectum | Health-related outcomes > Cancer register | 100092 |
| 40006_C220 | Type of cancer: ICD10-C22.0 Liver cell carcinoma | Health-related outcomes > Cancer register | 100092 |
| 40006_C221 | Type of cancer: ICD10-C22.1 Intrahepatic bile duct carcinoma | Health-related outcomes > Cancer register | 100092 |
| 40006_C250 | Type of cancer: ICD10-C25.0 Head of pancreas | Health-related outcomes > Cancer register | 100092 |
| 40006_C259 | Type of cancer: ICD10-C25.9 Pancreas, unspecified | Health-related outcomes > Cancer register | 100092 |
| 40006_C320 | Type of cancer: ICD10-C32.0 Glottis | Health-related outcomes > Cancer register | 100092 |
| 40006_C340 | Type of cancer: ICD10-C34.0 Main bronchus | Health-related outcomes > Cancer register | 100092 |
| 40006_C341 | Type of cancer: ICD10-C34.1 Upper lobe, bronchus or lung | Health-related outcomes > Cancer register | 100092 |
| 40006_C342 | Type of cancer: ICD10-C34.2 Middle lobe, bronchus or lung | Health-related outcomes > Cancer register | 100092 |
| 40006_C343 | Type of cancer: ICD10-C34.3 Lower lobe, bronchus or lung | Health-related outcomes > Cancer register | 100092 |
| 40006_C349 | Type of cancer: ICD10-C34.9 Bronchus or lung, unspecified | Health-related outcomes > Cancer register | 100092 |
| 40006_C433 | Type of cancer: ICD10-C43.3 Malignant melanoma of other and unspecified parts of face | Health-related outcomes > Cancer register | 100092 |
| 40006_C434 | Type of cancer: ICD10-C43.4 Malignant melanoma of scalp and neck | Health-related outcomes > Cancer register | 100092 |
| 40006_C435 | Type of cancer: ICD10-C43.5 Malignant melanoma of trunk | Health-related outcomes > Cancer register | 100092 |
| 40006_C436 | Type of cancer: ICD10-C43.6 Malignant melanoma of upper limb, including shoulder | Health-related outcomes > Cancer register | 100092 |
| 40006_C437 | Type of cancer: ICD10-C43.7 Malignant melanoma of lower limb, including hip | Health-related outcomes > Cancer register | 100092 |
| 40006_C439 | Type of cancer: ICD10-C43.9 Malignant melanoma of skin, unspecified | Health-related outcomes > Cancer register | 100092 |
| 40006_C440 | Type of cancer: ICD10-C44.0 Skin of lip | Health-related outcomes > Cancer register | 100092 |
| 40006_C441 | Type of cancer: ICD10-C44.1 Skin of eyelid, including canthus | Health-related outcomes > Cancer register | 100092 |
| 40006_C442 | Type of cancer: ICD10-C44.2 Skin of ear and external auricular canal | Health-related outcomes > Cancer register | 100092 |
| 40006_C443 | Type of cancer: ICD10-C44.3 Skin of other and unspecified parts of face | Health-related outcomes > Cancer register | 100092 |
| 40006_C444 | Type of cancer: ICD10-C44.4 Skin of scalp and neck | Health-related outcomes > Cancer register | 100092 |
| 40006_C445 | Type of cancer: ICD10-C44.5 Skin of trunk | Health-related outcomes > Cancer register | 100092 |
| 40006_C446 | Type of cancer: ICD10-C44.6 Skin of upper limb, including shoulder | Health-related outcomes > Cancer register | 100092 |
| 40006_C447 | Type of cancer: ICD10-C44.7 Skin of lower limb, including hip | Health-related outcomes > Cancer register | 100092 |
| 40006_C448 | Type of cancer: ICD10-C44.8 Overlapping lesion of skin | Health-related outcomes > Cancer register | 100092 |
| 40006_C449 | Type of cancer: ICD10-C44.9 Malignant neoplasm of skin, unspecified | Health-related outcomes > Cancer register | 100092 |
| 40006_C450 | Type of cancer: ICD10-C45.0 Mesothelioma of pleura | Health-related outcomes > Cancer register | 100092 |
| 40006_C492 | Type of cancer: ICD10-C49.2 Connective and soft tissue of lower limb, including hip | Health-related outcomes > Cancer register | 100092 |
| 40006_C500 | Type of cancer: ICD10-C50.0 Nipple and areola | Health-related outcomes > Cancer register | 100092 |
| 40006_C501 | Type of cancer: ICD10-C50.1 Central portion of breast | Health-related outcomes > Cancer register | 100092 |
| 40006_C502 | Type of cancer: ICD10-C50.2 Upper-inner quadrant of breast | Health-related outcomes > Cancer register | 100092 |
| 40006_C503 | Type of cancer: ICD10-C50.3 Lower-inner quadrant of breast | Health-related outcomes > Cancer register | 100092 |
| 40006_C504 | Type of cancer: ICD10-C50.4 Upper-outer quadrant of breast | Health-related outcomes > Cancer register | 100092 |
| 40006_C505 | Type of cancer: ICD10-C50.5 Lower-outer quadrant of breast | Health-related outcomes > Cancer register | 100092 |
| 40006_C508 | Type of cancer: ICD10-C50.8 Overlapping lesion of breast | Health-related outcomes > Cancer register | 100092 |
| 40006_C509 | Type of cancer: ICD10-C50.9 Breast, unspecified | Health-related outcomes > Cancer register | 100092 |
| 40006_C519 | Type of cancer: ICD10-C51.9 Vulva, unspecified | Health-related outcomes > Cancer register | 100092 |
| 40006_C539 | Type of cancer: ICD10-C53.9 Cervix uteri, unspecified | Health-related outcomes > Cancer register | 100092 |
| 40006_C541 | Type of cancer: ICD10-C54.1 Endometrium | Health-related outcomes > Cancer register | 100092 |
| 40006_C56 | Type of cancer: ICD10-C56 Malignant neoplasm of ovary | Health-related outcomes > Cancer register | 100092 |
| 40006_C61 | Type of cancer: ICD10-C61 Malignant neoplasm of prostate | Health-related outcomes > Cancer register | 100092 |
| 40006_C621 | Type of cancer: ICD10-C62.1 Descended testis | Health-related outcomes > Cancer register | 100092 |
| 40006_C629 | Type of cancer: ICD10-C62.9 Testis, unspecified | Health-related outcomes > Cancer register | 100092 |
| 40006_C64 | Type of cancer: ICD10-C64 Malignant neoplasm of kidney, except renal pelvis | Health-related outcomes > Cancer register | 100092 |
| 40006_C679 | Type of cancer: ICD10-C67.9 Bladder, unspecified | Health-related outcomes > Cancer register | 100092 |
| 40006_C693 | Type of cancer: ICD10-C69.3 Choroid | Health-related outcomes > Cancer register | 100092 |
| 40006_C711 | Type of cancer: ICD10-C71.1 Frontal lobe | Health-related outcomes > Cancer register | 100092 |
| 40006_C712 | Type of cancer: ICD10-C71.2 Temporal lobe | Health-related outcomes > Cancer register | 100092 |
| 40006_C719 | Type of cancer: ICD10-C71.9 Brain, unspecified | Health-related outcomes > Cancer register | 100092 |
| 40006_C73 | Type of cancer: ICD10-C73 Malignant neoplasm of thyroid gland | Health-related outcomes > Cancer register | 100092 |
| 40006_C80 | Type of cancer: ICD10-C80 Malignant neoplasm without specification of site | Health-related outcomes > Cancer register | 100092 |
| 40006_C811 | Type of cancer: ICD10-C81.1 Nodular sclerosis | Health-related outcomes > Cancer register | 100092 |
| 40006_C819 | Type of cancer: ICD10-C81.9 Hodgkin's disease, unspecified | Health-related outcomes > Cancer register | 100092 |
| 40006_C829 | Type of cancer: ICD10-C82.9 Follicular non-Hodgkin's lymphoma, unspecified | Health-related outcomes > Cancer register | 100092 |
| 40006_C830 | Type of cancer: ICD10-C83.0 Small cell (diffuse) | Health-related outcomes > Cancer register | 100092 |
| 40006_C833 | Type of cancer: ICD10-C83.3 Large cell (diffuse) | Health-related outcomes > Cancer register | 100092 |
| 40006_C838 | Type of cancer: ICD10-C83.8 Other types of diffuse non-Hodgkin's lymphoma | Health-related outcomes > Cancer register | 100092 |
| 40006_C851 | Type of cancer: ICD10-C85.1 B-cell lymphoma, unspecified | Health-related outcomes > Cancer register | 100092 |
| 40006_C857 | Type of cancer: ICD10-C85.7 Other specified types of non-Hodgkin's lymphoma | Health-related outcomes > Cancer register | 100092 |
| 40006_C859 | Type of cancer: ICD10-C85.9 Non-Hodgkin's lymphoma, unspecified type | Health-related outcomes > Cancer register | 100092 |
| 40006_C900 | Type of cancer: ICD10-C90.0 Multiple myeloma | Health-related outcomes > Cancer register | 100092 |
| 40006_C911 | Type of cancer: ICD10-C91.1 Chronic lymphocytic leukaemia | Health-related outcomes > Cancer register | 100092 |
| 40006_C920 | Type of cancer: ICD10-C92.0 Acute myeloid leukaemia | Health-related outcomes > Cancer register | 100092 |
| 40006_C921 | Type of cancer: ICD10-C92.1 Chronic myeloid leukaemia | Health-related outcomes > Cancer register | 100092 |
| 40006_D010 | Type of cancer: ICD10-D01.0 Colon | Health-related outcomes > Cancer register | 100092 |
| 40006_D012 | Type of cancer: ICD10-D01.2 Rectum | Health-related outcomes > Cancer register | 100092 |
| 40006_D033 | Type of cancer: ICD10-D03.3 Melanoma in situ of other and unspecified parts of face | Health-related outcomes > Cancer register | 100092 |
| 40006_D035 | Type of cancer: ICD10-D03.5 Melanoma in situ of trunk | Health-related outcomes > Cancer register | 100092 |
| 40006_D036 | Type of cancer: ICD10-D03.6 Melanoma in situ of upper limb, including shoulder | Health-related outcomes > Cancer register | 100092 |
| 40006_D037 | Type of cancer: ICD10-D03.7 Melanoma in situ of lower limb, including hip | Health-related outcomes > Cancer register | 100092 |
| 40006_D043 | Type of cancer: ICD10-D04.3 Skin of other and unspecified parts of face | Health-related outcomes > Cancer register | 100092 |
| 40006_D044 | Type of cancer: ICD10-D04.4 Skin of scalp and neck | Health-related outcomes > Cancer register | 100092 |
| 40006_D045 | Type of cancer: ICD10-D04.5 Skin of trunk | Health-related outcomes > Cancer register | 100092 |
| 40006_D046 | Type of cancer: ICD10-D04.6 Skin of upper limb, including shoulder | Health-related outcomes > Cancer register | 100092 |
| 40006_D047 | Type of cancer: ICD10-D04.7 Skin of lower limb, including hip | Health-related outcomes > Cancer register | 100092 |
| 40006_D049 | Type of cancer: ICD10-D04.9 Skin, unspecified | Health-related outcomes > Cancer register | 100092 |
| 40006_D050 | Type of cancer: ICD10-D05.0 Lobular carcinoma in situ | Health-related outcomes > Cancer register | 100092 |
| 40006_D051 | Type of cancer: ICD10-D05.1 Intraductal carcinoma in situ | Health-related outcomes > Cancer register | 100092 |
| 40006_D057 | Type of cancer: ICD10-D05.7 Other carcinoma in situ of breast | Health-related outcomes > Cancer register | 100092 |
| 40006_D059 | Type of cancer: ICD10-D05.9 Carcinoma in situ of breast, unspecified | Health-related outcomes > Cancer register | 100092 |
| 40006_D061 | Type of cancer: ICD10-D06.1 Exocervix | Health-related outcomes > Cancer register | 100092 |
| 40006_D069 | Type of cancer: ICD10-D06.9 Cervix, unspecified | Health-related outcomes > Cancer register | 100092 |
| 40006_D071 | Type of cancer: ICD10-D07.1 Vulva | Health-related outcomes > Cancer register | 100092 |
| 40006_D075 | Type of cancer: ICD10-D07.5 Prostate | Health-related outcomes > Cancer register | 100092 |
| 40006_D090 | Type of cancer: ICD10-D09.0 Bladder | Health-related outcomes > Cancer register | 100092 |
| 40006_D320 | Type of cancer: ICD10-D32.0 Cerebral meninges | Health-related outcomes > Cancer register | 100092 |
| 40006_D329 | Type of cancer: ICD10-D32.9 Meninges, unspecified | Health-related outcomes > Cancer register | 100092 |
| 40006_D333 | Type of cancer: ICD10-D33.3 Cranial nerves | Health-related outcomes > Cancer register | 100092 |
| 40006_D352 | Type of cancer: ICD10-D35.2 Pituitary gland | Health-related outcomes > Cancer register | 100092 |
| 40006_D374 | Type of cancer: ICD10-D37.4 Colon | Health-related outcomes > Cancer register | 100092 |
| 40006_D375 | Type of cancer: ICD10-D37.5 Rectum | Health-related outcomes > Cancer register | 100092 |
| 40006_D390 | Type of cancer: ICD10-D39.0 Uterus | Health-related outcomes > Cancer register | 100092 |
| 40006_D414 | Type of cancer: ICD10-D41.4 Bladder | Health-related outcomes > Cancer register | 100092 |
| 40006_D45 | Type of cancer: ICD10-D45 Polycythaemia vera | Health-related outcomes > Cancer register | 100092 |
| 40006_D469 | Type of cancer: ICD10-D46.9 Myelodysplastic syndrome, unspecified | Health-related outcomes > Cancer register | 100092 |
| 40006_D471 | Type of cancer: ICD10-D47.1 Chronic myeloproliferative disease | Health-related outcomes > Cancer register | 100092 |
| 40006_D472 | Type of cancer: ICD10-D47.2 Monoclonal gammopathy | Health-related outcomes > Cancer register | 100092 |
| 40006_D473 | Type of cancer: ICD10-D47.3 Essential (haemorrhagic) thrombocythaemia | Health-related outcomes > Cancer register | 100092 |
| 40013_C435 | Type of cancer: ICD9-C43.5 Malignant melanoma of trunk | Health-related outcomes > Cancer register | 100092 |
| 40013_C436 | Type of cancer: ICD9-C43.6 Malignant melanoma of upper limb, including shoulder | Health-related outcomes > Cancer register | 100092 |
| 40013_C437 | Type of cancer: ICD9-C43.7 Malignant melanoma of lower limb, including hip | Health-related outcomes > Cancer register | 100092 |
| 40013_C441 | Type of cancer: ICD9-C44.1 Skin of eyelid, including canthus | Health-related outcomes > Cancer register | 100092 |
| 40013_C443 | Type of cancer: ICD9-C44.3 Skin of other and unspecified parts of face | Health-related outcomes > Cancer register | 100092 |
| 40013_C445 | Type of cancer: ICD9-C44.5 Skin of trunk | Health-related outcomes > Cancer register | 100092 |
| 40013_C446 | Type of cancer: ICD9-C44.6 Skin of upper limb, including shoulder | Health-related outcomes > Cancer register | 100092 |
| 40013_C447 | Type of cancer: ICD9-C44.7 Skin of lower limb, including hip | Health-related outcomes > Cancer register | 100092 |
| 40013_C504 | Type of cancer: ICD9-C50.4 Upper-outer quadrant of breast | Health-related outcomes > Cancer register | 100092 |
| 40013_C509 | Type of cancer: ICD9-C50.9 Breast, unspecified | Health-related outcomes > Cancer register | 100092 |
| 40013_C539 | Type of cancer: ICD9-C53.9 Cervix uteri, unspecified | Health-related outcomes > Cancer register | 100092 |
| 40013_C549 | Type of cancer: ICD9-C54.9 Corpus uteri, unspecified | Health-related outcomes > Cancer register | 100092 |
| 40013_C56 | Type of cancer: ICD9-C56 Malignant neoplasm of ovary | Health-related outcomes > Cancer register | 100092 |
| 40013_C629 | Type of cancer: ICD9-C62.9 Testis, unspecified | Health-related outcomes > Cancer register | 100092 |
| 40013_C679 | Type of cancer: ICD9-C67.9 Bladder, unspecified | Health-related outcomes > Cancer register | 100092 |
| 40013_C73 | Type of cancer: ICD9-C73 Malignant neoplasm of thyroid gland | Health-related outcomes > Cancer register | 100092 |
| 40013_C819 | Type of cancer: ICD9-C81.9 Hodgkin's disease, unspecified | Health-related outcomes > Cancer register | 100092 |
| 40013_C857 | Type of cancer: ICD9-C85.7 Other specified types of non-Hodgkin's lymphoma | Health-related outcomes > Cancer register | 100092 |
| 40013_D059 | Type of cancer: ICD9-D05.9 Carcinoma in situ of breast, unspecified | Health-related outcomes > Cancer register | 100092 |
| 40013_D069 | Type of cancer: ICD9-D06.9 Cervix, unspecified | Health-related outcomes > Cancer register | 100092 |
| 40013_D092 | Type of cancer: ICD9-D09.2 Eye | Health-related outcomes > Cancer register | 100092 |
| 40013_D390 | Type of cancer: ICD9-D39.0 Uterus | Health-related outcomes > Cancer register | 100092 |
| 41270_C01 | Diagnoses - ICD10-C01 Malignant neoplasm of base of tongue | Health-related outcomes > Hospital inpatient > Summary Diagnoses | 2002 |
| 41270_C021 | Diagnoses - ICD10-C02.1 Border of tongue | Health-related outcomes > Hospital inpatient > Summary Diagnoses | 2002 |
| 41270_C029 | Diagnoses - ICD10-C02.9 Tongue, unspecified | Health-related outcomes > Hospital inpatient > Summary Diagnoses | 2002 |
| 41270_C07 | Diagnoses - ICD10-C07 Malignant neoplasm of parotid gland | Health-related outcomes > Hospital inpatient > Summary Diagnoses | 2002 |
| 41270_C099 | Diagnoses - ICD10-C09.9 Tonsil, unspecified | Health-related outcomes > Hospital inpatient > Summary Diagnoses | 2002 |
| 41270_C154 | Diagnoses - ICD10-C15.4 Middle third of oesophagus | Health-related outcomes > Hospital inpatient > Summary Diagnoses | 2002 |
| 41270_C155 | Diagnoses - ICD10-C15.5 Lower third of oesophagus | Health-related outcomes > Hospital inpatient > Summary Diagnoses | 2002 |
| 41270_C159 | Diagnoses - ICD10-C15.9 Oesophagus, unspecified | Health-related outcomes > Hospital inpatient > Summary Diagnoses | 2002 |
| 41270_C160 | Diagnoses - ICD10-C16.0 Cardia | Health-related outcomes > Hospital inpatient > Summary Diagnoses | 2002 |
| 41270_C169 | Diagnoses - ICD10-C16.9 Stomach, unspecified | Health-related outcomes > Hospital inpatient > Summary Diagnoses | 2002 |
| 41270_C180 | Diagnoses - ICD10-C18.0 Caecum | Health-related outcomes > Hospital inpatient > Summary Diagnoses | 2002 |
| 41270_C181 | Diagnoses - ICD10-C18.1 Appendix | Health-related outcomes > Hospital inpatient > Summary Diagnoses | 2002 |
| 41270_C182 | Diagnoses - ICD10-C18.2 Ascending colon | Health-related outcomes > Hospital inpatient > Summary Diagnoses | 2002 |
| 41270_C183 | Diagnoses - ICD10-C18.3 Hepatic flexure | Health-related outcomes > Hospital inpatient > Summary Diagnoses | 2002 |
| 41270_C184 | Diagnoses - ICD10-C18.4 Transverse colon | Health-related outcomes > Hospital inpatient > Summary Diagnoses | 2002 |
| 41270_C185 | Diagnoses - ICD10-C18.5 Splenic flexure | Health-related outcomes > Hospital inpatient > Summary Diagnoses | 2002 |
| 41270_C186 | Diagnoses - ICD10-C18.6 Descending colon | Health-related outcomes > Hospital inpatient > Summary Diagnoses | 2002 |
| 41270_C187 | Diagnoses - ICD10-C18.7 Sigmoid colon | Health-related outcomes > Hospital inpatient > Summary Diagnoses | 2002 |
| 41270_C189 | Diagnoses - ICD10-C18.9 Colon, unspecified | Health-related outcomes > Hospital inpatient > Summary Diagnoses | 2002 |
| 41270_C19 | Diagnoses - ICD10-C19 Malignant neoplasm of rectosigmoid junction | Health-related outcomes > Hospital inpatient > Summary Diagnoses | 2002 |
| 41270_C20 | Diagnoses - ICD10-C20 Malignant neoplasm of rectum | Health-related outcomes > Hospital inpatient > Summary Diagnoses | 2002 |
| 41270_C210 | Diagnoses - ICD10-C21.0 Anus, unspecified | Health-related outcomes > Hospital inpatient > Summary Diagnoses | 2002 |
| 41270_C211 | Diagnoses - ICD10-C21.1 Anal canal | Health-related outcomes > Hospital inpatient > Summary Diagnoses | 2002 |
| 41270_C220 | Diagnoses - ICD10-C22.0 Liver cell carcinoma | Health-related outcomes > Hospital inpatient > Summary Diagnoses | 2002 |
| 41270_C221 | Diagnoses - ICD10-C22.1 Intrahepatic bile duct carcinoma | Health-related outcomes > Hospital inpatient > Summary Diagnoses | 2002 |
| 41270_C229 | Diagnoses - ICD10-C22.9 Liver, unspecified | Health-related outcomes > Hospital inpatient > Summary Diagnoses | 2002 |
| 41270_C23 | Diagnoses - ICD10-C23 Malignant neoplasm of gallbladder | Health-related outcomes > Hospital inpatient > Summary Diagnoses | 2002 |
| 41270_C250 | Diagnoses - ICD10-C25.0 Head of pancreas | Health-related outcomes > Hospital inpatient > Summary Diagnoses | 2002 |
| 41270_C252 | Diagnoses - ICD10-C25.2 Tail of pancreas | Health-related outcomes > Hospital inpatient > Summary Diagnoses | 2002 |
| 41270_C259 | Diagnoses - ICD10-C25.9 Pancreas, unspecified | Health-related outcomes > Hospital inpatient > Summary Diagnoses | 2002 |
| 41270_C260 | Diagnoses - ICD10-C26.0 Intestinal tract, part unspecified | Health-related outcomes > Hospital inpatient > Summary Diagnoses | 2002 |
| 41270_C320 | Diagnoses - ICD10-C32.0 Glottis | Health-related outcomes > Hospital inpatient > Summary Diagnoses | 2002 |
| 41270_C329 | Diagnoses - ICD10-C32.9 Larynx, unspecified | Health-related outcomes > Hospital inpatient > Summary Diagnoses | 2002 |
| 41270_C340 | Diagnoses - ICD10-C34.0 Main bronchus | Health-related outcomes > Hospital inpatient > Summary Diagnoses | 2002 |
| 41270_C341 | Diagnoses - ICD10-C34.1 Upper lobe, bronchus or lung | Health-related outcomes > Hospital inpatient > Summary Diagnoses | 2002 |
| 41270_C342 | Diagnoses - ICD10-C34.2 Middle lobe, bronchus or lung | Health-related outcomes > Hospital inpatient > Summary Diagnoses | 2002 |
| 41270_C343 | Diagnoses - ICD10-C34.3 Lower lobe, bronchus or lung | Health-related outcomes > Hospital inpatient > Summary Diagnoses | 2002 |
| 41270_C349 | Diagnoses - ICD10-C34.9 Bronchus or lung, unspecified | Health-related outcomes > Hospital inpatient > Summary Diagnoses | 2002 |
| 41270_C433 | Diagnoses - ICD10-C43.3 Malignant melanoma of other and unspecified parts of face | Health-related outcomes > Hospital inpatient > Summary Diagnoses | 2002 |
| 41270_C434 | Diagnoses - ICD10-C43.4 Malignant melanoma of scalp and neck | Health-related outcomes > Hospital inpatient > Summary Diagnoses | 2002 |
| 41270_C435 | Diagnoses - ICD10-C43.5 Malignant melanoma of trunk | Health-related outcomes > Hospital inpatient > Summary Diagnoses | 2002 |
| 41270_C436 | Diagnoses - ICD10-C43.6 Malignant melanoma of upper limb, including shoulder | Health-related outcomes > Hospital inpatient > Summary Diagnoses | 2002 |
| 41270_C437 | Diagnoses - ICD10-C43.7 Malignant melanoma of lower limb, including hip | Health-related outcomes > Hospital inpatient > Summary Diagnoses | 2002 |
| 41270_C439 | Diagnoses - ICD10-C43.9 Malignant melanoma of skin, unspecified | Health-related outcomes > Hospital inpatient > Summary Diagnoses | 2002 |
| 41270_C440 | Diagnoses - ICD10-C44.0 Skin of lip | Health-related outcomes > Hospital inpatient > Summary Diagnoses | 2002 |
| 41270_C441 | Diagnoses - ICD10-C44.1 Skin of eyelid, including canthus | Health-related outcomes > Hospital inpatient > Summary Diagnoses | 2002 |
| 41270_C442 | Diagnoses - ICD10-C44.2 Skin of ear and external auricular canal | Health-related outcomes > Hospital inpatient > Summary Diagnoses | 2002 |
| 41270_C443 | Diagnoses - ICD10-C44.3 Skin of other and unspecified parts of face | Health-related outcomes > Hospital inpatient > Summary Diagnoses | 2002 |
| 41270_C444 | Diagnoses - ICD10-C44.4 Skin of scalp and neck | Health-related outcomes > Hospital inpatient > Summary Diagnoses | 2002 |
| 41270_C445 | Diagnoses - ICD10-C44.5 Skin of trunk | Health-related outcomes > Hospital inpatient > Summary Diagnoses | 2002 |
| 41270_C446 | Diagnoses - ICD10-C44.6 Skin of upper limb, including shoulder | Health-related outcomes > Hospital inpatient > Summary Diagnoses | 2002 |
| 41270_C447 | Diagnoses - ICD10-C44.7 Skin of lower limb, including hip | Health-related outcomes > Hospital inpatient > Summary Diagnoses | 2002 |
| 41270_C449 | Diagnoses - ICD10-C44.9 Malignant neoplasm of skin, unspecified | Health-related outcomes > Hospital inpatient > Summary Diagnoses | 2002 |
| 41270_C450 | Diagnoses - ICD10-C45.0 Mesothelioma of pleura | Health-related outcomes > Hospital inpatient > Summary Diagnoses | 2002 |
| 41270_C459 | Diagnoses - ICD10-C45.9 Mesothelioma, unspecified | Health-related outcomes > Hospital inpatient > Summary Diagnoses | 2002 |
| 41270_C482 | Diagnoses - ICD10-C48.2 Peritoneum, unspecified | Health-related outcomes > Hospital inpatient > Summary Diagnoses | 2002 |
| 41270_C492 | Diagnoses - ICD10-C49.2 Connective and soft tissue of lower limb, including hip | Health-related outcomes > Hospital inpatient > Summary Diagnoses | 2002 |
| 41270_C500 | Diagnoses - ICD10-C50.0 Nipple and areola | Health-related outcomes > Hospital inpatient > Summary Diagnoses | 2002 |
| 41270_C501 | Diagnoses - ICD10-C50.1 Central portion of breast | Health-related outcomes > Hospital inpatient > Summary Diagnoses | 2002 |
| 41270_C502 | Diagnoses - ICD10-C50.2 Upper-inner quadrant of breast | Health-related outcomes > Hospital inpatient > Summary Diagnoses | 2002 |
| 41270_C503 | Diagnoses - ICD10-C50.3 Lower-inner quadrant of breast | Health-related outcomes > Hospital inpatient > Summary Diagnoses | 2002 |
| 41270_C504 | Diagnoses - ICD10-C50.4 Upper-outer quadrant of breast | Health-related outcomes > Hospital inpatient > Summary Diagnoses | 2002 |
| 41270_C505 | Diagnoses - ICD10-C50.5 Lower-outer quadrant of breast | Health-related outcomes > Hospital inpatient > Summary Diagnoses | 2002 |
| 41270_C508 | Diagnoses - ICD10-C50.8 Overlapping lesion of breast | Health-related outcomes > Hospital inpatient > Summary Diagnoses | 2002 |
| 41270_C509 | Diagnoses - ICD10-C50.9 Breast, unspecified | Health-related outcomes > Hospital inpatient > Summary Diagnoses | 2002 |
| 41270_C519 | Diagnoses - ICD10-C51.9 Vulva, unspecified | Health-related outcomes > Hospital inpatient > Summary Diagnoses | 2002 |
| 41270_C539 | Diagnoses - ICD10-C53.9 Cervix uteri, unspecified | Health-related outcomes > Hospital inpatient > Summary Diagnoses | 2002 |
| 41270_C541 | Diagnoses - ICD10-C54.1 Endometrium | Health-related outcomes > Hospital inpatient > Summary Diagnoses | 2002 |
| 41270_C55 | Diagnoses - ICD10-C55 Malignant neoplasm of uterus, part unspecified | Health-related outcomes > Hospital inpatient > Summary Diagnoses | 2002 |
| 41270_C56 | Diagnoses - ICD10-C56 Malignant neoplasm of ovary | Health-related outcomes > Hospital inpatient > Summary Diagnoses | 2002 |
| 41270_C61 | Diagnoses - ICD10-C61 Malignant neoplasm of prostate | Health-related outcomes > Hospital inpatient > Summary Diagnoses | 2002 |
| 41270_C629 | Diagnoses - ICD10-C62.9 Testis, unspecified | Health-related outcomes > Hospital inpatient > Summary Diagnoses | 2002 |
| 41270_C64 | Diagnoses - ICD10-C64 Malignant neoplasm of kidney, except renal pelvis | Health-related outcomes > Hospital inpatient > Summary Diagnoses | 2002 |
| 41270_C65 | Diagnoses - ICD10-C65 Malignant neoplasm of renal pelvis | Health-related outcomes > Hospital inpatient > Summary Diagnoses | 2002 |
| 41270_C66 | Diagnoses - ICD10-C66 Malignant neoplasm of ureter | Health-related outcomes > Hospital inpatient > Summary Diagnoses | 2002 |
| 41270_C671 | Diagnoses - ICD10-C67.1 Dome of bladder | Health-related outcomes > Hospital inpatient > Summary Diagnoses | 2002 |
| 41270_C672 | Diagnoses - ICD10-C67.2 Lateral wall of bladder | Health-related outcomes > Hospital inpatient > Summary Diagnoses | 2002 |
| 41270_C674 | Diagnoses - ICD10-C67.4 Posterior wall of bladder | Health-related outcomes > Hospital inpatient > Summary Diagnoses | 2002 |
| 41270_C675 | Diagnoses - ICD10-C67.5 Bladder neck | Health-related outcomes > Hospital inpatient > Summary Diagnoses | 2002 |
| 41270_C676 | Diagnoses - ICD10-C67.6 Ureteric orifice | Health-related outcomes > Hospital inpatient > Summary Diagnoses | 2002 |
| 41270_C679 | Diagnoses - ICD10-C67.9 Bladder, unspecified | Health-related outcomes > Hospital inpatient > Summary Diagnoses | 2002 |
| 41270_C693 | Diagnoses - ICD10-C69.3 Choroid | Health-related outcomes > Hospital inpatient > Summary Diagnoses | 2002 |
| 41270_C711 | Diagnoses - ICD10-C71.1 Frontal lobe | Health-related outcomes > Hospital inpatient > Summary Diagnoses | 2002 |
| 41270_C712 | Diagnoses - ICD10-C71.2 Temporal lobe | Health-related outcomes > Hospital inpatient > Summary Diagnoses | 2002 |
| 41270_C713 | Diagnoses - ICD10-C71.3 Parietal lobe | Health-related outcomes > Hospital inpatient > Summary Diagnoses | 2002 |
| 41270_C719 | Diagnoses - ICD10-C71.9 Brain, unspecified | Health-related outcomes > Hospital inpatient > Summary Diagnoses | 2002 |
| 41270_C73 | Diagnoses - ICD10-C73 Malignant neoplasm of thyroid gland | Health-related outcomes > Hospital inpatient > Summary Diagnoses | 2002 |
| 41270_C770 | Diagnoses - ICD10-C77.0 Lymph nodes of head, face and neck | Health-related outcomes > Hospital inpatient > Summary Diagnoses | 2002 |
| 41270_C771 | Diagnoses - ICD10-C77.1 Intrathoracic lymph nodes | Health-related outcomes > Hospital inpatient > Summary Diagnoses | 2002 |
| 41270_C772 | Diagnoses - ICD10-C77.2 Intra-abdominal lymph nodes | Health-related outcomes > Hospital inpatient > Summary Diagnoses | 2002 |
| 41270_C773 | Diagnoses - ICD10-C77.3 Axillary and upper limb lymph nodes | Health-related outcomes > Hospital inpatient > Summary Diagnoses | 2002 |
| 41270_C774 | Diagnoses - ICD10-C77.4 Inguinal and lower limb lymph nodes | Health-related outcomes > Hospital inpatient > Summary Diagnoses | 2002 |
| 41270_C775 | Diagnoses - ICD10-C77.5 Intrapelvic lymph nodes | Health-related outcomes > Hospital inpatient > Summary Diagnoses | 2002 |
| 41270_C778 | Diagnoses - ICD10-C77.8 Lymph nodes of multiple regions | Health-related outcomes > Hospital inpatient > Summary Diagnoses | 2002 |
| 41270_C779 | Diagnoses - ICD10-C77.9 Lymph node, unspecified | Health-related outcomes > Hospital inpatient > Summary Diagnoses | 2002 |
| 41270_C780 | Diagnoses - ICD10-C78.0 Secondary malignant neoplasm of lung | Health-related outcomes > Hospital inpatient > Summary Diagnoses | 2002 |
| 41270_C781 | Diagnoses - ICD10-C78.1 Secondary malignant neoplasm of mediastinum | Health-related outcomes > Hospital inpatient > Summary Diagnoses | 2002 |
| 41270_C782 | Diagnoses - ICD10-C78.2 Secondary malignant neoplasm of pleura | Health-related outcomes > Hospital inpatient > Summary Diagnoses | 2002 |
| 41270_C784 | Diagnoses - ICD10-C78.4 Secondary malignant neoplasm of small intestine | Health-related outcomes > Hospital inpatient > Summary Diagnoses | 2002 |
| 41270_C785 | Diagnoses - ICD10-C78.5 Secondary malignant neoplasm of large intestine and rectum | Health-related outcomes > Hospital inpatient > Summary Diagnoses | 2002 |
| 41270_C786 | Diagnoses - ICD10-C78.6 Secondary malignant neoplasm of retroperitoneum and peritoneum | Health-related outcomes > Hospital inpatient > Summary Diagnoses | 2002 |
| 41270_C787 | Diagnoses - ICD10-C78.7 Secondary malignant neoplasm of liver | Health-related outcomes > Hospital inpatient > Summary Diagnoses | 2002 |
| 41270_C788 | Diagnoses - ICD10-C78.8 Secondary malignant neoplasm of other and unspecified digestive organs | Health-related outcomes > Hospital inpatient > Summary Diagnoses | 2002 |
| 41270_C790 | Diagnoses - ICD10-C79.0 Secondary malignant neoplasm of kidney and renal pelvis | Health-related outcomes > Hospital inpatient > Summary Diagnoses | 2002 |
| 41270_C791 | Diagnoses - ICD10-C79.1 Secondary malignant neoplasm of bladder and other and unspecified urinary organs | Health-related outcomes > Hospital inpatient > Summary Diagnoses | 2002 |
| 41270_C792 | Diagnoses - ICD10-C79.2 Secondary malignant neoplasm of skin | Health-related outcomes > Hospital inpatient > Summary Diagnoses | 2002 |
| 41270_C793 | Diagnoses - ICD10-C79.3 Secondary malignant neoplasm of brain and cerebral meninges | Health-related outcomes > Hospital inpatient > Summary Diagnoses | 2002 |
| 41270_C795 | Diagnoses - ICD10-C79.5 Secondary malignant neoplasm of bone and bone marrow | Health-related outcomes > Hospital inpatient > Summary Diagnoses | 2002 |
| 41270_C796 | Diagnoses - ICD10-C79.6 Secondary malignant neoplasm of ovary | Health-related outcomes > Hospital inpatient > Summary Diagnoses | 2002 |
| 41270_C797 | Diagnoses - ICD10-C79.7 Secondary malignant neoplasm of adrenal gland | Health-related outcomes > Hospital inpatient > Summary Diagnoses | 2002 |
| 41270_C798 | Diagnoses - ICD10-C79.8 Secondary malignant neoplasm of other specified sites | Health-related outcomes > Hospital inpatient > Summary Diagnoses | 2002 |
| 41270_C799 | Diagnoses - ICD10-C79.9 Secondary malignant neoplasm, unspecified site | Health-related outcomes > Hospital inpatient > Summary Diagnoses | 2002 |
| 41270_C80 | Diagnoses - ICD10-C80 Malignant neoplasm without specification of site | Health-related outcomes > Hospital inpatient > Summary Diagnoses | 2002 |
| 41270_C800 | Diagnoses - ICD10-C80.0 Malignant neoplasm, primary site unknown, so stated | Health-related outcomes > Hospital inpatient > Summary Diagnoses | 2002 |
| 41270_C809 | Diagnoses - ICD10-C80.9 Malignant neoplasm, unspecified | Health-related outcomes > Hospital inpatient > Summary Diagnoses | 2002 |
| 41270_C819 | Diagnoses - ICD10-C81.9 Hodgkin's disease, unspecified | Health-related outcomes > Hospital inpatient > Summary Diagnoses | 2002 |
| 41270_C829 | Diagnoses - ICD10-C82.9 Follicular non-Hodgkin's lymphoma, unspecified | Health-related outcomes > Hospital inpatient > Summary Diagnoses | 2002 |
| 41270_C830 | Diagnoses - ICD10-C83.0 Small cell (diffuse) | Health-related outcomes > Hospital inpatient > Summary Diagnoses | 2002 |
| 41270_C833 | Diagnoses - ICD10-C83.3 Large cell (diffuse) | Health-related outcomes > Hospital inpatient > Summary Diagnoses | 2002 |
| 41270_C838 | Diagnoses - ICD10-C83.8 Other types of diffuse non-Hodgkin's lymphoma | Health-related outcomes > Hospital inpatient > Summary Diagnoses | 2002 |
| 41270_C851 | Diagnoses - ICD10-C85.1 B-cell lymphoma, unspecified | Health-related outcomes > Hospital inpatient > Summary Diagnoses | 2002 |
| 41270_C857 | Diagnoses - ICD10-C85.7 Other specified types of non-Hodgkin's lymphoma | Health-related outcomes > Hospital inpatient > Summary Diagnoses | 2002 |
| 41270_C859 | Diagnoses - ICD10-C85.9 Non-Hodgkin's lymphoma, unspecified type | Health-related outcomes > Hospital inpatient > Summary Diagnoses | 2002 |
| 41270_C880 | Diagnoses - ICD10-C88.0 Waldenstrom's macroglobulinaemia | Health-related outcomes > Hospital inpatient > Summary Diagnoses | 2002 |
| 41270_C900 | Diagnoses - ICD10-C90.0 Multiple myeloma | Health-related outcomes > Hospital inpatient > Summary Diagnoses | 2002 |
| 41270_C911 | Diagnoses - ICD10-C91.1 Chronic lymphocytic leukaemia | Health-related outcomes > Hospital inpatient > Summary Diagnoses | 2002 |
| 41270_C920 | Diagnoses - ICD10-C92.0 Acute myeloid leukaemia | Health-related outcomes > Hospital inpatient > Summary Diagnoses | 2002 |
| 41270_C921 | Diagnoses - ICD10-C92.1 Chronic myeloid leukaemia | Health-related outcomes > Hospital inpatient > Summary Diagnoses | 2002 |
| 41270_C97 | Diagnoses - ICD10-C97 Malignant neoplasms of independent (primary) multiple sites | Health-related outcomes > Hospital inpatient > Summary Diagnoses | 2002 |
| 41270_D033 | Diagnoses - ICD10-D03.3 Melanoma in situ of other and unspecified parts of face | Health-related outcomes > Hospital inpatient > Summary Diagnoses | 2002 |
| 41270_D035 | Diagnoses - ICD10-D03.5 Melanoma in situ of trunk | Health-related outcomes > Hospital inpatient > Summary Diagnoses | 2002 |
| 41270_D036 | Diagnoses - ICD10-D03.6 Melanoma in situ of upper limb, including shoulder | Health-related outcomes > Hospital inpatient > Summary Diagnoses | 2002 |
| 41270_D037 | Diagnoses - ICD10-D03.7 Melanoma in situ of lower limb, including hip | Health-related outcomes > Hospital inpatient > Summary Diagnoses | 2002 |
| 41270_D043 | Diagnoses - ICD10-D04.3 Skin of other and unspecified parts of face | Health-related outcomes > Hospital inpatient > Summary Diagnoses | 2002 |
| 41270_D044 | Diagnoses - ICD10-D04.4 Skin of scalp and neck | Health-related outcomes > Hospital inpatient > Summary Diagnoses | 2002 |
| 41270_D045 | Diagnoses - ICD10-D04.5 Skin of trunk | Health-related outcomes > Hospital inpatient > Summary Diagnoses | 2002 |
| 41270_D046 | Diagnoses - ICD10-D04.6 Skin of upper limb, including shoulder | Health-related outcomes > Hospital inpatient > Summary Diagnoses | 2002 |
| 41270_D047 | Diagnoses - ICD10-D04.7 Skin of lower limb, including hip | Health-related outcomes > Hospital inpatient > Summary Diagnoses | 2002 |
| 41270_D050 | Diagnoses - ICD10-D05.0 Lobular carcinoma in situ | Health-related outcomes > Hospital inpatient > Summary Diagnoses | 2002 |
| 41270_D051 | Diagnoses - ICD10-D05.1 Intraductal carcinoma in situ | Health-related outcomes > Hospital inpatient > Summary Diagnoses | 2002 |
| 41270_D059 | Diagnoses - ICD10-D05.9 Carcinoma in situ of breast, unspecified | Health-related outcomes > Hospital inpatient > Summary Diagnoses | 2002 |
| 41270_D069 | Diagnoses - ICD10-D06.9 Cervix, unspecified | Health-related outcomes > Hospital inpatient > Summary Diagnoses | 2002 |
| 41270_D071 | Diagnoses - ICD10-D07.1 Vulva | Health-related outcomes > Hospital inpatient > Summary Diagnoses | 2002 |
| 41270_D075 | Diagnoses - ICD10-D07.5 Prostate | Health-related outcomes > Hospital inpatient > Summary Diagnoses | 2002 |
| 41270_D090 | Diagnoses - ICD10-D09.0 Bladder | Health-related outcomes > Hospital inpatient > Summary Diagnoses | 2002 |
| 41270_D101 | Diagnoses - ICD10-D10.1 Tongue | Health-related outcomes > Hospital inpatient > Summary Diagnoses | 2002 |
| 41270_D103 | Diagnoses - ICD10-D10.3 Other and unspecified parts of mouth | Health-related outcomes > Hospital inpatient > Summary Diagnoses | 2002 |
| 41270_D110 | Diagnoses - ICD10-D11.0 Parotid gland | Health-related outcomes > Hospital inpatient > Summary Diagnoses | 2002 |
| 41270_D120 | Diagnoses - ICD10-D12.0 Caecum | Health-related outcomes > Hospital inpatient > Summary Diagnoses | 2002 |
| 41270_D122 | Diagnoses - ICD10-D12.2 Ascending colon | Health-related outcomes > Hospital inpatient > Summary Diagnoses | 2002 |
| 41270_D123 | Diagnoses - ICD10-D12.3 Transverse colon | Health-related outcomes > Hospital inpatient > Summary Diagnoses | 2002 |
| 41270_D124 | Diagnoses - ICD10-D12.4 Descending colon | Health-related outcomes > Hospital inpatient > Summary Diagnoses | 2002 |
| 41270_D125 | Diagnoses - ICD10-D12.5 Sigmoid colon | Health-related outcomes > Hospital inpatient > Summary Diagnoses | 2002 |
| 41270_D126 | Diagnoses - ICD10-D12.6 Colon, unspecified | Health-related outcomes > Hospital inpatient > Summary Diagnoses | 2002 |
| 41270_D127 | Diagnoses - ICD10-D12.7 Rectosigmoid junction | Health-related outcomes > Hospital inpatient > Summary Diagnoses | 2002 |
| 41270_D128 | Diagnoses - ICD10-D12.8 Rectum | Health-related outcomes > Hospital inpatient > Summary Diagnoses | 2002 |
| 41270_D129 | Diagnoses - ICD10-D12.9 Anus and anal canal | Health-related outcomes > Hospital inpatient > Summary Diagnoses | 2002 |
| 41270_D130 | Diagnoses - ICD10-D13.0 Oesophagus | Health-related outcomes > Hospital inpatient > Summary Diagnoses | 2002 |
| 41270_D131 | Diagnoses - ICD10-D13.1 Stomach | Health-related outcomes > Hospital inpatient > Summary Diagnoses | 2002 |
| 41270_D132 | Diagnoses - ICD10-D13.2 Duodenum | Health-related outcomes > Hospital inpatient > Summary Diagnoses | 2002 |
| 41270_D140 | Diagnoses - ICD10-D14.0 Middle ear, nasal cavity and accessory sinuses | Health-related outcomes > Hospital inpatient > Summary Diagnoses | 2002 |
| 41270_D164 | Diagnoses - ICD10-D16.4 Bones of skull and face | Health-related outcomes > Hospital inpatient > Summary Diagnoses | 2002 |
| 41270_D170 | Diagnoses - ICD10-D17.0 Benign lipomatous neoplasm of skin and subcutaneous tissue of head, face and neck | Health-related outcomes > Hospital inpatient > Summary Diagnoses | 2002 |
| 41270_D171 | Diagnoses - ICD10-D17.1 Benign lipomatous neoplasm of skin and subcutaneous tissue of trunk | Health-related outcomes > Hospital inpatient > Summary Diagnoses | 2002 |
| 41270_D172 | Diagnoses - ICD10-D17.2 Benign lipomatous neoplasm of skin and subcutaneous tissue of limbs | Health-related outcomes > Hospital inpatient > Summary Diagnoses | 2002 |
| 41270_D173 | Diagnoses - ICD10-D17.3 Benign lipomatous neoplasm of skin and subcutaneous tissue of other and unspecified sites | Health-related outcomes > Hospital inpatient > Summary Diagnoses | 2002 |
| 41270_D175 | Diagnoses - ICD10-D17.5 Benign lipomatous neoplasm of intra-abdominal organs | Health-related outcomes > Hospital inpatient > Summary Diagnoses | 2002 |
| 41270_D176 | Diagnoses - ICD10-D17.6 Benign lipomatous neoplasm of spermatic cord | Health-related outcomes > Hospital inpatient > Summary Diagnoses | 2002 |
| 41270_D177 | Diagnoses - ICD10-D17.7 Benign lipomatous neoplasm of other sites | Health-related outcomes > Hospital inpatient > Summary Diagnoses | 2002 |
| 41270_D179 | Diagnoses - ICD10-D17.9 Benign lipomatous neoplasm, unspecified | Health-related outcomes > Hospital inpatient > Summary Diagnoses | 2002 |
| 41270_D180 | Diagnoses - ICD10-D18.0 Haemangioma, any site | Health-related outcomes > Hospital inpatient > Summary Diagnoses | 2002 |
| 41270_D210 | Diagnoses - ICD10-D21.0 Connective and other soft tissue of head, face and neck | Health-related outcomes > Hospital inpatient > Summary Diagnoses | 2002 |
| 41270_D211 | Diagnoses - ICD10-D21.1 Connective and other soft tissue of upper limb, including shoulder | Health-related outcomes > Hospital inpatient > Summary Diagnoses | 2002 |
| 41270_D212 | Diagnoses - ICD10-D21.2 Connective and other soft tissue of lower limb, including hip | Health-related outcomes > Hospital inpatient > Summary Diagnoses | 2002 |
| 41270_D220 | Diagnoses - ICD10-D22.0 Melanocytic naevi of lip | Health-related outcomes > Hospital inpatient > Summary Diagnoses | 2002 |
| 41270_D221 | Diagnoses - ICD10-D22.1 Melanocytic naevi of eyelid, including canthus | Health-related outcomes > Hospital inpatient > Summary Diagnoses | 2002 |
| 41270_D222 | Diagnoses - ICD10-D22.2 Melanocytic naevi of ear and external auricular canal | Health-related outcomes > Hospital inpatient > Summary Diagnoses | 2002 |
| 41270_D223 | Diagnoses - ICD10-D22.3 Melanocytic naevi of other and unspecified parts of face | Health-related outcomes > Hospital inpatient > Summary Diagnoses | 2002 |
| 41270_D224 | Diagnoses - ICD10-D22.4 Melanocytic naevi of scalp and neck | Health-related outcomes > Hospital inpatient > Summary Diagnoses | 2002 |
| 41270_D225 | Diagnoses - ICD10-D22.5 Melanocytic naevi of trunk | Health-related outcomes > Hospital inpatient > Summary Diagnoses | 2002 |
| 41270_D226 | Diagnoses - ICD10-D22.6 Melanocytic naevi of upper limb, including shoulder | Health-related outcomes > Hospital inpatient > Summary Diagnoses | 2002 |
| 41270_D227 | Diagnoses - ICD10-D22.7 Melanocytic naevi of lower limb, including hip | Health-related outcomes > Hospital inpatient > Summary Diagnoses | 2002 |
| 41270_D229 | Diagnoses - ICD10-D22.9 Melanocytic naevi, unspecified | Health-related outcomes > Hospital inpatient > Summary Diagnoses | 2002 |
| 41270_D230 | Diagnoses - ICD10-D23.0 Skin of lip | Health-related outcomes > Hospital inpatient > Summary Diagnoses | 2002 |
| 41270_D231 | Diagnoses - ICD10-D23.1 Skin of eyelid, including canthus | Health-related outcomes > Hospital inpatient > Summary Diagnoses | 2002 |
| 41270_D232 | Diagnoses - ICD10-D23.2 Skin of ear and external auricular canal | Health-related outcomes > Hospital inpatient > Summary Diagnoses | 2002 |
| 41270_D233 | Diagnoses - ICD10-D23.3 Skin of other and unspecified parts of face | Health-related outcomes > Hospital inpatient > Summary Diagnoses | 2002 |
| 41270_D234 | Diagnoses - ICD10-D23.4 Skin of scalp and neck | Health-related outcomes > Hospital inpatient > Summary Diagnoses | 2002 |
| 41270_D235 | Diagnoses - ICD10-D23.5 Skin of trunk | Health-related outcomes > Hospital inpatient > Summary Diagnoses | 2002 |
| 41270_D236 | Diagnoses - ICD10-D23.6 Skin of upper limb, including shoulder | Health-related outcomes > Hospital inpatient > Summary Diagnoses | 2002 |
| 41270_D237 | Diagnoses - ICD10-D23.7 Skin of lower limb, including hip | Health-related outcomes > Hospital inpatient > Summary Diagnoses | 2002 |
| 41270_D239 | Diagnoses - ICD10-D23.9 Skin, unspecified | Health-related outcomes > Hospital inpatient > Summary Diagnoses | 2002 |
| 41270_D24 | Diagnoses - ICD10-D24 Benign neoplasm of breast | Health-related outcomes > Hospital inpatient > Summary Diagnoses | 2002 |
| 41270_D250 | Diagnoses - ICD10-D25.0 Submucous leiomyoma of uterus | Health-related outcomes > Hospital inpatient > Summary Diagnoses | 2002 |
| 41270_D251 | Diagnoses - ICD10-D25.1 Intramural leiomyoma of uterus | Health-related outcomes > Hospital inpatient > Summary Diagnoses | 2002 |
| 41270_D252 | Diagnoses - ICD10-D25.2 Subserosal leiomyoma of uterus | Health-related outcomes > Hospital inpatient > Summary Diagnoses | 2002 |
| 41270_D259 | Diagnoses - ICD10-D25.9 Leiomyoma of uterus, unspecified | Health-related outcomes > Hospital inpatient > Summary Diagnoses | 2002 |
| 41270_D260 | Diagnoses - ICD10-D26.0 Cervix uteri | Health-related outcomes > Hospital inpatient > Summary Diagnoses | 2002 |
| 41270_D261 | Diagnoses - ICD10-D26.1 Corpus uteri | Health-related outcomes > Hospital inpatient > Summary Diagnoses | 2002 |
| 41270_D269 | Diagnoses - ICD10-D26.9 Uterus, unspecified | Health-related outcomes > Hospital inpatient > Summary Diagnoses | 2002 |
| 41270_D27 | Diagnoses - ICD10-D27 Benign neoplasm of ovary | Health-related outcomes > Hospital inpatient > Summary Diagnoses | 2002 |
| 41270_D280 | Diagnoses - ICD10-D28.0 Vulva | Health-related outcomes > Hospital inpatient > Summary Diagnoses | 2002 |
| 41270_D291 | Diagnoses - ICD10-D29.1 Prostate | Health-related outcomes > Hospital inpatient > Summary Diagnoses | 2002 |
| 41270_D300 | Diagnoses - ICD10-D30.0 Kidney | Health-related outcomes > Hospital inpatient > Summary Diagnoses | 2002 |
| 41270_D303 | Diagnoses - ICD10-D30.3 Bladder | Health-related outcomes > Hospital inpatient > Summary Diagnoses | 2002 |
| 41270_D320 | Diagnoses - ICD10-D32.0 Cerebral meninges | Health-related outcomes > Hospital inpatient > Summary Diagnoses | 2002 |
| 41270_D329 | Diagnoses - ICD10-D32.9 Meninges, unspecified | Health-related outcomes > Hospital inpatient > Summary Diagnoses | 2002 |
| 41270_D333 | Diagnoses - ICD10-D33.3 Cranial nerves | Health-related outcomes > Hospital inpatient > Summary Diagnoses | 2002 |
| 41270_D34 | Diagnoses - ICD10-D34 Benign neoplasm of thyroid gland | Health-related outcomes > Hospital inpatient > Summary Diagnoses | 2002 |
| 41270_D350 | Diagnoses - ICD10-D35.0 Adrenal gland | Health-related outcomes > Hospital inpatient > Summary Diagnoses | 2002 |
| 41270_D351 | Diagnoses - ICD10-D35.1 Parathyroid gland | Health-related outcomes > Hospital inpatient > Summary Diagnoses | 2002 |
| 41270_D352 | Diagnoses - ICD10-D35.2 Pituitary gland | Health-related outcomes > Hospital inpatient > Summary Diagnoses | 2002 |
| 41270_D361 | Diagnoses - ICD10-D36.1 Peripheral nerves and autonomic nervous system | Health-related outcomes > Hospital inpatient > Summary Diagnoses | 2002 |
| 41270_D367 | Diagnoses - ICD10-D36.7 Other specified sites | Health-related outcomes > Hospital inpatient > Summary Diagnoses | 2002 |
| 41270_D370 | Diagnoses - ICD10-D37.0 Lip, oral cavity and pharynx | Health-related outcomes > Hospital inpatient > Summary Diagnoses | 2002 |
| 41270_D371 | Diagnoses - ICD10-D37.1 Stomach | Health-related outcomes > Hospital inpatient > Summary Diagnoses | 2002 |
| 41270_D374 | Diagnoses - ICD10-D37.4 Colon | Health-related outcomes > Hospital inpatient > Summary Diagnoses | 2002 |
| 41270_D375 | Diagnoses - ICD10-D37.5 Rectum | Health-related outcomes > Hospital inpatient > Summary Diagnoses | 2002 |
| 41270_D377 | Diagnoses - ICD10-D37.7 Other digestive organs | Health-related outcomes > Hospital inpatient > Summary Diagnoses | 2002 |
| 41270_D381 | Diagnoses - ICD10-D38.1 Trachea, bronchus and lung | Health-related outcomes > Hospital inpatient > Summary Diagnoses | 2002 |
| 41270_D391 | Diagnoses - ICD10-D39.1 Ovary | Health-related outcomes > Hospital inpatient > Summary Diagnoses | 2002 |
| 41270_D410 | Diagnoses - ICD10-D41.0 Kidney | Health-related outcomes > Hospital inpatient > Summary Diagnoses | 2002 |
| 41270_D414 | Diagnoses - ICD10-D41.4 Bladder | Health-related outcomes > Hospital inpatient > Summary Diagnoses | 2002 |
| 41270_D432 | Diagnoses - ICD10-D43.2 Brain, unspecified | Health-related outcomes > Hospital inpatient > Summary Diagnoses | 2002 |
| 41270_D45 | Diagnoses - ICD10-D45 Polycythaemia vera | Health-related outcomes > Hospital inpatient > Summary Diagnoses | 2002 |
| 41270_D469 | Diagnoses - ICD10-D46.9 Myelodysplastic syndrome, unspecified | Health-related outcomes > Hospital inpatient > Summary Diagnoses | 2002 |
| 41270_D471 | Diagnoses - ICD10-D47.1 Chronic myeloproliferative disease | Health-related outcomes > Hospital inpatient > Summary Diagnoses | 2002 |
| 41270_D472 | Diagnoses - ICD10-D47.2 Monoclonal gammopathy | Health-related outcomes > Hospital inpatient > Summary Diagnoses | 2002 |
| 41270_D473 | Diagnoses - ICD10-D47.3 Essential (haemorrhagic) thrombocythaemia | Health-related outcomes > Hospital inpatient > Summary Diagnoses | 2002 |
| 41270_D474 | Diagnoses - ICD10-D47.4 Osteomyelofibrosis | Health-related outcomes > Hospital inpatient > Summary Diagnoses | 2002 |
| 41270_D480 | Diagnoses - ICD10-D48.0 Bone and articular cartilage | Health-related outcomes > Hospital inpatient > Summary Diagnoses | 2002 |
| 41270_D481 | Diagnoses - ICD10-D48.1 Connective and other soft tissue | Health-related outcomes > Hospital inpatient > Summary Diagnoses | 2002 |
| 41270_D485 | Diagnoses - ICD10-D48.5 Skin | Health-related outcomes > Hospital inpatient > Summary Diagnoses | 2002 |
| 41270_D486 | Diagnoses - ICD10-D48.6 Breast | Health-related outcomes > Hospital inpatient > Summary Diagnoses | 2002 |
| 41270_D487 | Diagnoses - ICD10-D48.7 Other specified sites | Health-related outcomes > Hospital inpatient > Summary Diagnoses | 2002 |
| 41270_E101 | Diagnoses - ICD10-E10.1 With ketoacidosis | Health-related outcomes > Hospital inpatient > Summary Diagnoses | 2002 |
| 41270_E102 | Diagnoses - ICD10-E10.2 With renal complications | Health-related outcomes > Hospital inpatient > Summary Diagnoses | 2002 |
| 41270_E103 | Diagnoses - ICD10-E10.3 With ophthalmic complications | Health-related outcomes > Hospital inpatient > Summary Diagnoses | 2002 |
| 41270_E104 | Diagnoses - ICD10-E10.4 With neurological complications | Health-related outcomes > Hospital inpatient > Summary Diagnoses | 2002 |
| 41270_E105 | Diagnoses - ICD10-E10.5 With peripheral circulatory complications | Health-related outcomes > Hospital inpatient > Summary Diagnoses | 2002 |
| 41270_E109 | Diagnoses - ICD10-E10.9 Without complications | Health-related outcomes > Hospital inpatient > Summary Diagnoses | 2002 |
| 41270_E110 | Diagnoses - ICD10-E11.0 With coma | Health-related outcomes > Hospital inpatient > Summary Diagnoses | 2002 |
| 41270_E111 | Diagnoses - ICD10-E11.1 With ketoacidosis | Health-related outcomes > Hospital inpatient > Summary Diagnoses | 2002 |
| 41270_E112 | Diagnoses - ICD10-E11.2 With renal complications | Health-related outcomes > Hospital inpatient > Summary Diagnoses | 2002 |
| 41270_E113 | Diagnoses - ICD10-E11.3 With ophthalmic complications | Health-related outcomes > Hospital inpatient > Summary Diagnoses | 2002 |
| 41270_E114 | Diagnoses - ICD10-E11.4 With neurological complications | Health-related outcomes > Hospital inpatient > Summary Diagnoses | 2002 |
| 41270_E115 | Diagnoses - ICD10-E11.5 With peripheral circulatory complications | Health-related outcomes > Hospital inpatient > Summary Diagnoses | 2002 |
| 41270_E116 | Diagnoses - ICD10-E11.6 With other specified complications | Health-related outcomes > Hospital inpatient > Summary Diagnoses | 2002 |
| 41270_E119 | Diagnoses - ICD10-E11.9 Without complications | Health-related outcomes > Hospital inpatient > Summary Diagnoses | 2002 |
| 41270_E139 | Diagnoses - ICD10-E13.9 Without complications | Health-related outcomes > Hospital inpatient > Summary Diagnoses | 2002 |
| 41270_I251 | Diagnoses - ICD10-I25.1 Atherosclerotic heart disease | Health-related outcomes > Hospital inpatient > Summary Diagnoses | 2002 |
| 41270_I252 | Diagnoses - ICD10-I25.2 Old myocardial infarction | Health-related outcomes > Hospital inpatient > Summary Diagnoses | 2002 |
| 41270_I253 | Diagnoses - ICD10-I25.3 Aneurysm of heart | Health-related outcomes > Hospital inpatient > Summary Diagnoses | 2002 |
| 41270_I255 | Diagnoses - ICD10-I25.5 Ischaemic cardiomyopathy | Health-related outcomes > Hospital inpatient > Summary Diagnoses | 2002 |
| 41270_I258 | Diagnoses - ICD10-I25.8 Other forms of chronic ischaemic heart disease | Health-related outcomes > Hospital inpatient > Summary Diagnoses | 2002 |
| 41270_I259 | Diagnoses - ICD10-I25.9 Chronic ischaemic heart disease, unspecified | Health-related outcomes > Hospital inpatient > Summary Diagnoses | 2002 |
| 41270_I420 | Diagnoses - ICD10-I42.0 Dilated cardiomyopathy | Health-related outcomes > Hospital inpatient > Summary Diagnoses | 2002 |
| 41270_I421 | Diagnoses - ICD10-I42.1 Obstructive hypertrophic cardiomyopathy | Health-related outcomes > Hospital inpatient > Summary Diagnoses | 2002 |
| 41270_I422 | Diagnoses - ICD10-I42.2 Other hypertrophic cardiomyopathy | Health-related outcomes > Hospital inpatient > Summary Diagnoses | 2002 |
| 41270_I428 | Diagnoses - ICD10-I42.8 Other cardiomyopathies | Health-related outcomes > Hospital inpatient > Summary Diagnoses | 2002 |
| 41270_I429 | Diagnoses - ICD10-I42.9 Cardiomyopathy, unspecified | Health-related outcomes > Hospital inpatient > Summary Diagnoses | 2002 |
| 41270_I500 | Diagnoses - ICD10-I50.0 Congestive heart failure | Health-related outcomes > Hospital inpatient > Summary Diagnoses | 2002 |
| 41270_I501 | Diagnoses - ICD10-I50.1 Left ventricular failure | Health-related outcomes > Hospital inpatient > Summary Diagnoses | 2002 |
| 41270_I509 | Diagnoses - ICD10-I50.9 Heart failure, unspecified | Health-related outcomes > Hospital inpatient > Summary Diagnoses | 2002 |
| 41270_I601 | Diagnoses - ICD10-I60.1 Subarachnoid haemorrhage from middle cerebral artery | Health-related outcomes > Hospital inpatient > Summary Diagnoses | 2002 |
| 41270_I602 | Diagnoses - ICD10-I60.2 Subarachnoid haemorrhage from anterior communicating artery | Health-related outcomes > Hospital inpatient > Summary Diagnoses | 2002 |
| 41270_I603 | Diagnoses - ICD10-I60.3 Subarachnoid haemorrhage from posterior communicating artery | Health-related outcomes > Hospital inpatient > Summary Diagnoses | 2002 |
| 41270_I609 | Diagnoses - ICD10-I60.9 Subarachnoid haemorrhage, unspecified | Health-related outcomes > Hospital inpatient > Summary Diagnoses | 2002 |
| 41270_I611 | Diagnoses - ICD10-I61.1 Intracerebral haemorrhage in hemisphere, cortical | Health-related outcomes > Hospital inpatient > Summary Diagnoses | 2002 |
| 41270_I615 | Diagnoses - ICD10-I61.5 Intracerebral haemorrhage, intraventricular | Health-related outcomes > Hospital inpatient > Summary Diagnoses | 2002 |
| 41270_I619 | Diagnoses - ICD10-I61.9 Intracerebral haemorrhage, unspecified | Health-related outcomes > Hospital inpatient > Summary Diagnoses | 2002 |
| 41270_I620 | Diagnoses - ICD10-I62.0 Subdural haemorrhage (acute) (nontraumatic) | Health-related outcomes > Hospital inpatient > Summary Diagnoses | 2002 |
| 41270_I629 | Diagnoses - ICD10-I62.9 Intracranial haemorrhage (nontraumatic), unspecified | Health-related outcomes > Hospital inpatient > Summary Diagnoses | 2002 |
| 41270_I632 | Diagnoses - ICD10-I63.2 Cerebral infarction due to unspecified occlusion or stenosis of precerebral arteries | Health-related outcomes > Hospital inpatient > Summary Diagnoses | 2002 |
| 41270_I633 | Diagnoses - ICD10-I63.3 Cerebral infarction due to thrombosis of cerebral arteries | Health-related outcomes > Hospital inpatient > Summary Diagnoses | 2002 |
| 41270_I634 | Diagnoses - ICD10-I63.4 Cerebral infarction due to embolism of cerebral arteries | Health-related outcomes > Hospital inpatient > Summary Diagnoses | 2002 |
| 41270_I635 | Diagnoses - ICD10-I63.5 Cerebral infarction due to unspecified occlusion or stenosis of cerebral arteries | Health-related outcomes > Hospital inpatient > Summary Diagnoses | 2002 |
| 41270_I638 | Diagnoses - ICD10-I63.8 Other cerebral infarction | Health-related outcomes > Hospital inpatient > Summary Diagnoses | 2002 |
| 41270_I639 | Diagnoses - ICD10-I63.9 Cerebral infarction, unspecified | Health-related outcomes > Hospital inpatient > Summary Diagnoses | 2002 |
| 41270_I64 | Diagnoses - ICD10-I64 Stroke, not specified as haemorrhage or infarction | Health-related outcomes > Hospital inpatient > Summary Diagnoses | 2002 |
| 41270_I652 | Diagnoses - ICD10-I65.2 Occlusion and stenosis of carotid artery | Health-related outcomes > Hospital inpatient > Summary Diagnoses | 2002 |
| 41270_I671 | Diagnoses - ICD10-I67.1 Cerebral aneurysm, nonruptured | Health-related outcomes > Hospital inpatient > Summary Diagnoses | 2002 |
| 41270_I672 | Diagnoses - ICD10-I67.2 Cerebral atherosclerosis | Health-related outcomes > Hospital inpatient > Summary Diagnoses | 2002 |
| 41270_I678 | Diagnoses - ICD10-I67.8 Other specified cerebrovascular diseases | Health-related outcomes > Hospital inpatient > Summary Diagnoses | 2002 |
| 41270_I679 | Diagnoses - ICD10-I67.9 Cerebrovascular disease, unspecified | Health-related outcomes > Hospital inpatient > Summary Diagnoses | 2002 |
| 41270_I690 | Diagnoses - ICD10-I69.0 Sequelae of subarachnoid haemorrhage | Health-related outcomes > Hospital inpatient > Summary Diagnoses | 2002 |
| 41270_I691 | Diagnoses - ICD10-I69.1 Sequelae of intracerebral haemorrhage | Health-related outcomes > Hospital inpatient > Summary Diagnoses | 2002 |
| 41270_I693 | Diagnoses - ICD10-I69.3 Sequelae of cerebral infarction | Health-related outcomes > Hospital inpatient > Summary Diagnoses | 2002 |
| 41270_I694 | Diagnoses - ICD10-I69.4 Sequelae of stroke, not specified as haemorrhage or infarction | Health-related outcomes > Hospital inpatient > Summary Diagnoses | 2002 |
| 41270_I698 | Diagnoses - ICD10-I69.8 Sequelae of other and unspecified cerebrovascular diseases | Health-related outcomes > Hospital inpatient > Summary Diagnoses | 2002 |
| 41270_J440 | Diagnoses - ICD10-J44.0 Chronic obstructive pulmonary disease with acute lower respiratory infection | Health-related outcomes > Hospital inpatient > Summary Diagnoses | 2002 |
| 41270_J441 | Diagnoses - ICD10-J44.1 Chronic obstructive pulmonary disease with acute exacerbation, unspecified | Health-related outcomes > Hospital inpatient > Summary Diagnoses | 2002 |
| 41270_J448 | Diagnoses - ICD10-J44.8 Other specified chronic obstructive pulmonary disease | Health-related outcomes > Hospital inpatient > Summary Diagnoses | 2002 |
| 41270_J449 | Diagnoses - ICD10-J44.9 Chronic obstructive pulmonary disease, unspecified | Health-related outcomes > Hospital inpatient > Summary Diagnoses | 2002 |
| 41270_N180 | Diagnoses - ICD10-N18.0 End-stage renal disease | Health-related outcomes > Hospital inpatient > Summary Diagnoses | 2002 |
| 41270_N182 | Diagnoses - ICD10-N18.2 Chronic kidney disease, stage 2 | Health-related outcomes > Hospital inpatient > Summary Diagnoses | 2002 |
| 41270_N183 | Diagnoses - ICD10-N18.3 Chronic kidney disease, stage 3 | Health-related outcomes > Hospital inpatient > Summary Diagnoses | 2002 |
| 41270_N184 | Diagnoses - ICD10-N18.4 Chronic kidney disease, stage 4 | Health-related outcomes > Hospital inpatient > Summary Diagnoses | 2002 |
| 41270_N185 | Diagnoses - ICD10-N18.5 Chronic kidney disease, stage 5 | Health-related outcomes > Hospital inpatient > Summary Diagnoses | 2002 |
| 41270_N189 | Diagnoses - ICD10-N18.9 Chronic renal failure, unspecified | Health-related outcomes > Hospital inpatient > Summary Diagnoses | 2002 |
| 41271_C509 | Diagnoses - ICD9-C50.9 Breast, unspecified | Health-related outcomes > Hospital inpatient > Summary Diagnoses | 2002 |
| 41271_D179 | Diagnoses - ICD9-D17.9 Benign lipomatous neoplasm, unspecified | Health-related outcomes > Hospital inpatient > Summary Diagnoses | 2002 |
| 41271_D233 | Diagnoses - ICD9-D23.3 Skin of other and unspecified parts of face | Health-related outcomes > Hospital inpatient > Summary Diagnoses | 2002 |
| 41271_D235 | Diagnoses - ICD9-D23.5 Skin of trunk | Health-related outcomes > Hospital inpatient > Summary Diagnoses | 2002 |
| 41271_D237 | Diagnoses - ICD9-D23.7 Skin of lower limb, including hip | Health-related outcomes > Hospital inpatient > Summary Diagnoses | 2002 |
| 41271_D24 | Diagnoses - ICD9-D24 Benign neoplasm of breast | Health-related outcomes > Hospital inpatient > Summary Diagnoses | 2002 |
| 41271_D259 | Diagnoses - ICD9-D25.9 Leiomyoma of uterus, unspecified | Health-related outcomes > Hospital inpatient > Summary Diagnoses | 2002 |
| 41271_I259 | Diagnoses - ICD9-I25.9 Chronic ischaemic heart disease, unspecified | Health-related outcomes > Hospital inpatient > Summary Diagnoses | 2002 |

**Supplementary Table S1.** Five hundred and one variables encoding relevant medical risk factors, for inclusion in the base classifiers. Labels in “code” column are the outcome of curation through PHESANT, and relate to the UK Biobank data dictionary at https://biobank.ctsu.ox.ac.uk/crystal/exinfo.cgi?src=accessing_data_guide.


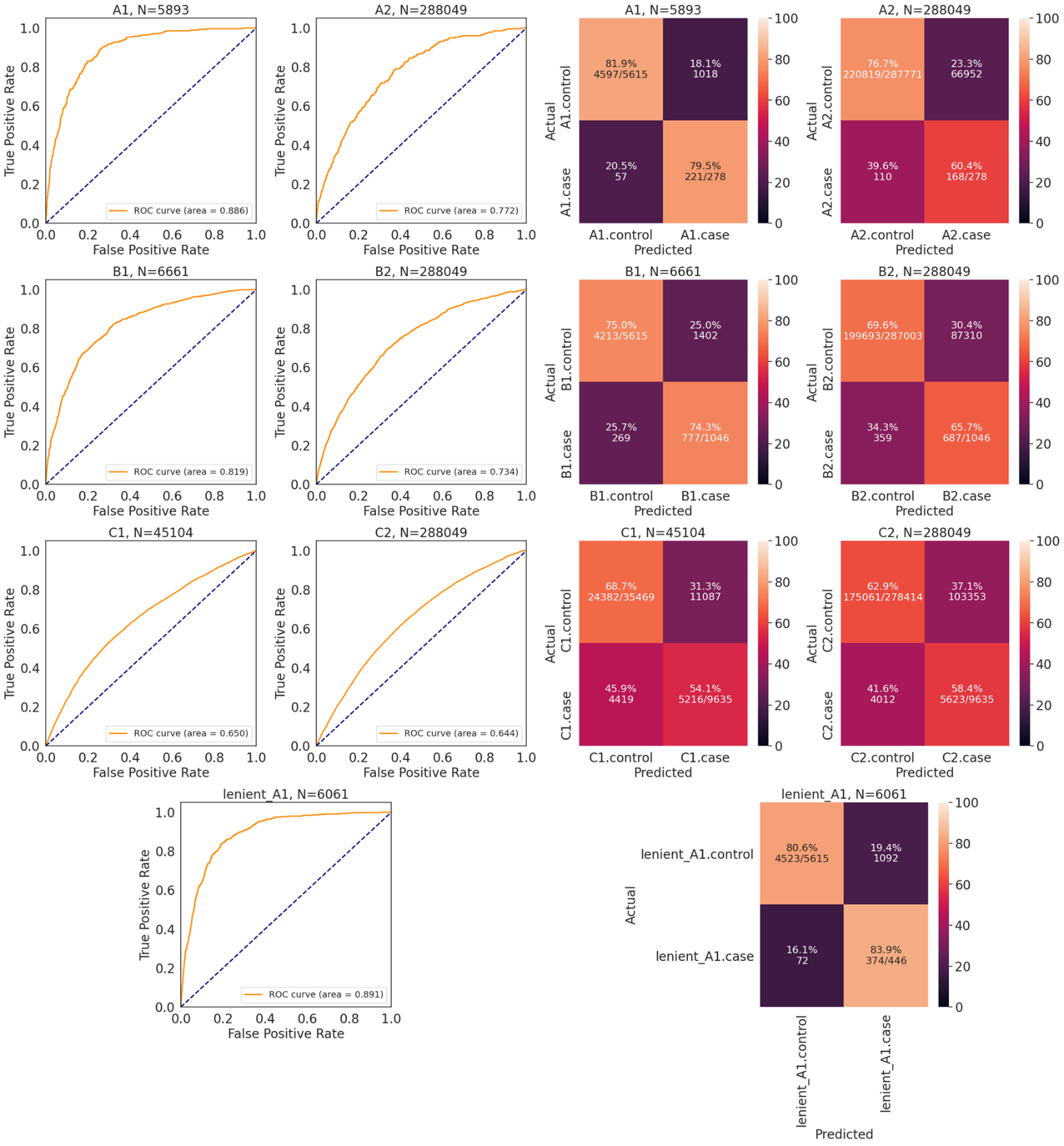


**Supplementary Figure S1.** Receiver-operating characteristic curve and confusion matrices for the COVID-19 case-control classifiers using XGBoost.

| **Target** | **Base** | **Threshold** | **PRS.R2** | **Full.R2** | **Null.R2** | **Coef.** | **S.E.** | **P** | **N SNP** | **Bonf. P** | **Significance** |
| --- | --- | --- | --- | --- | --- | --- | --- | --- | --- | --- | --- |
| A1 | A2_ALL_eur_leave_ukbb_23andme_20210107 | 5.01E-05 | 0.005103 | 0.302848 | 0.297744 | 39.1717 | 12.068 | 0.001171 | 95 | 0.065564 | NS |
| A1 | A2_ALL_leave_UKBB_23andme_20210107 | 0.0004 | 0.009094 | 0.306838 | 0.297744 | 130.618 | 30.2865 | 1.61E-05 | 385 | 0.000903 | * |
| A1 | B1_ALL_eur_leave_ukbb_23andme_20210107 | 0.00355 | 0.00097 | 0.298714 | 0.297744 | 99.6433 | 70.6047 | 0.158161 | 3664 | 1 | NS |
| A1 | B1_ALL_leave_UKBB_23andme_20210107 | 5.01E-05 | 0.002958 | 0.300702 | 0.297744 | 18.7551 | 7.58628 | 0.013427 | 54 | 0.751906 | NS |
| A1 | B2_ALL_eur_leave_ukbb_23andme_20210107 | 5.00E-08 | 0.000861 | 0.298605 | 0.297744 | 2.9213 | 2.16189 | 0.176607 | 6 | 1 | NS |
| A1 | B2_ALL_leave_UKBB_23andme_20210107 | 5.00E-08 | 0.001311 | 0.299055 | 0.297744 | 3.02836 | 1.80787 | 0.093915 | 5 | 1 | NS |
| A1 | C2_ALL_eur_leave_ukbb_23andme_20210107 | 0.0014 | 0.001711 | 0.299455 | 0.297744 | -328.928 | 175.516 | 0.060922 | 1654 | 1 | NS |
| A1 | C2_ALL_leave_UKBB_23andme_20210107 | 0.0033 | 0.004351 | 0.302095 | 0.297744 | -862.892 | 289.566 | 0.002883 | 2993 | 0.161446 | NS |
| A2 | A2_ALL_eur_leave_ukbb_23andme_20210107 | 5.01E-05 | 0.003385 | 0.062833 | 0.059448 | 40.8602 | 10.5103 | 0.000101 | 95 | 0.005668 | * |
| A2 | A2_ALL_leave_UKBB_23andme_20210107 | 0.00025 | 0.005555 | 0.065002 | 0.059448 | 107.832 | 21.8209 | 7.74E-07 | 253 | 4.34E-05 | * |
| A2 | B1_ALL_eur_leave_ukbb_23andme_20210107 | 0.00355 | 0.000294 | 0.059741 | 0.059448 | 70.6228 | 62.2173 | 0.256334 | 3664 | 1 | NS |
| A2 | B1_ALL_leave_UKBB_23andme_20210107 | 5.01E-05 | 0.001087 | 0.060534 | 0.059448 | 15.2628 | 6.95232 | 0.028138 | 54 | 1 | NS |
| A2 | B2_ALL_eur_leave_ukbb_23andme_20210107 | 0.0001 | 0.001254 | 0.060702 | 0.059448 | 46.605 | 19.7391 | 0.018223 | 169 | 1 | NS |
| A2 | B2_ALL_leave_UKBB_23andme_20210107 | 0.02235 | 0.000955 | 0.060403 | 0.059448 | 653.441 | 319.126 | 0.0406 | 13097 | 1 | NS |
| A2 | C2_ALL_eur_leave_ukbb_23andme_20210107 | 0.2307 | 0.00109 | 0.060537 | 0.059448 | 4589.52 | 2098.68 | 0.028753 | 124363 | 1 | NS |
| A2 | C2_ALL_leave_UKBB_23andme_20210107 | 0.0033 | 0.001054 | 0.060501 | 0.059448 | -549.606 | 255.75 | 0.031635 | 2993 | 1 | NS |
| B1 | A2_ALL_eur_leave_ukbb_23andme_20210107 | 0.02865 | 0.001827 | 0.255501 | 0.253674 | 439.007 | 152.637 | 0.004025 | 24366 | 0.225427 | NS |
| B1 | A2_ALL_leave_UKBB_23andme_20210107 | 0.001 | 0.004188 | 0.257862 | 0.253674 | 119.731 | 27.5226 | 1.36E-05 | 867 | 0.000761 | * |
| B1 | B1_ALL_eur_leave_ukbb_23andme_20210107 | 0.0139 | 0.001558 | 0.255232 | 0.253674 | 204.295 | 76.8837 | 0.007879 | 12343 | 0.441237 | NS |
| B1 | B1_ALL_leave_UKBB_23andme_20210107 | 5.00E-08 | 0.001936 | 0.25561 | 0.253674 | 1.42176 | 0.472488 | 0.00262 | 1 | 0.146741 | NS |
| B1 | B2_ALL_eur_leave_ukbb_23andme_20210107 | 0.0003 | 0.000635 | 0.254309 | 0.253674 | 36.7474 | 21.6462 | 0.089576 | 450 | 1 | NS |
| B1 | B2_ALL_leave_UKBB_23andme_20210107 | 0.00065 | 0.001705 | 0.255379 | 0.253674 | 91.4987 | 32.9037 | 0.005422 | 623 | 0.303653 | NS |
| B1 | C2_ALL_eur_leave_ukbb_23andme_20210107 | 0.35 | 0.000467 | 0.25414 | 0.253674 | 2269.64 | 1559.12 | 0.14547 | 166719 | 1 | NS |
| B1 | C2_ALL_leave_UKBB_23andme_20210107 | 0.00325 | 0.000629 | 0.254303 | 0.253674 | -266.145 | 157.645 | 0.091362 | 2947 | 1 | NS |
| B2 | A2_ALL_eur_leave_ukbb_23andme_20210107 | 0.0293 | 0.001015 | 0.026653 | 0.025637 | 477.93 | 128.933 | 0.00021 | 24854 | 0.011754 | * |
| B2 | A2_ALL_leave_UKBB_23andme_20210107 | 0.001 | 0.001617 | 0.027255 | 0.025637 | 108.095 | 23.1015 | 2.88E-06 | 867 | 0.000161 | * |
| B2 | B1_ALL_eur_leave_ukbb_23andme_20210107 | 0.01395 | 0.000317 | 0.025954 | 0.025637 | 132.842 | 64.1745 | 0.038451 | 12376 | 1 | NS |
| B2 | B1_ALL_leave_UKBB_23andme_20210107 | 5.00E-08 | 0.001159 | 0.026797 | 0.025637 | 1.58144 | 0.38325 | 3.68E-05 | 1 | 0.002064 | * |
| B2 | B2_ALL_eur_leave_ukbb_23andme_20210107 | 0.0003 | 0.000837 | 0.026475 | 0.025637 | 62.507 | 18.5203 | 0.000738 | 450 | 0.041329 | * |
| B2 | B2_ALL_leave_UKBB_23andme_20210107 | 0.0006 | 0.001175 | 0.026813 | 0.025637 | 107.906 | 27.0069 | 6.46E-05 | 593 | 0.003615 | * |
| B2 | C2_ALL_eur_leave_ukbb_23andme_20210107 | 0.5 | 0.000818 | 0.026455 | 0.025637 | 5458.26 | 1640.63 | 0.000878 | 209543 | 0.049172 | * |
| B2 | C2_ALL_leave_UKBB_23andme_20210107 | 0.4338 | 0.000703 | 0.02634 | 0.025637 | 4576 | 1483.98 | 0.002045 | 141066 | 0.114524 | NS |
| C1 | A2_ALL_eur_leave_ukbb_23andme_20210107 | 0.0867 | 0.000382 | 0.05949 | 0.059108 | 284.23 | 83.594 | 0.000674 | 60883 | 0.037718 | * |
| C1 | A2_ALL_leave_UKBB_23andme_20210107 | 0.07895 | 0.000429 | 0.059537 | 0.059108 | 279.198 | 77.5153 | 0.000316 | 35981 | 0.017694 | * |
| C1 | B1_ALL_eur_leave_ukbb_23andme_20210107 | 1 | 4.39E-05 | 0.059152 | 0.059108 | 249.606 | 216.526 | 0.249003 | 302583 | 1 | NS |
| C1 | B1_ALL_leave_UKBB_23andme_20210107 | 5.00E-08 | 0.000313 | 0.059421 | 0.059108 | 0.498373 | 0.160865 | 0.001948 | 1 | 0.109073 | NS |
| C1 | B2_ALL_eur_leave_ukbb_23andme_20210107 | 0.1473 | 0.000247 | 0.059355 | 0.059108 | 413.222 | 151.197 | 0.006276 | 84971 | 0.351449 | NS |
| C1 | B2_ALL_leave_UKBB_23andme_20210107 | 0.0023 | 0.000317 | 0.059425 | 0.059108 | 62.9009 | 20.3072 | 0.001952 | 1900 | 0.109299 | NS |
| C1 | C2_ALL_eur_leave_ukbb_23andme_20210107 | 5.00E-08 | 0.000842 | 0.05995 | 0.059108 | 5.47313 | 1.08117 | 4.14E-07 | 5 | 2.32E-05 | * |
| C1 | C2_ALL_leave_UKBB_23andme_20210107 | 5.00E-08 | 0.000887 | 0.059996 | 0.059108 | 6.0974 | 1.17286 | 2.01E-07 | 5 | 1.12E-05 | * |
| C2 | A2_ALL_eur_leave_ukbb_23andme_20210107 | 0.07995 | 0.000202 | 0.016207 | 0.016005 | 274.161 | 71.1333 | 0.000116 | 57087 | 0.006502 | * |
| C2 | A2_ALL_leave_UKBB_23andme_20210107 | 0.3349 | 0.00019 | 0.016195 | 0.016005 | 523.551 | 140.068 | 0.000186 | 99022 | 0.010395 | * |
| C2 | B1_ALL_eur_leave_ukbb_23andme_20210107 | 1 | 2.72E-05 | 0.016032 | 0.016005 | 270.757 | 191.517 | 0.157437 | 302583 | 1 | NS |
| C2 | B1_ALL_leave_UKBB_23andme_20210107 | 5.00E-08 | 0.000112 | 0.016117 | 0.016005 | 0.409644 | 0.141218 | 0.003722 | 1 | 0.208442 | NS |
| C2 | B2_ALL_eur_leave_ukbb_23andme_20210107 | 0.0966 | 0.000142 | 0.016147 | 0.016005 | 352.548 | 109.093 | 0.001231 | 61676 | 0.068928 | NS |
| C2 | B2_ALL_leave_UKBB_23andme_20210107 | 0.0044 | 0.000199 | 0.016204 | 0.016005 | 94.0676 | 24.5927 | 0.000131 | 3290 | 0.007323 | * |
| C2 | C2_ALL_eur_leave_ukbb_23andme_20210107 | 5.00E-08 | 0.00044 | 0.016445 | 0.016005 | 5.48472 | 0.959181 | 1.08E-08 | 5 | 6.03E-07 | * |
| C2 | C2_ALL_leave_UKBB_23andme_20210107 | 5.00E-08 | 0.000466 | 0.016471 | 0.016005 | 6.11816 | 1.0395 | 3.96E-09 | 5 | 2.22E-07 | * |
| lenient.A1 | A2_ALL_eur_leave_ukbb_23andme_20210107 | 0.0004 | 0.00305 | 0.346475 | 0.343425 | 78.7005 | 26.5794 | 0.003067 | 567 | 0.171742 | NS |
| lenient.A1 | A2_ALL_leave_UKBB_23andme_20210107 | 0.001 | 0.007562 | 0.350987 | 0.343425 | 190.746 | 40.9835 | 3.25E-06 | 867 | 0.000182 | * |
| lenient.A1 | B1_ALL_eur_leave_ukbb_23andme_20210107 | 0.0008 | 0.001053 | 0.344478 | 0.343425 | 46.3999 | 26.6417 | 0.081573 | 933 | 1 | NS |
| lenient.A1 | B1_ALL_leave_UKBB_23andme_20210107 | 5.00E-08 | 0.001336 | 0.344761 | 0.343425 | 1.38674 | 0.694212 | 0.045762 | 1 | 1 | NS |
| lenient.A1 | B2_ALL_eur_leave_ukbb_23andme_20210107 | 0.1062 | 0.000743 | 0.344168 | 0.343425 | -886.302 | 605.889 | 0.143518 | 66333 | 1 | NS |
| lenient.A1 | B2_ALL_leave_UKBB_23andme_20210107 | 5.00E-08 | 0.001178 | 0.344603 | 0.343425 | 2.89092 | 1.5453 | 0.061375 | 5 | 1 | NS |
| lenient.A1 | C2_ALL_eur_leave_ukbb_23andme_20210107 | 5.00E-08 | 0.000794 | 0.344219 | 0.343425 | 7.54995 | 4.97311 | 0.128975 | 5 | 1 | NS |
| lenient.A1 | C2_ALL_leave_UKBB_23andme_20210107 | 0.0033 | 0.001741 | 0.345166 | 0.343425 | -536.191 | 239.813 | 0.02536 | 2993 | 1 | NS |

**Supplementary Table S2.** Results of best-fitting thresholds for each of the 56 combinations of target phenotypes and base summary statistics.

Notes: Coef.: beta coefficient, S.E.: standard error, N SNP: number of markers in PS, Bonf. P.: Bonferroni-adjusted *p*-value.

| basepheno | targetpheno | coef | std err | t | P>\|t\| | -log10(p) |
| --- | --- | --- | --- | --- | --- | --- |
| A1 | B2_ALL_eur_leave_ukbb_23andme_20210107-lenientA1 | -115.5812 | 164.176 | -0.704 | 0.482 | 0.317 |
| A1 | A2_ALL_leave_UKBB_23andme_20210107-B1 | -13.8466 | 10.647 | -1.301 | 0.194 | 0.712 |
| A1 | B1_ALL_leave_UKBB_23andme_20210107-A2 | -2.7991 | 1.651 | -1.695 | 0.091 | 1.041 |
| A1 | B1_ALL_leave_UKBB_23andme_20210107-B1 | -0.061 | 0.187 | -0.326 | 0.744 | 0.128 |
| A1 | A2_ALL_leave_UKBB_23andme_20210107-A2 | -0.1253 | 5.14 | -0.024 | 0.981 | 0.008 |
| A1 | B2_ALL_leave_UKBB_23andme_20210107-A1 | -0.1898 | 0.404 | -0.469 | 0.639 | 0.194 |
| A1 | B2_ALL_eur_leave_ukbb_23andme_20210107-B1 | -7.8616 | 9.161 | -0.858 | 0.392 | 0.407 |
| A1 | A2_ALL_eur_leave_ukbb_23andme_20210107-B2 | -31.4326 | 60.915 | -0.516 | 0.606 | 0.218 |
| A1 | B1_ALL_eur_leave_ukbb_23andme_20210107-C1 | -445.384 | 311.664 | -1.429 | 0.154 | 0.812 |
| A1 | A2_ALL_leave_UKBB_23andme_20210107-lenientA1 | -13.8466 | 10.647 | -1.301 | 0.194 | 0.712 |
| A1 | A2_ALL_eur_leave_ukbb_23andme_20210107-C1 | -73.5843 | 102.561 | -0.717 | 0.474 | 0.324 |
| A1 | B1_ALL_eur_leave_ukbb_23andme_20210107-B2 | -18.0947 | 30.888 | -0.586 | 0.558 | 0.253 |
| A1 | B1_ALL_leave_UKBB_23andme_20210107-lenientA1 | -0.061 | 0.187 | -0.326 | 0.744 | 0.128 |
| A1 | C2_ALL_eur_leave_ukbb_23andme_20210107-A1 | -56.9244 | 41.412 | -1.375 | 0.17 | 0.77 |
| A1 | B2_ALL_eur_leave_ukbb_23andme_20210107-A2 | 1.1282 | 4.798 | 0.235 | 0.814 | 0.089 |
| A1 | C2_ALL_leave_UKBB_23andme_20210107-C2 | -1.1711 | 1.394 | -0.84 | 0.401 | 0.397 |
| A1 | B1_ALL_eur_leave_ukbb_23andme_20210107-A1 | 1.2068 | 15.729 | 0.077 | 0.939 | 0.027 |
| A1 | A2_ALL_eur_leave_ukbb_23andme_20210107-A1 | -0.6759 | 2.875 | -0.235 | 0.814 | 0.089 |
| A1 | B1_ALL_leave_UKBB_23andme_20210107-C2 | -0.061 | 0.187 | -0.326 | 0.744 | 0.128 |
| A1 | B2_ALL_leave_UKBB_23andme_20210107-B2 | -18.7111 | 12.561 | -1.49 | 0.137 | 0.863 |
| A1 | C2_ALL_leave_UKBB_23andme_20210107-B1 | 8.0716 | 64.396 | 0.125 | 0.9 | 0.046 |
| A1 | C2_ALL_leave_UKBB_23andme_20210107-A2 | 0.7316 | 64.665 | 0.011 | 0.991 | 0.004 |
| A1 | B2_ALL_leave_UKBB_23andme_20210107-C1 | -52.1965 | 25.721 | -2.029 | 0.043 | 1.367 |
| A1 | A2_ALL_leave_UKBB_23andme_20210107-C2 | 19.3365 | 192.632 | 0.1 | 0.92 | 0.036 |
| A1 | C2_ALL_leave_UKBB_23andme_20210107-lenientA1 | 0.7316 | 64.665 | 0.011 | 0.991 | 0.004 |
| A1 | C2_ALL_eur_leave_ukbb_23andme_20210107-B2 | 424.9448 | 750.532 | 0.566 | 0.572 | 0.243 |
| A1 | C2_ALL_eur_leave_ukbb_23andme_20210107-C1 | -1.0395 | 1.283 | -0.81 | 0.418 | 0.379 |
| A1 | B2_ALL_eur_leave_ukbb_23andme_20210107-C2 | -137.83 | 155.83 | -0.884 | 0.377 | 0.424 |
| A1 | C2_ALL_eur_leave_ukbb_23andme_20210107-lenientA1 | -1.0395 | 1.283 | -0.81 | 0.418 | 0.379 |
| A1 | C2_ALL_leave_UKBB_23andme_20210107-A1 | 0.7316 | 64.665 | 0.011 | 0.991 | 0.004 |
| A1 | B2_ALL_leave_UKBB_23andme_20210107-A2 | -135.1461 | 76.576 | -1.765 | 0.079 | 1.102 |
| A1 | A2_ALL_leave_UKBB_23andme_20210107-C1 | -54.3428 | 95.351 | -0.57 | 0.569 | 0.245 |
| A1 | B1_ALL_leave_UKBB_23andme_20210107-B2 | -0.061 | 0.187 | -0.326 | 0.744 | 0.128 |
| A1 | A2_ALL_leave_UKBB_23andme_20210107-B2 | -13.8466 | 10.647 | -1.301 | 0.194 | 0.712 |
| A1 | B2_ALL_leave_UKBB_23andme_20210107-B1 | -20.3 | 13.007 | -1.561 | 0.12 | 0.921 |
| A1 | B1_ALL_leave_UKBB_23andme_20210107-C1 | -0.061 | 0.187 | -0.326 | 0.744 | 0.128 |
| A1 | C2_ALL_eur_leave_ukbb_23andme_20210107-A2 | 155.8568 | 485.107 | 0.321 | 0.748 | 0.126 |
| A1 | B2_ALL_eur_leave_ukbb_23andme_20210107-C1 | -173.7009 | 191.444 | -0.907 | 0.365 | 0.438 |
| A1 | A2_ALL_eur_leave_ukbb_23andme_20210107-C2 | -48.0035 | 98.022 | -0.49 | 0.625 | 0.204 |
| A1 | B2_ALL_eur_leave_ukbb_23andme_20210107-B2 | -7.8616 | 9.161 | -0.858 | 0.392 | 0.407 |
| A1 | C2_ALL_eur_leave_ukbb_23andme_20210107-B1 | 101.1487 | 612.19 | 0.165 | 0.869 | 0.061 |
| A1 | B2_ALL_leave_UKBB_23andme_20210107-lenientA1 | -0.1898 | 0.404 | -0.469 | 0.639 | 0.194 |
| A1 | B1_ALL_eur_leave_ukbb_23andme_20210107-C2 | -445.384 | 311.664 | -1.429 | 0.154 | 0.812 |
| A1 | B1_ALL_eur_leave_ukbb_23andme_20210107-B1 | -17.9717 | 30.822 | -0.583 | 0.56 | 0.252 |
| A1 | A2_ALL_eur_leave_ukbb_23andme_20210107-A2 | -0.6759 | 2.875 | -0.235 | 0.814 | 0.089 |
| A1 | B2_ALL_eur_leave_ukbb_23andme_20210107-A1 | -0.388 | 0.5 | -0.777 | 0.438 | 0.359 |
| A1 | B1_ALL_eur_leave_ukbb_23andme_20210107-A2 | 1.2068 | 15.729 | 0.077 | 0.939 | 0.027 |
| A1 | A2_ALL_eur_leave_ukbb_23andme_20210107-B1 | -42.9859 | 59.863 | -0.718 | 0.473 | 0.325 |
| A1 | B2_ALL_leave_UKBB_23andme_20210107-C2 | -42.0321 | 36.393 | -1.155 | 0.249 | 0.604 |
| A1 | C2_ALL_leave_UKBB_23andme_20210107-C1 | -1.1711 | 1.394 | -0.84 | 0.401 | 0.397 |
| A1 | C2_ALL_leave_UKBB_23andme_20210107-B2 | -245.139 | 714.517 | -0.343 | 0.732 | 0.135 |
| A1 | C2_ALL_eur_leave_ukbb_23andme_20210107-C2 | -1.0395 | 1.283 | -0.81 | 0.418 | 0.379 |
| A1 | A2_ALL_leave_UKBB_23andme_20210107-A1 | -0.8627 | 6.825 | -0.126 | 0.9 | 0.046 |
| A1 | B1_ALL_leave_UKBB_23andme_20210107-A1 | -2.7991 | 1.651 | -1.695 | 0.091 | 1.041 |
| A1 | A2_ALL_eur_leave_ukbb_23andme_20210107-lenientA1 | -0.15 | 7.611 | -0.02 | 0.984 | 0.007 |
| A1 | B1_ALL_eur_leave_ukbb_23andme_20210107-lenientA1 | -12.9373 | 7.306 | -1.771 | 0.078 | 1.108 |
| A2 | B2_ALL_eur_leave_ukbb_23andme_20210107-lenientA1 | -32.5571 | 161.014 | -0.202 | 0.84 | 0.076 |
| A2 | A2_ALL_leave_UKBB_23andme_20210107-B1 | -14.4827 | 10.428 | -1.389 | 0.166 | 0.78 |
| A2 | B1_ALL_leave_UKBB_23andme_20210107-A2 | -2.802 | 1.618 | -1.732 | 0.084 | 1.076 |
| A2 | B1_ALL_leave_UKBB_23andme_20210107-B1 | 0.037 | 0.183 | 0.202 | 0.84 | 0.076 |
| A2 | A2_ALL_leave_UKBB_23andme_20210107-A2 | 0.8013 | 5.037 | 0.159 | 0.874 | 0.058 |
| A2 | B2_ALL_leave_UKBB_23andme_20210107-A1 | -0.0357 | 0.396 | -0.09 | 0.928 | 0.032 |
| A2 | B2_ALL_eur_leave_ukbb_23andme_20210107-B1 | -4.4683 | 8.985 | -0.497 | 0.619 | 0.208 |
| A2 | A2_ALL_eur_leave_ukbb_23andme_20210107-B2 | -55.1644 | 59.629 | -0.925 | 0.356 | 0.449 |
| A2 | B1_ALL_eur_leave_ukbb_23andme_20210107-C1 | -139.6172 | 306.423 | -0.456 | 0.649 | 0.188 |
| A2 | A2_ALL_leave_UKBB_23andme_20210107-lenientA1 | -14.4827 | 10.428 | -1.389 | 0.166 | 0.78 |
| A2 | A2_ALL_eur_leave_ukbb_23andme_20210107-C1 | -90.3592 | 100.45 | -0.9 | 0.369 | 0.433 |
| A2 | B1_ALL_eur_leave_ukbb_23andme_20210107-B2 | 2.7242 | 30.286 | 0.09 | 0.928 | 0.032 |
| A2 | B1_ALL_leave_UKBB_23andme_20210107-lenientA1 | 0.037 | 0.183 | 0.202 | 0.84 | 0.076 |
| A2 | C2_ALL_eur_leave_ukbb_23andme_20210107-A1 | -10.7132 | 40.714 | -0.263 | 0.793 | 0.101 |
| A2 | B2_ALL_eur_leave_ukbb_23andme_20210107-A2 | 5.0563 | 4.693 | 1.077 | 0.282 | 0.55 |
| A2 | C2_ALL_leave_UKBB_23andme_20210107-C2 | -0.2865 | 1.367 | -0.21 | 0.834 | 0.079 |
| A2 | B1_ALL_eur_leave_ukbb_23andme_20210107-A1 | 6.4902 | 15.408 | 0.421 | 0.674 | 0.171 |
| A2 | A2_ALL_eur_leave_ukbb_23andme_20210107-A1 | -0.3229 | 2.818 | -0.115 | 0.909 | 0.041 |
| A2 | B1_ALL_leave_UKBB_23andme_20210107-C2 | 0.037 | 0.183 | 0.202 | 0.84 | 0.076 |
| A2 | B2_ALL_leave_UKBB_23andme_20210107-B2 | -1.0143 | 12.358 | -0.082 | 0.935 | 0.029 |
| A2 | C2_ALL_leave_UKBB_23andme_20210107-B1 | 77.7526 | 62.932 | 1.236 | 0.218 | 0.662 |
| A2 | C2_ALL_leave_UKBB_23andme_20210107-A2 | 73.7934 | 63.211 | 1.167 | 0.244 | 0.613 |
| A2 | B2_ALL_leave_UKBB_23andme_20210107-C1 | -29.5189 | 25.33 | -1.165 | 0.245 | 0.611 |
| A2 | A2_ALL_leave_UKBB_23andme_20210107-C2 | 44.7853 | 188.751 | 0.237 | 0.813 | 0.09 |
| A2 | C2_ALL_leave_UKBB_23andme_20210107-lenientA1 | 73.7934 | 63.211 | 1.167 | 0.244 | 0.613 |
| A2 | C2_ALL_eur_leave_ukbb_23andme_20210107-B2 | 813.6552 | 734.266 | 1.108 | 0.269 | 0.57 |
| A2 | C2_ALL_eur_leave_ukbb_23andme_20210107-C1 | -0.329 | 1.258 | -0.261 | 0.794 | 0.1 |
| A2 | B2_ALL_eur_leave_ukbb_23andme_20210107-C2 | -39.0931 | 152.901 | -0.256 | 0.798 | 0.098 |
| A2 | C2_ALL_eur_leave_ukbb_23andme_20210107-lenientA1 | -0.329 | 1.258 | -0.261 | 0.794 | 0.1 |
| A2 | C2_ALL_leave_UKBB_23andme_20210107-A1 | 73.7934 | 63.211 | 1.167 | 0.244 | 0.613 |
| A2 | B2_ALL_leave_UKBB_23andme_20210107-A2 | -66.7992 | 75.354 | -0.886 | 0.376 | 0.425 |
| A2 | A2_ALL_leave_UKBB_23andme_20210107-C1 | -46.7448 | 93.45 | -0.5 | 0.617 | 0.21 |
| A2 | B1_ALL_leave_UKBB_23andme_20210107-B2 | 0.037 | 0.183 | 0.202 | 0.84 | 0.076 |
| A2 | A2_ALL_leave_UKBB_23andme_20210107-B2 | -14.4827 | 10.428 | -1.389 | 0.166 | 0.78 |
| A2 | B2_ALL_leave_UKBB_23andme_20210107-B1 | -4.1549 | 12.8 | -0.325 | 0.746 | 0.127 |
| A2 | B1_ALL_leave_UKBB_23andme_20210107-C1 | 0.037 | 0.183 | 0.202 | 0.84 | 0.076 |
| A2 | C2_ALL_eur_leave_ukbb_23andme_20210107-A2 | 405.5619 | 474.834 | 0.854 | 0.394 | 0.405 |
| A2 | B2_ALL_eur_leave_ukbb_23andme_20210107-C1 | -58.9479 | 187.848 | -0.314 | 0.754 | 0.123 |
| A2 | A2_ALL_eur_leave_ukbb_23andme_20210107-C2 | -71.3863 | 96 | -0.744 | 0.458 | 0.339 |
| A2 | B2_ALL_eur_leave_ukbb_23andme_20210107-B2 | -4.4683 | 8.985 | -0.497 | 0.619 | 0.208 |
| A2 | C2_ALL_eur_leave_ukbb_23andme_20210107-B1 | 452.1039 | 599.318 | 0.754 | 0.451 | 0.346 |
| A2 | B2_ALL_leave_UKBB_23andme_20210107-lenientA1 | -0.0357 | 0.396 | -0.09 | 0.928 | 0.032 |
| A2 | B1_ALL_eur_leave_ukbb_23andme_20210107-C2 | -139.6172 | 306.423 | -0.456 | 0.649 | 0.188 |
| A2 | B1_ALL_eur_leave_ukbb_23andme_20210107-B1 | 3.1875 | 30.222 | 0.105 | 0.916 | 0.038 |
| A2 | A2_ALL_eur_leave_ukbb_23andme_20210107-A2 | -0.3229 | 2.818 | -0.115 | 0.909 | 0.041 |
| A2 | B2_ALL_eur_leave_ukbb_23andme_20210107-A1 | -0.1947 | 0.49 | -0.397 | 0.691 | 0.161 |
| A2 | B1_ALL_eur_leave_ukbb_23andme_20210107-A2 | 6.4902 | 15.408 | 0.421 | 0.674 | 0.171 |
| A2 | A2_ALL_eur_leave_ukbb_23andme_20210107-B1 | -61.7957 | 58.598 | -1.055 | 0.293 | 0.533 |
| A2 | B2_ALL_leave_UKBB_23andme_20210107-C2 | -14.6697 | 35.738 | -0.41 | 0.682 | 0.166 |
| A2 | C2_ALL_leave_UKBB_23andme_20210107-C1 | -0.2865 | 1.367 | -0.21 | 0.834 | 0.079 |
| A2 | C2_ALL_leave_UKBB_23andme_20210107-B2 | 24.029 | 700.327 | 0.034 | 0.973 | 0.012 |
| A2 | C2_ALL_eur_leave_ukbb_23andme_20210107-C2 | -0.329 | 1.258 | -0.261 | 0.794 | 0.1 |
| A2 | A2_ALL_leave_UKBB_23andme_20210107-A1 | 0.509 | 6.688 | 0.076 | 0.939 | 0.027 |
| A2 | B1_ALL_leave_UKBB_23andme_20210107-A1 | -2.802 | 1.618 | -1.732 | 0.084 | 1.076 |
| A2 | A2_ALL_eur_leave_ukbb_23andme_20210107-lenientA1 | 0.9583 | 7.458 | 0.129 | 0.898 | 0.047 |
| A2 | B1_ALL_eur_leave_ukbb_23andme_20210107-lenientA1 | -14.354 | 7.148 | -2.008 | 0.046 | 1.337 |
| B1 | B2_ALL_eur_leave_ukbb_23andme_20210107-lenientA1 | -158.1227 | 79.028 | -2.001 | 0.046 | 1.337 |
| B1 | A2_ALL_leave_UKBB_23andme_20210107-B1 | -8.9912 | 5.457 | -1.648 | 0.1 | 1 |
| B1 | B1_ALL_leave_UKBB_23andme_20210107-A2 | -0.988 | 0.847 | -1.167 | 0.244 | 0.613 |
| B1 | B1_ALL_leave_UKBB_23andme_20210107-B1 | -0.0631 | 0.092 | -0.687 | 0.492 | 0.308 |
| B1 | A2_ALL_leave_UKBB_23andme_20210107-A2 | -0.1018 | 2.665 | -0.038 | 0.97 | 0.013 |
| B1 | B2_ALL_leave_UKBB_23andme_20210107-A1 | -0.2107 | 0.205 | -1.027 | 0.305 | 0.516 |
| B1 | B2_ALL_eur_leave_ukbb_23andme_20210107-B1 | -3.7563 | 4.305 | -0.872 | 0.383 | 0.417 |
| B1 | A2_ALL_eur_leave_ukbb_23andme_20210107-B2 | -34.1374 | 30.398 | -1.123 | 0.262 | 0.582 |
| B1 | B1_ALL_eur_leave_ukbb_23andme_20210107-C1 | -126.8545 | 140.725 | -0.901 | 0.368 | 0.434 |
| B1 | A2_ALL_leave_UKBB_23andme_20210107-lenientA1 | -8.9912 | 5.457 | -1.648 | 0.1 | 1 |
| B1 | A2_ALL_eur_leave_ukbb_23andme_20210107-C1 | -100.2122 | 52.362 | -1.914 | 0.056 | 1.252 |
| B1 | B1_ALL_eur_leave_ukbb_23andme_20210107-B2 | -6.437 | 15.209 | -0.423 | 0.672 | 0.173 |
| B1 | B1_ALL_leave_UKBB_23andme_20210107-lenientA1 | -0.0631 | 0.092 | -0.687 | 0.492 | 0.308 |
| B1 | C2_ALL_eur_leave_ukbb_23andme_20210107-A1 | -5.1617 | 19.35 | -0.267 | 0.79 | 0.102 |
| B1 | B2_ALL_eur_leave_ukbb_23andme_20210107-A2 | -0.899 | 2.324 | -0.387 | 0.699 | 0.156 |
| B1 | C2_ALL_leave_UKBB_23andme_20210107-C2 | -0.4776 | 0.705 | -0.677 | 0.498 | 0.303 |
| B1 | B1_ALL_eur_leave_ukbb_23andme_20210107-A1 | 2.7947 | 7.726 | 0.362 | 0.718 | 0.144 |
| B1 | A2_ALL_eur_leave_ukbb_23andme_20210107-A1 | 0.5923 | 1.342 | 0.441 | 0.659 | 0.181 |
| B1 | B1_ALL_leave_UKBB_23andme_20210107-C2 | -0.0631 | 0.092 | -0.687 | 0.492 | 0.308 |
| B1 | B2_ALL_leave_UKBB_23andme_20210107-B2 | -12.373 | 6.357 | -1.946 | 0.052 | 1.284 |
| B1 | C2_ALL_leave_UKBB_23andme_20210107-B1 | -34.2514 | 30.395 | -1.127 | 0.26 | 0.585 |
| B1 | C2_ALL_leave_UKBB_23andme_20210107-A2 | -44.9573 | 30.639 | -1.467 | 0.143 | 0.845 |
| B1 | B2_ALL_leave_UKBB_23andme_20210107-C1 | -35.4793 | 12.847 | -2.762 | 0.006 | 2.222 |
| B1 | A2_ALL_leave_UKBB_23andme_20210107-C2 | -216.3992 | 97.086 | -2.229 | 0.026 | 1.585 |
| B1 | C2_ALL_leave_UKBB_23andme_20210107-lenientA1 | -44.9573 | 30.639 | -1.467 | 0.143 | 0.845 |
| B1 | C2_ALL_eur_leave_ukbb_23andme_20210107-B2 | 100.681 | 375.806 | 0.268 | 0.789 | 0.103 |
| B1 | C2_ALL_eur_leave_ukbb_23andme_20210107-C1 | -0.4002 | 0.654 | -0.612 | 0.541 | 0.267 |
| B1 | B2_ALL_eur_leave_ukbb_23andme_20210107-C2 | -150.9253 | 75.222 | -2.006 | 0.045 | 1.347 |
| B1 | C2_ALL_eur_leave_ukbb_23andme_20210107-lenientA1 | -0.4002 | 0.654 | -0.612 | 0.541 | 0.267 |
| B1 | C2_ALL_leave_UKBB_23andme_20210107-A1 | -44.9573 | 30.639 | -1.467 | 0.143 | 0.845 |
| B1 | B2_ALL_leave_UKBB_23andme_20210107-A2 | -102.4319 | 38.771 | -2.642 | 0.008 | 2.097 |
| B1 | A2_ALL_leave_UKBB_23andme_20210107-C1 | -88.7556 | 48.905 | -1.815 | 0.07 | 1.155 |
| B1 | B1_ALL_leave_UKBB_23andme_20210107-B2 | -0.0631 | 0.092 | -0.687 | 0.492 | 0.308 |
| B1 | A2_ALL_leave_UKBB_23andme_20210107-B2 | -8.9912 | 5.457 | -1.648 | 0.1 | 1 |
| B1 | B2_ALL_leave_UKBB_23andme_20210107-B1 | -11.8147 | 6.572 | -1.798 | 0.072 | 1.143 |
| B1 | B1_ALL_leave_UKBB_23andme_20210107-C1 | -0.0631 | 0.092 | -0.687 | 0.492 | 0.308 |
| B1 | C2_ALL_eur_leave_ukbb_23andme_20210107-A2 | 9.538 | 247.934 | 0.038 | 0.969 | 0.014 |
| B1 | B2_ALL_eur_leave_ukbb_23andme_20210107-C1 | -183.7411 | 93.347 | -1.968 | 0.049 | 1.31 |
| B1 | A2_ALL_eur_leave_ukbb_23andme_20210107-C2 | -87.7743 | 49.902 | -1.759 | 0.079 | 1.102 |
| B1 | B2_ALL_eur_leave_ukbb_23andme_20210107-B2 | -3.7563 | 4.305 | -0.872 | 0.383 | 0.417 |
| B1 | C2_ALL_eur_leave_ukbb_23andme_20210107-B1 | -62.799 | 307.748 | -0.204 | 0.838 | 0.077 |
| B1 | B2_ALL_leave_UKBB_23andme_20210107-lenientA1 | -0.2107 | 0.205 | -1.027 | 0.305 | 0.516 |
| B1 | B1_ALL_eur_leave_ukbb_23andme_20210107-C2 | -126.8545 | 140.725 | -0.901 | 0.368 | 0.434 |
| B1 | B1_ALL_eur_leave_ukbb_23andme_20210107-B1 | -7.5524 | 15.169 | -0.498 | 0.619 | 0.208 |
| B1 | A2_ALL_eur_leave_ukbb_23andme_20210107-A2 | 0.5923 | 1.342 | 0.441 | 0.659 | 0.181 |
| B1 | B2_ALL_eur_leave_ukbb_23andme_20210107-A1 | -0.3108 | 0.252 | -1.232 | 0.218 | 0.662 |
| B1 | B1_ALL_eur_leave_ukbb_23andme_20210107-A2 | 2.7947 | 7.726 | 0.362 | 0.718 | 0.144 |
| B1 | A2_ALL_eur_leave_ukbb_23andme_20210107-B1 | -35.0795 | 29.907 | -1.173 | 0.241 | 0.618 |
| B1 | B2_ALL_leave_UKBB_23andme_20210107-C2 | -36.118 | 18.105 | -1.995 | 0.046 | 1.337 |
| B1 | C2_ALL_leave_UKBB_23andme_20210107-C1 | -0.4776 | 0.705 | -0.677 | 0.498 | 0.303 |
| B1 | C2_ALL_leave_UKBB_23andme_20210107-B2 | -275.3823 | 345.776 | -0.796 | 0.426 | 0.371 |
| B1 | C2_ALL_eur_leave_ukbb_23andme_20210107-C2 | -0.4002 | 0.654 | -0.612 | 0.541 | 0.267 |
| B1 | A2_ALL_leave_UKBB_23andme_20210107-A1 | -2.4123 | 3.45 | -0.699 | 0.485 | 0.314 |
| B1 | B1_ALL_leave_UKBB_23andme_20210107-A1 | -0.988 | 0.847 | -1.167 | 0.244 | 0.613 |
| B1 | A2_ALL_eur_leave_ukbb_23andme_20210107-lenientA1 | -1.6184 | 3.588 | -0.451 | 0.652 | 0.186 |
| B1 | B1_ALL_eur_leave_ukbb_23andme_20210107-lenientA1 | 3.245 | 3.554 | 0.913 | 0.361 | 0.442 |
| B2 | B2_ALL_eur_leave_ukbb_23andme_20210107-lenientA1 | -58.6434 | 64.318 | -0.912 | 0.362 | 0.441 |
| B2 | A2_ALL_leave_UKBB_23andme_20210107-B1 | -7.6973 | 4.434 | -1.736 | 0.083 | 1.081 |
| B2 | B1_ALL_leave_UKBB_23andme_20210107-A2 | -1.0612 | 0.688 | -1.543 | 0.123 | 0.91 |
| B2 | B1_ALL_leave_UKBB_23andme_20210107-B1 | -0.0416 | 0.075 | -0.556 | 0.578 | 0.238 |
| B2 | A2_ALL_leave_UKBB_23andme_20210107-A2 | -0.4421 | 2.165 | -0.204 | 0.838 | 0.077 |
| B2 | B2_ALL_leave_UKBB_23andme_20210107-A1 | -0.1228 | 0.167 | -0.736 | 0.462 | 0.335 |
| B2 | B2_ALL_eur_leave_ukbb_23andme_20210107-B1 | -4.2057 | 3.498 | -1.202 | 0.229 | 0.64 |
| B2 | A2_ALL_eur_leave_ukbb_23andme_20210107-B2 | -35.8539 | 24.692 | -1.452 | 0.147 | 0.833 |
| B2 | B1_ALL_eur_leave_ukbb_23andme_20210107-C1 | -4.4733 | 114.402 | -0.039 | 0.969 | 0.014 |
| B2 | A2_ALL_leave_UKBB_23andme_20210107-lenientA1 | -7.6973 | 4.434 | -1.736 | 0.083 | 1.081 |
| B2 | A2_ALL_eur_leave_ukbb_23andme_20210107-C1 | -75.0542 | 42.562 | -1.763 | 0.078 | 1.108 |
| B2 | B1_ALL_eur_leave_ukbb_23andme_20210107-B2 | -0.2264 | 12.36 | -0.018 | 0.985 | 0.007 |
| B2 | B1_ALL_leave_UKBB_23andme_20210107-lenientA1 | -0.0416 | 0.075 | -0.556 | 0.578 | 0.238 |
| B2 | C2_ALL_eur_leave_ukbb_23andme_20210107-A1 | 7.1784 | 15.723 | 0.457 | 0.648 | 0.188 |
| B2 | B2_ALL_eur_leave_ukbb_23andme_20210107-A2 | -0.1561 | 1.889 | -0.083 | 0.934 | 0.03 |
| B2 | C2_ALL_leave_UKBB_23andme_20210107-C2 | 0.0515 | 0.573 | 0.09 | 0.928 | 0.032 |
| B2 | B1_ALL_eur_leave_ukbb_23andme_20210107-A1 | 3.0453 | 6.278 | 0.485 | 0.628 | 0.202 |
| B2 | A2_ALL_eur_leave_ukbb_23andme_20210107-A1 | 0.2463 | 1.091 | 0.226 | 0.821 | 0.086 |
| B2 | B1_ALL_leave_UKBB_23andme_20210107-C2 | -0.0416 | 0.075 | -0.556 | 0.578 | 0.238 |
| B2 | B2_ALL_leave_UKBB_23andme_20210107-B2 | -6.2381 | 5.171 | -1.206 | 0.228 | 0.642 |
| B2 | C2_ALL_leave_UKBB_23andme_20210107-B1 | -15.8859 | 24.71 | -0.643 | 0.52 | 0.284 |
| B2 | C2_ALL_leave_UKBB_23andme_20210107-A2 | -21.8112 | 24.915 | -0.875 | 0.382 | 0.418 |
| B2 | B2_ALL_leave_UKBB_23andme_20210107-C1 | -17.63 | 10.464 | -1.685 | 0.092 | 1.036 |
| B2 | A2_ALL_leave_UKBB_23andme_20210107-C2 | -106.0528 | 79.015 | -1.342 | 0.18 | 0.745 |
| B2 | C2_ALL_leave_UKBB_23andme_20210107-lenientA1 | -21.8112 | 24.915 | -0.875 | 0.382 | 0.418 |
| B2 | C2_ALL_eur_leave_ukbb_23andme_20210107-B2 | 450.5185 | 305.083 | 1.477 | 0.14 | 0.854 |
| B2 | C2_ALL_eur_leave_ukbb_23andme_20210107-C1 | 0.0759 | 0.531 | 0.143 | 0.886 | 0.053 |
| B2 | B2_ALL_eur_leave_ukbb_23andme_20210107-C2 | -52.8869 | 61.224 | -0.864 | 0.388 | 0.411 |
| B2 | C2_ALL_eur_leave_ukbb_23andme_20210107-lenientA1 | 0.0759 | 0.531 | 0.143 | 0.886 | 0.053 |
| B2 | C2_ALL_leave_UKBB_23andme_20210107-A1 | -21.8112 | 24.915 | -0.875 | 0.382 | 0.418 |
| B2 | B2_ALL_leave_UKBB_23andme_20210107-A2 | -46.7219 | 31.578 | -1.48 | 0.139 | 0.857 |
| B2 | A2_ALL_leave_UKBB_23andme_20210107-C1 | -44.4317 | 39.781 | -1.117 | 0.264 | 0.578 |
| B2 | B1_ALL_leave_UKBB_23andme_20210107-B2 | -0.0416 | 0.075 | -0.556 | 0.578 | 0.238 |
| B2 | A2_ALL_leave_UKBB_23andme_20210107-B2 | -7.6973 | 4.434 | -1.736 | 0.083 | 1.081 |
| B2 | B2_ALL_leave_UKBB_23andme_20210107-B1 | -6.1872 | 5.345 | -1.158 | 0.247 | 0.607 |
| B2 | B1_ALL_leave_UKBB_23andme_20210107-C1 | -0.0416 | 0.075 | -0.556 | 0.578 | 0.238 |
| B2 | C2_ALL_eur_leave_ukbb_23andme_20210107-A2 | 224.1193 | 201.36 | 1.113 | 0.266 | 0.575 |
| B2 | B2_ALL_eur_leave_ukbb_23andme_20210107-C1 | -62.7853 | 75.973 | -0.826 | 0.409 | 0.388 |
| B2 | A2_ALL_eur_leave_ukbb_23andme_20210107-C2 | -68.5703 | 40.556 | -1.691 | 0.091 | 1.041 |
| B2 | B2_ALL_eur_leave_ukbb_23andme_20210107-B2 | -4.2057 | 3.498 | -1.202 | 0.229 | 0.64 |
| B2 | C2_ALL_eur_leave_ukbb_23andme_20210107-B1 | 265.5187 | 249.955 | 1.062 | 0.288 | 0.541 |
| B2 | B2_ALL_leave_UKBB_23andme_20210107-lenientA1 | -0.1228 | 0.167 | -0.736 | 0.462 | 0.335 |
| B2 | B1_ALL_eur_leave_ukbb_23andme_20210107-C2 | -4.4733 | 114.402 | -0.039 | 0.969 | 0.014 |
| B2 | B1_ALL_eur_leave_ukbb_23andme_20210107-B1 | -1.5611 | 12.328 | -0.127 | 0.899 | 0.046 |
| B2 | A2_ALL_eur_leave_ukbb_23andme_20210107-A2 | 0.2463 | 1.091 | 0.226 | 0.821 | 0.086 |
| B2 | B2_ALL_eur_leave_ukbb_23andme_20210107-A1 | -0.1529 | 0.205 | -0.746 | 0.456 | 0.341 |
| B2 | B1_ALL_eur_leave_ukbb_23andme_20210107-A2 | 3.0453 | 6.278 | 0.485 | 0.628 | 0.202 |
| B2 | A2_ALL_eur_leave_ukbb_23andme_20210107-B1 | -35.3754 | 24.295 | -1.456 | 0.146 | 0.836 |
| B2 | B2_ALL_leave_UKBB_23andme_20210107-C2 | -16.8214 | 14.732 | -1.142 | 0.254 | 0.595 |
| B2 | C2_ALL_leave_UKBB_23andme_20210107-C1 | 0.0515 | 0.573 | 0.09 | 0.928 | 0.032 |
| B2 | C2_ALL_leave_UKBB_23andme_20210107-B2 | 93.8073 | 281.058 | 0.334 | 0.739 | 0.131 |
| B2 | C2_ALL_eur_leave_ukbb_23andme_20210107-C2 | 0.0759 | 0.531 | 0.143 | 0.886 | 0.053 |
| B2 | A2_ALL_leave_UKBB_23andme_20210107-A1 | -3.1191 | 2.803 | -1.113 | 0.266 | 0.575 |
| B2 | B1_ALL_leave_UKBB_23andme_20210107-A1 | -1.0612 | 0.688 | -1.543 | 0.123 | 0.91 |
| B2 | A2_ALL_eur_leave_ukbb_23andme_20210107-lenientA1 | -1.4274 | 2.916 | -0.49 | 0.625 | 0.204 |
| B2 | B1_ALL_eur_leave_ukbb_23andme_20210107-lenientA1 | 0.1906 | 2.889 | 0.066 | 0.947 | 0.024 |
| C1 | B2_ALL_eur_leave_ukbb_23andme_20210107-lenientA1 | -0.5738 | 13.933 | -0.041 | 0.967 | 0.015 |
| C1 | A2_ALL_leave_UKBB_23andme_20210107-B1 | -0.0104 | 0.949 | -0.011 | 0.991 | 0.004 |
| C1 | B1_ALL_leave_UKBB_23andme_20210107-A2 | -0.1075 | 0.147 | -0.731 | 0.465 | 0.333 |
| C1 | B1_ALL_leave_UKBB_23andme_20210107-B1 | -0.0276 | 0.017 | -1.603 | 0.109 | 0.963 |
| C1 | A2_ALL_leave_UKBB_23andme_20210107-A2 | -0.4117 | 0.459 | -0.896 | 0.37 | 0.432 |
| C1 | B2_ALL_leave_UKBB_23andme_20210107-A1 | -0.0453 | 0.037 | -1.208 | 0.227 | 0.644 |
| C1 | B2_ALL_eur_leave_ukbb_23andme_20210107-B1 | -2.237 | 0.75 | -2.982 | 0.003 | 2.523 |
| C1 | A2_ALL_eur_leave_ukbb_23andme_20210107-B2 | 2.6243 | 5.287 | 0.496 | 0.62 | 0.208 |
| C1 | B1_ALL_eur_leave_ukbb_23andme_20210107-C1 | 24.1584 | 23.593 | 1.024 | 0.306 | 0.514 |
| C1 | A2_ALL_leave_UKBB_23andme_20210107-lenientA1 | -0.0104 | 0.949 | -0.011 | 0.991 | 0.004 |
| C1 | A2_ALL_eur_leave_ukbb_23andme_20210107-C1 | 6.9123 | 9.063 | 0.763 | 0.446 | 0.351 |
| C1 | B1_ALL_eur_leave_ukbb_23andme_20210107-B2 | 1.7291 | 2.644 | 0.654 | 0.513 | 0.29 |
| C1 | B1_ALL_leave_UKBB_23andme_20210107-lenientA1 | -0.0276 | 0.017 | -1.603 | 0.109 | 0.963 |
| C1 | C2_ALL_eur_leave_ukbb_23andme_20210107-A1 | -4.6841 | 3.362 | -1.393 | 0.164 | 0.785 |
| C1 | B2_ALL_eur_leave_ukbb_23andme_20210107-A2 | -0.4974 | 0.419 | -1.188 | 0.235 | 0.629 |
| C1 | C2_ALL_leave_UKBB_23andme_20210107-C2 | 0.1102 | 0.126 | 0.874 | 0.382 | 0.418 |
| C1 | B1_ALL_eur_leave_ukbb_23andme_20210107-A1 | 0.3805 | 1.316 | 0.289 | 0.773 | 0.112 |
| C1 | A2_ALL_eur_leave_ukbb_23andme_20210107-A1 | -0.2111 | 0.226 | -0.933 | 0.351 | 0.455 |
| C1 | B1_ALL_leave_UKBB_23andme_20210107-C2 | -0.0276 | 0.017 | -1.603 | 0.109 | 0.963 |
| C1 | B2_ALL_leave_UKBB_23andme_20210107-B2 | -2.1053 | 1.096 | -1.921 | 0.055 | 1.26 |
| C1 | C2_ALL_leave_UKBB_23andme_20210107-B1 | 3.6603 | 5.35 | 0.684 | 0.494 | 0.306 |
| C1 | C2_ALL_leave_UKBB_23andme_20210107-A2 | 4.8813 | 5.401 | 0.904 | 0.366 | 0.437 |
| C1 | B2_ALL_leave_UKBB_23andme_20210107-C1 | -1.1867 | 2.203 | -0.539 | 0.59 | 0.229 |
| C1 | A2_ALL_leave_UKBB_23andme_20210107-C2 | -12.7257 | 17.026 | -0.747 | 0.455 | 0.342 |
| C1 | C2_ALL_leave_UKBB_23andme_20210107-lenientA1 | 4.8813 | 5.401 | 0.904 | 0.366 | 0.437 |
| C1 | C2_ALL_eur_leave_ukbb_23andme_20210107-B2 | 50.5288 | 65.29 | 0.774 | 0.439 | 0.358 |
| C1 | C2_ALL_eur_leave_ukbb_23andme_20210107-C1 | 0.113 | 0.116 | 0.973 | 0.331 | 0.48 |
| C1 | B2_ALL_eur_leave_ukbb_23andme_20210107-C2 | 0.5594 | 13.289 | 0.042 | 0.966 | 0.015 |
| C1 | C2_ALL_eur_leave_ukbb_23andme_20210107-lenientA1 | 0.113 | 0.116 | 0.973 | 0.331 | 0.48 |
| C1 | C2_ALL_leave_UKBB_23andme_20210107-A1 | 4.8813 | 5.401 | 0.904 | 0.366 | 0.437 |
| C1 | B2_ALL_leave_UKBB_23andme_20210107-A2 | -0.684 | 6.719 | -0.102 | 0.919 | 0.037 |
| C1 | A2_ALL_leave_UKBB_23andme_20210107-C1 | -6.5647 | 8.344 | -0.787 | 0.431 | 0.366 |
| C1 | B1_ALL_leave_UKBB_23andme_20210107-B2 | -0.0276 | 0.017 | -1.603 | 0.109 | 0.963 |
| C1 | A2_ALL_leave_UKBB_23andme_20210107-B2 | -0.0104 | 0.949 | -0.011 | 0.991 | 0.004 |
| C1 | B2_ALL_leave_UKBB_23andme_20210107-B1 | -1.8221 | 1.131 | -1.612 | 0.107 | 0.971 |
| C1 | B1_ALL_leave_UKBB_23andme_20210107-C1 | -0.0276 | 0.017 | -1.603 | 0.109 | 0.963 |
| C1 | C2_ALL_eur_leave_ukbb_23andme_20210107-A2 | 42.2299 | 43.136 | 0.979 | 0.328 | 0.484 |
| C1 | B2_ALL_eur_leave_ukbb_23andme_20210107-C1 | -1.8959 | 16.418 | -0.115 | 0.908 | 0.042 |
| C1 | A2_ALL_eur_leave_ukbb_23andme_20210107-C2 | 5.2955 | 8.692 | 0.609 | 0.542 | 0.266 |
| C1 | B2_ALL_eur_leave_ukbb_23andme_20210107-B2 | -2.237 | 0.75 | -2.982 | 0.003 | 2.523 |
| C1 | C2_ALL_eur_leave_ukbb_23andme_20210107-B1 | 50.8489 | 53.804 | 0.945 | 0.345 | 0.462 |
| C1 | B2_ALL_leave_UKBB_23andme_20210107-lenientA1 | -0.0453 | 0.037 | -1.208 | 0.227 | 0.644 |
| C1 | B1_ALL_eur_leave_ukbb_23andme_20210107-C2 | 24.1584 | 23.593 | 1.024 | 0.306 | 0.514 |
| C1 | B1_ALL_eur_leave_ukbb_23andme_20210107-B1 | 1.6033 | 2.64 | 0.607 | 0.544 | 0.264 |
| C1 | A2_ALL_eur_leave_ukbb_23andme_20210107-A2 | -0.2111 | 0.226 | -0.933 | 0.351 | 0.455 |
| C1 | B2_ALL_eur_leave_ukbb_23andme_20210107-A1 | -0.0426 | 0.044 | -0.959 | 0.338 | 0.471 |
| C1 | B1_ALL_eur_leave_ukbb_23andme_20210107-A2 | 0.3805 | 1.316 | 0.289 | 0.773 | 0.112 |
| C1 | A2_ALL_eur_leave_ukbb_23andme_20210107-B1 | 1.6336 | 5.228 | 0.312 | 0.755 | 0.122 |
| C1 | B2_ALL_leave_UKBB_23andme_20210107-C2 | -2.1169 | 3.035 | -0.697 | 0.486 | 0.313 |
| C1 | C2_ALL_leave_UKBB_23andme_20210107-C1 | 0.1102 | 0.126 | 0.874 | 0.382 | 0.418 |
| C1 | C2_ALL_leave_UKBB_23andme_20210107-B2 | 78.1845 | 59.431 | 1.316 | 0.188 | 0.726 |
| C1 | C2_ALL_eur_leave_ukbb_23andme_20210107-C2 | 0.113 | 0.116 | 0.973 | 0.331 | 0.48 |
| C1 | A2_ALL_leave_UKBB_23andme_20210107-A1 | -0.3564 | 0.585 | -0.61 | 0.542 | 0.266 |
| C1 | B1_ALL_leave_UKBB_23andme_20210107-A1 | -0.1075 | 0.147 | -0.731 | 0.465 | 0.333 |
| C1 | A2_ALL_eur_leave_ukbb_23andme_20210107-lenientA1 | -0.6554 | 0.61 | -1.074 | 0.283 | 0.548 |
| C1 | B1_ALL_eur_leave_ukbb_23andme_20210107-lenientA1 | 0.0752 | 0.614 | 0.122 | 0.903 | 0.044 |
| C2 | B2_ALL_eur_leave_ukbb_23andme_20210107-lenientA1 | 13.2553 | 12.014 | 1.103 | 0.27 | 0.569 |
| C2 | A2_ALL_leave_UKBB_23andme_20210107-B1 | 0.5093 | 0.818 | 0.622 | 0.534 | 0.272 |
| C2 | B1_ALL_leave_UKBB_23andme_20210107-A2 | -0.1337 | 0.127 | -1.054 | 0.292 | 0.535 |
| C2 | B1_ALL_leave_UKBB_23andme_20210107-B1 | -0.0253 | 0.015 | -1.71 | 0.087 | 1.06 |
| C2 | A2_ALL_leave_UKBB_23andme_20210107-A2 | -0.2057 | 0.396 | -0.519 | 0.604 | 0.219 |
| C2 | B2_ALL_leave_UKBB_23andme_20210107-A1 | -0.0402 | 0.032 | -1.244 | 0.213 | 0.672 |
| C2 | B2_ALL_eur_leave_ukbb_23andme_20210107-B1 | -1.8018 | 0.647 | -2.785 | 0.005 | 2.301 |
| C2 | A2_ALL_eur_leave_ukbb_23andme_20210107-B2 | 7.8953 | 4.558 | 1.732 | 0.083 | 1.081 |
| C2 | B1_ALL_eur_leave_ukbb_23andme_20210107-C1 | 24.9554 | 20.343 | 1.227 | 0.22 | 0.658 |
| C2 | A2_ALL_leave_UKBB_23andme_20210107-lenientA1 | 0.5093 | 0.818 | 0.622 | 0.534 | 0.272 |
| C2 | A2_ALL_eur_leave_ukbb_23andme_20210107-C1 | 12.2394 | 7.814 | 1.566 | 0.117 | 0.932 |
| C2 | B1_ALL_eur_leave_ukbb_23andme_20210107-B2 | 2.3858 | 2.28 | 1.046 | 0.295 | 0.53 |
| C2 | B1_ALL_leave_UKBB_23andme_20210107-lenientA1 | -0.0253 | 0.015 | -1.71 | 0.087 | 1.06 |
| C2 | C2_ALL_eur_leave_ukbb_23andme_20210107-A1 | -2.5145 | 2.899 | -0.867 | 0.386 | 0.413 |
| C2 | B2_ALL_eur_leave_ukbb_23andme_20210107-A2 | -0.2003 | 0.361 | -0.555 | 0.579 | 0.237 |
| C2 | C2_ALL_leave_UKBB_23andme_20210107-C2 | 0.0584 | 0.109 | 0.537 | 0.591 | 0.228 |
| C2 | B1_ALL_eur_leave_ukbb_23andme_20210107-A1 | 0.1088 | 1.135 | 0.096 | 0.924 | 0.034 |
| C2 | A2_ALL_eur_leave_ukbb_23andme_20210107-A1 | -0.1719 | 0.195 | -0.881 | 0.379 | 0.421 |
| C2 | B1_ALL_leave_UKBB_23andme_20210107-C2 | -0.0253 | 0.015 | -1.71 | 0.087 | 1.06 |
| C2 | B2_ALL_leave_UKBB_23andme_20210107-B2 | -1.5171 | 0.945 | -1.605 | 0.108 | 0.967 |
| C2 | C2_ALL_leave_UKBB_23andme_20210107-B1 | 6.1895 | 4.613 | 1.342 | 0.18 | 0.745 |
| C2 | C2_ALL_leave_UKBB_23andme_20210107-A2 | 6.8719 | 4.657 | 1.476 | 0.14 | 0.854 |
| C2 | B2_ALL_leave_UKBB_23andme_20210107-C1 | 1.1131 | 1.899 | 0.586 | 0.558 | 0.253 |
| C2 | A2_ALL_leave_UKBB_23andme_20210107-C2 | 4.997 | 14.681 | 0.34 | 0.734 | 0.134 |
| C2 | C2_ALL_leave_UKBB_23andme_20210107-lenientA1 | 6.8719 | 4.657 | 1.476 | 0.14 | 0.854 |
| C2 | C2_ALL_eur_leave_ukbb_23andme_20210107-B2 | 132.7027 | 56.283 | 2.358 | 0.018 | 1.745 |
| C2 | C2_ALL_eur_leave_ukbb_23andme_20210107-C1 | 0.0616 | 0.1 | 0.615 | 0.539 | 0.268 |
| C2 | B2_ALL_eur_leave_ukbb_23andme_20210107-C2 | 13.2747 | 11.458 | 1.159 | 0.247 | 0.607 |
| C2 | C2_ALL_eur_leave_ukbb_23andme_20210107-lenientA1 | 0.0616 | 0.1 | 0.615 | 0.539 | 0.268 |
| C2 | C2_ALL_leave_UKBB_23andme_20210107-A1 | 6.8719 | 4.657 | 1.476 | 0.14 | 0.854 |
| C2 | B2_ALL_leave_UKBB_23andme_20210107-A2 | 7.3842 | 5.793 | 1.275 | 0.202 | 0.695 |
| C2 | A2_ALL_leave_UKBB_23andme_20210107-C1 | 2.9664 | 7.195 | 0.412 | 0.68 | 0.167 |
| C2 | B1_ALL_leave_UKBB_23andme_20210107-B2 | -0.0253 | 0.015 | -1.71 | 0.087 | 1.06 |
| C2 | A2_ALL_leave_UKBB_23andme_20210107-B2 | 0.5093 | 0.818 | 0.622 | 0.534 | 0.272 |
| C2 | B2_ALL_leave_UKBB_23andme_20210107-B1 | -1.373 | 0.975 | -1.408 | 0.159 | 0.799 |
| C2 | B1_ALL_leave_UKBB_23andme_20210107-C1 | -0.0253 | 0.015 | -1.71 | 0.087 | 1.06 |
| C2 | C2_ALL_eur_leave_ukbb_23andme_20210107-A2 | 88.0158 | 37.186 | 2.367 | 0.018 | 1.745 |
| C2 | B2_ALL_eur_leave_ukbb_23andme_20210107-C1 | 15.8988 | 14.156 | 1.123 | 0.261 | 0.583 |
| C2 | A2_ALL_eur_leave_ukbb_23andme_20210107-C2 | 11.1031 | 7.494 | 1.482 | 0.138 | 0.86 |
| C2 | B2_ALL_eur_leave_ukbb_23andme_20210107-B2 | -1.8018 | 0.647 | -2.785 | 0.005 | 2.301 |
| C2 | C2_ALL_eur_leave_ukbb_23andme_20210107-B1 | 112.7114 | 46.382 | 2.43 | 0.015 | 1.824 |
| C2 | B2_ALL_leave_UKBB_23andme_20210107-lenientA1 | -0.0402 | 0.032 | -1.244 | 0.213 | 0.672 |
| C2 | B1_ALL_eur_leave_ukbb_23andme_20210107-C2 | 24.9554 | 20.343 | 1.227 | 0.22 | 0.658 |
| C2 | B1_ALL_eur_leave_ukbb_23andme_20210107-B1 | 2.2697 | 2.276 | 0.997 | 0.319 | 0.496 |
| C2 | A2_ALL_eur_leave_ukbb_23andme_20210107-A2 | -0.1719 | 0.195 | -0.881 | 0.379 | 0.421 |
| C2 | B2_ALL_eur_leave_ukbb_23andme_20210107-A1 | -0.0363 | 0.038 | -0.949 | 0.343 | 0.465 |
| C2 | B1_ALL_eur_leave_ukbb_23andme_20210107-A2 | 0.1088 | 1.135 | 0.096 | 0.924 | 0.034 |
| C2 | A2_ALL_eur_leave_ukbb_23andme_20210107-B1 | 7.1232 | 4.507 | 1.58 | 0.114 | 0.943 |
| C2 | B2_ALL_leave_UKBB_23andme_20210107-C2 | 0.5957 | 2.617 | 0.228 | 0.82 | 0.086 |
| C2 | C2_ALL_leave_UKBB_23andme_20210107-C1 | 0.0584 | 0.109 | 0.537 | 0.591 | 0.228 |
| C2 | C2_ALL_leave_UKBB_23andme_20210107-B2 | 117.1853 | 51.236 | 2.287 | 0.022 | 1.658 |
| C2 | C2_ALL_eur_leave_ukbb_23andme_20210107-C2 | 0.0616 | 0.1 | 0.615 | 0.539 | 0.268 |
| C2 | A2_ALL_leave_UKBB_23andme_20210107-A1 | -0.2445 | 0.504 | -0.485 | 0.628 | 0.202 |
| C2 | B1_ALL_leave_UKBB_23andme_20210107-A1 | -0.1337 | 0.127 | -1.054 | 0.292 | 0.535 |
| C2 | A2_ALL_eur_leave_ukbb_23andme_20210107-lenientA1 | -0.4981 | 0.526 | -0.946 | 0.344 | 0.463 |
| C2 | B1_ALL_eur_leave_ukbb_23andme_20210107-lenientA1 | -0.1997 | 0.53 | -0.377 | 0.706 | 0.151 |
| lenient_A1 | B2_ALL_eur_leave_ukbb_23andme_20210107-lenientA1 | 47.8442 | 127.883 | 0.374 | 0.708 | 0.15 |
| lenient_A1 | A2_ALL_leave_UKBB_23andme_20210107-B1 | -18.2775 | 8.276 | -2.208 | 0.028 | 1.553 |
| lenient_A1 | B1_ALL_leave_UKBB_23andme_20210107-A2 | -2.2847 | 1.267 | -1.803 | 0.072 | 1.143 |
| lenient_A1 | B1_ALL_leave_UKBB_23andme_20210107-B1 | -0.1426 | 0.142 | -1.007 | 0.315 | 0.502 |
| lenient_A1 | A2_ALL_leave_UKBB_23andme_20210107-A2 | -4.2227 | 3.906 | -1.081 | 0.28 | 0.553 |
| lenient_A1 | B2_ALL_leave_UKBB_23andme_20210107-A1 | -0.5841 | 0.317 | -1.843 | 0.066 | 1.18 |
| lenient_A1 | B2_ALL_eur_leave_ukbb_23andme_20210107-B1 | -3.0114 | 6.983 | -0.431 | 0.666 | 0.177 |
| lenient_A1 | A2_ALL_eur_leave_ukbb_23andme_20210107-B2 | -55.0334 | 48.215 | -1.141 | 0.254 | 0.595 |
| lenient_A1 | B1_ALL_eur_leave_ukbb_23andme_20210107-C1 | -41.8185 | 224.817 | -0.186 | 0.853 | 0.069 |
| lenient_A1 | A2_ALL_leave_UKBB_23andme_20210107-lenientA1 | -18.2775 | 8.276 | -2.208 | 0.028 | 1.553 |
| lenient_A1 | A2_ALL_eur_leave_ukbb_23andme_20210107-C1 | -105.0246 | 80.834 | -1.299 | 0.195 | 0.71 |
| lenient_A1 | B1_ALL_eur_leave_ukbb_23andme_20210107-B2 | -0.3556 | 23.27 | -0.015 | 0.988 | 0.005 |
| lenient_A1 | B1_ALL_leave_UKBB_23andme_20210107-lenientA1 | -0.1426 | 0.142 | -1.007 | 0.315 | 0.502 |
| lenient_A1 | C2_ALL_eur_leave_ukbb_23andme_20210107-A1 | -4.4042 | 31.922 | -0.138 | 0.89 | 0.051 |
| lenient_A1 | B2_ALL_eur_leave_ukbb_23andme_20210107-A2 | 1.5182 | 3.723 | 0.408 | 0.684 | 0.165 |
| lenient_A1 | C2_ALL_leave_UKBB_23andme_20210107-C2 | -1.1126 | 1.062 | -1.048 | 0.295 | 0.53 |
| lenient_A1 | B1_ALL_eur_leave_ukbb_23andme_20210107-A1 | 5.3173 | 11.968 | 0.444 | 0.657 | 0.182 |
| lenient_A1 | A2_ALL_eur_leave_ukbb_23andme_20210107-A1 | -2.7407 | 2.089 | -1.312 | 0.19 | 0.721 |
| lenient_A1 | B1_ALL_leave_UKBB_23andme_20210107-C2 | -0.1426 | 0.142 | -1.007 | 0.315 | 0.502 |
| lenient_A1 | B2_ALL_leave_UKBB_23andme_20210107-B2 | -12.8678 | 9.702 | -1.326 | 0.185 | 0.733 |
| lenient_A1 | C2_ALL_leave_UKBB_23andme_20210107-B1 | 59.0967 | 48.502 | 1.218 | 0.224 | 0.65 |
| lenient_A1 | C2_ALL_leave_UKBB_23andme_20210107-A2 | 48.8199 | 48.756 | 1.001 | 0.317 | 0.499 |
| lenient_A1 | B2_ALL_leave_UKBB_23andme_20210107-C1 | -32.3545 | 19.791 | -1.635 | 0.103 | 0.987 |
| lenient_A1 | A2_ALL_leave_UKBB_23andme_20210107-C2 | -99.7971 | 153.296 | -0.651 | 0.515 | 0.288 |
| lenient_A1 | C2_ALL_leave_UKBB_23andme_20210107-lenientA1 | 48.8199 | 48.756 | 1.001 | 0.317 | 0.499 |
| lenient_A1 | C2_ALL_eur_leave_ukbb_23andme_20210107-B2 | 667.3544 | 598.392 | 1.115 | 0.265 | 0.577 |
| lenient_A1 | C2_ALL_eur_leave_ukbb_23andme_20210107-C1 | -0.995 | 0.981 | -1.015 | 0.311 | 0.507 |
| lenient_A1 | B2_ALL_eur_leave_ukbb_23andme_20210107-C2 | 44.9686 | 121.456 | 0.37 | 0.711 | 0.148 |
| lenient_A1 | C2_ALL_eur_leave_ukbb_23andme_20210107-lenientA1 | -0.995 | 0.981 | -1.015 | 0.311 | 0.507 |
| lenient_A1 | C2_ALL_leave_UKBB_23andme_20210107-A1 | 48.8199 | 48.756 | 1.001 | 0.317 | 0.499 |
| lenient_A1 | B2_ALL_leave_UKBB_23andme_20210107-A2 | -75.0688 | 60.06 | -1.25 | 0.212 | 0.674 |
| lenient_A1 | A2_ALL_leave_UKBB_23andme_20210107-C1 | -54.1598 | 74.788 | -0.724 | 0.469 | 0.329 |
| lenient_A1 | B1_ALL_leave_UKBB_23andme_20210107-B2 | -0.1426 | 0.142 | -1.007 | 0.315 | 0.502 |
| lenient_A1 | A2_ALL_leave_UKBB_23andme_20210107-B2 | -18.2775 | 8.276 | -2.208 | 0.028 | 1.553 |
| lenient_A1 | B2_ALL_leave_UKBB_23andme_20210107-B1 | -14.086 | 9.932 | -1.418 | 0.157 | 0.804 |
| lenient_A1 | B1_ALL_leave_UKBB_23andme_20210107-C1 | -0.1426 | 0.142 | -1.007 | 0.315 | 0.502 |
| lenient_A1 | C2_ALL_eur_leave_ukbb_23andme_20210107-A2 | 395.4019 | 390.259 | 1.013 | 0.312 | 0.506 |
| lenient_A1 | B2_ALL_eur_leave_ukbb_23andme_20210107-C1 | 24.2582 | 152.042 | 0.16 | 0.873 | 0.059 |
| lenient_A1 | A2_ALL_eur_leave_ukbb_23andme_20210107-C2 | -84.796 | 77.286 | -1.097 | 0.273 | 0.564 |
| lenient_A1 | B2_ALL_eur_leave_ukbb_23andme_20210107-B2 | -3.0114 | 6.983 | -0.431 | 0.666 | 0.177 |
| lenient_A1 | C2_ALL_eur_leave_ukbb_23andme_20210107-B1 | 402.7431 | 492.109 | 0.818 | 0.414 | 0.383 |
| lenient_A1 | B2_ALL_leave_UKBB_23andme_20210107-lenientA1 | -0.5841 | 0.317 | -1.843 | 0.066 | 1.18 |
| lenient_A1 | B1_ALL_eur_leave_ukbb_23andme_20210107-C2 | -41.8185 | 224.817 | -0.186 | 0.853 | 0.069 |
| lenient_A1 | B1_ALL_eur_leave_ukbb_23andme_20210107-B1 | -0.2249 | 23.217 | -0.01 | 0.992 | 0.003 |
| lenient_A1 | A2_ALL_eur_leave_ukbb_23andme_20210107-A2 | -2.7407 | 2.089 | -1.312 | 0.19 | 0.721 |
| lenient_A1 | B2_ALL_eur_leave_ukbb_23andme_20210107-A1 | -0.8076 | 0.386 | -2.09 | 0.037 | 1.432 |
| lenient_A1 | B1_ALL_eur_leave_ukbb_23andme_20210107-A2 | 5.3173 | 11.968 | 0.444 | 0.657 | 0.182 |
| lenient_A1 | A2_ALL_eur_leave_ukbb_23andme_20210107-B1 | -55.1764 | 47.372 | -1.165 | 0.245 | 0.611 |
| lenient_A1 | B2_ALL_leave_UKBB_23andme_20210107-C2 | -23.7189 | 27.487 | -0.863 | 0.389 | 0.41 |
| lenient_A1 | C2_ALL_leave_UKBB_23andme_20210107-C1 | -1.1126 | 1.062 | -1.048 | 0.295 | 0.53 |
| lenient_A1 | C2_ALL_leave_UKBB_23andme_20210107-B2 | 54.8141 | 560.57 | 0.098 | 0.922 | 0.035 |
| lenient_A1 | C2_ALL_eur_leave_ukbb_23andme_20210107-C2 | -0.995 | 0.981 | -1.015 | 0.311 | 0.507 |
| lenient_A1 | A2_ALL_leave_UKBB_23andme_20210107-A1 | -6.4433 | 5.193 | -1.241 | 0.215 | 0.668 |
| lenient_A1 | B1_ALL_leave_UKBB_23andme_20210107-A1 | -2.2847 | 1.267 | -1.803 | 0.072 | 1.143 |
| lenient_A1 | A2_ALL_eur_leave_ukbb_23andme_20210107-lenientA1 | -3.074 | 5.65 | -0.544 | 0.587 | 0.231 |
| lenient_A1 | B1_ALL_eur_leave_ukbb_23andme_20210107-lenientA1 | -8.34 | 5.314 | -1.57 | 0.117 | 0.932 |

**Supplementary Table S3.** Linear regression results for the associations between 56 top polygenic scores and disease risk prediction based on medical comorbidities, among cases (not controls).


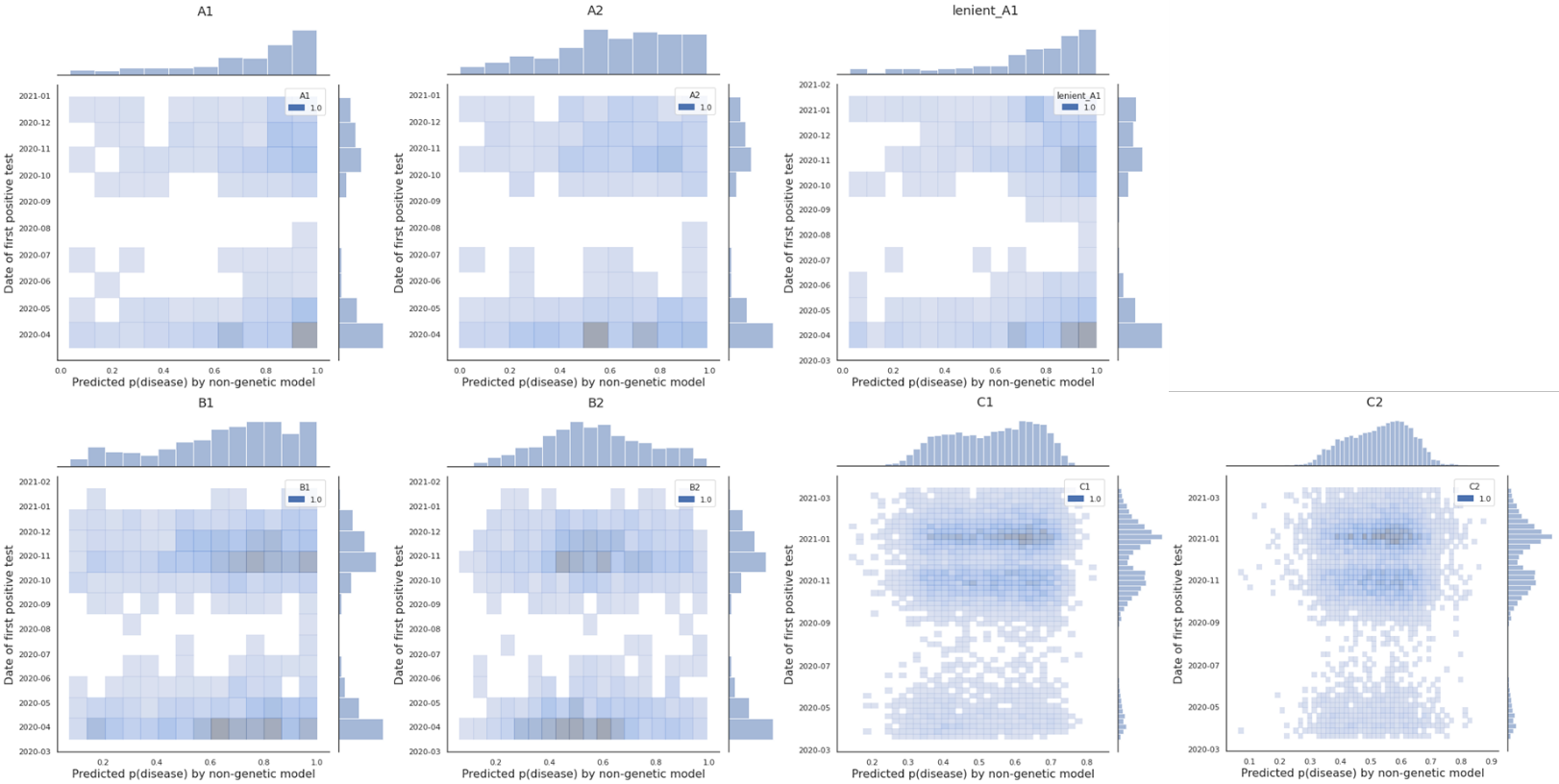


**Supplementary Figure S2.** Relationship between predicted probability of disease (modeled using medical risk factors) and reported COVID-19 date of onset in the UK Biobank. Dates were entered as numeric timestamps from the first positive COVID-19 test result.


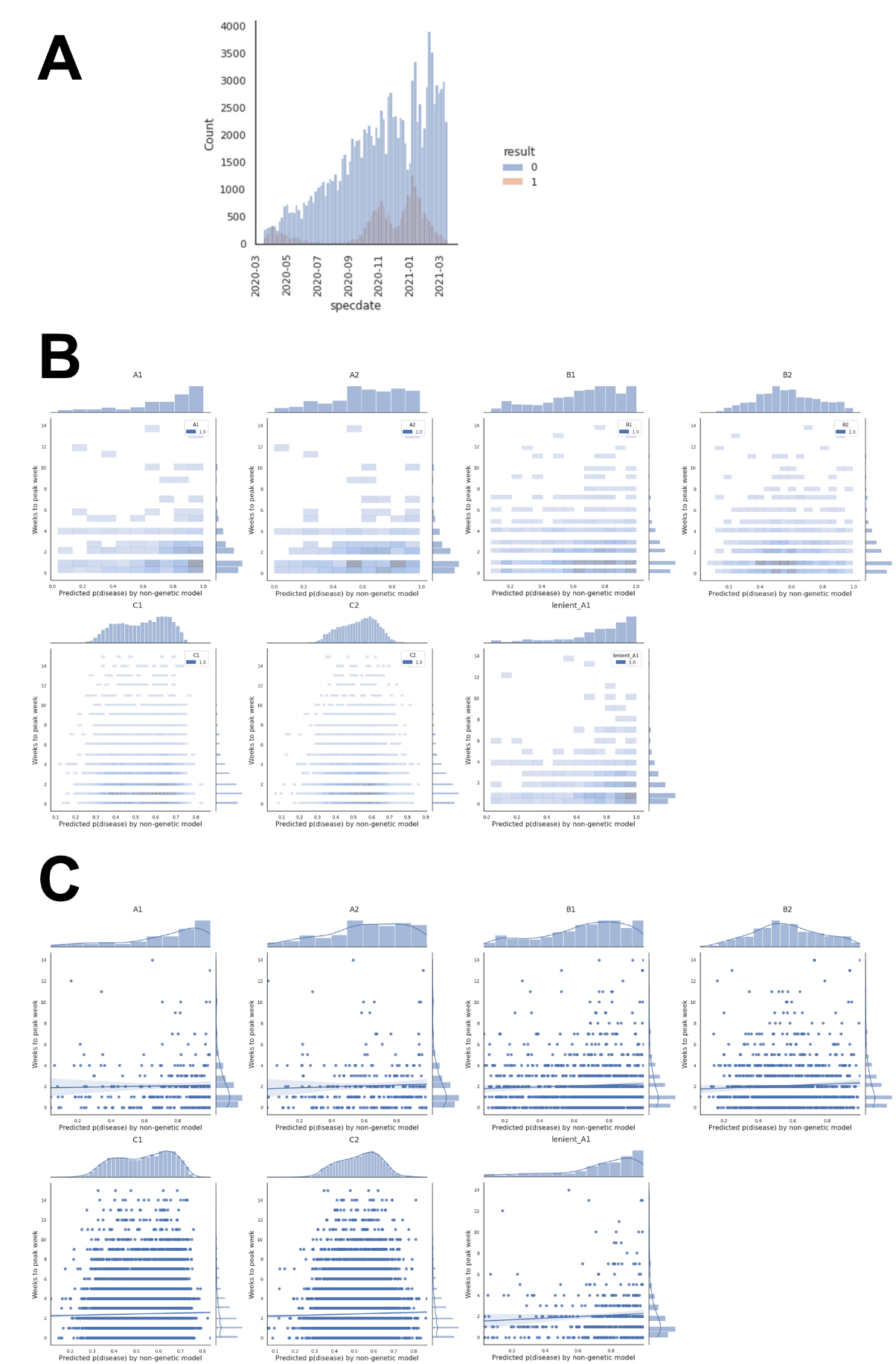


**Supplementary Figure S3.** Relationship between predicted probability of disease (by medical risk factors) and time to nearest population peak in positive COVID-19 test results. A: Distribution of positive and negative test results displays three modes around April 2020, November 2020 and January 2021; B: distribution of model predictions and time (in weeks) to any of the top three weeks in the population; C: regression line fit across data on B.

| **Trait** | **Coef.** | **Std. Err.** | **t** | **P>\|t\|** |
| --- | --- | --- | --- | --- |
| *A1* | 0.002992 | 0.006452 | 0.463686 | 0.643238 |
| *A2* | 0.004798 | 0.006353 | 0.75524 | 0.450749 |
| *B1* | 0.005404 | 0.003295 | 1.63993 | 0.101322 |
| *B2* | 0.004691 | 0.002679 | 1.75144 | 0.080163 |
| *C1* | 0.001248 | 0.000529 | 2.35879 | 0.018355 |
| *C2* | 0.001034 | 0.000456 | 2.26613 | 0.023466 |
| *lenient_A1* | 0.007818 | 0.00491 | 1.59235 | 0.112018 |

**Supplementary Table S4.** Linear regression test results for the associations between predicted probability of disease and time from COVID-19 onset to population peak in COVID-19 positive cases.


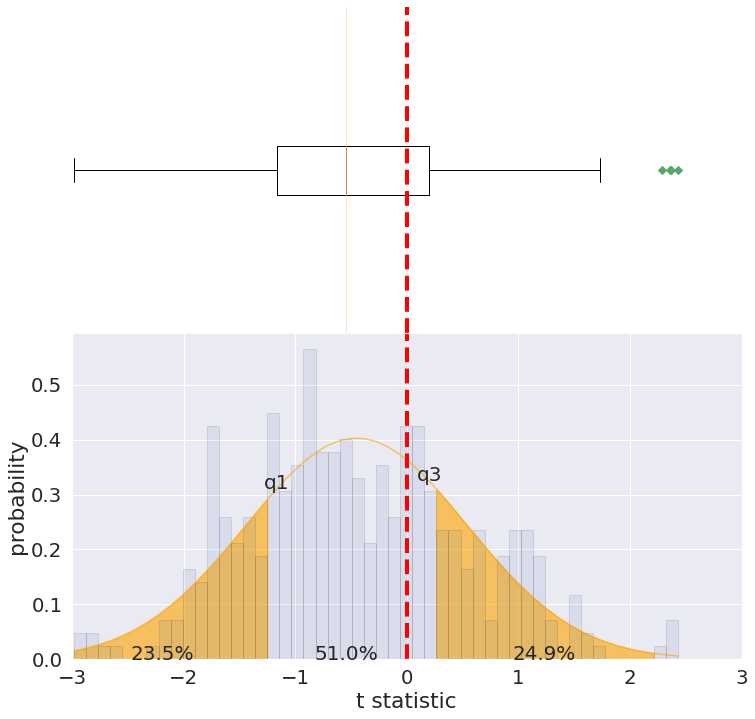


**Supplementary Figure S4.** Distribution of *t* statistics from linear regression results displayed on Supplementary Table S3 (associations between 56 top polygenic scores and disease risk prediction based on medical comorbidities, among cases, excluding controls). The red dashed line at zero highlights a distribution shift to the left.

**A1
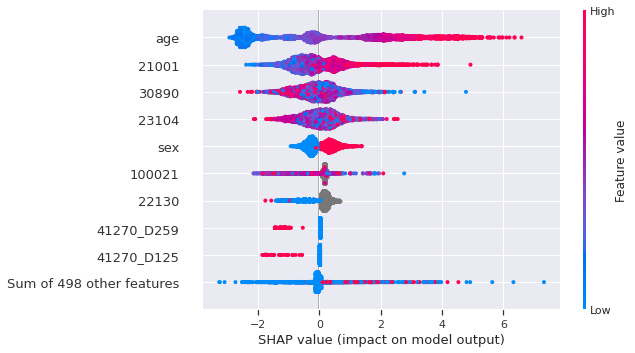
**

**Lenient A1
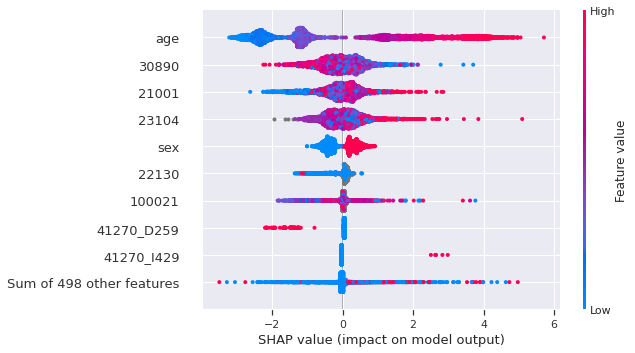
**

**A2
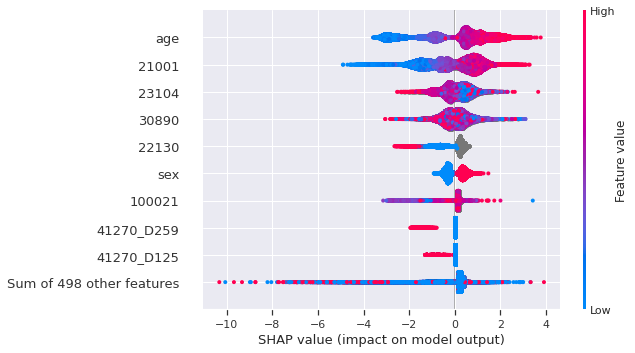
**

**B1
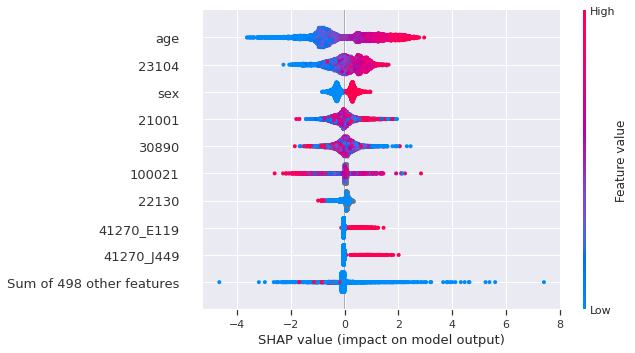
**

**B2
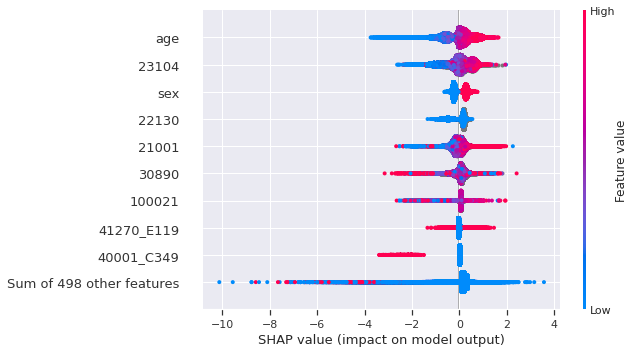
**

**C1
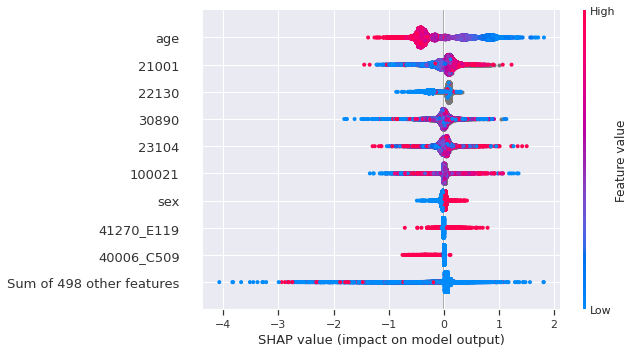
**

**C2
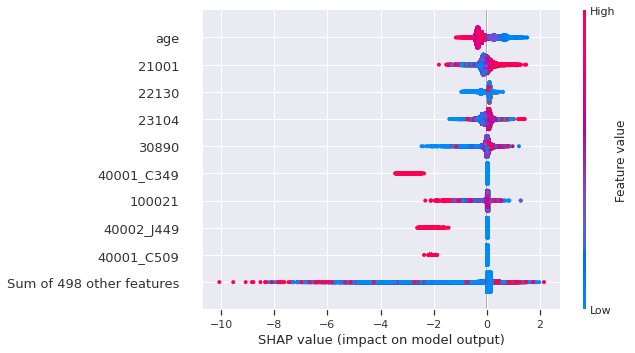
**

**Supplementary Figure S5.** Feature importance plots using SHAP values, for an XGBoost classifier predictive of COVID-19 severity based on medical risk factors. Each subplot corresponds to a binary classification problem of a specific case/control definition (e.g., A1 cases vs A1 controls).
